# Genetic and clinical landscape of *ARR3*-associated MYP26: the most common cause of Mendelian early-onset high myopia with a unique inheritance

**DOI:** 10.1136/bjo-2022-321511

**Published:** 2022-09-30

**Authors:** Yingwei Wang, Xueshan Xiao, Xueqing Li, Zhen Yi, Yi Jiang, Fengsheng Zhang, Lin Zhou, Shiqiang Li, Xiaoyun Jia, Wenmin Sun, Panfeng Wang, Qingjiong Zhang

**Affiliations:** 1 State Key Laboratory of Ophthalmology, Zhongshan Ophthalmic Center, Sun Yat-sen University, Guangdong Provincial Key Laboratory of Ophthalmology and Visual Science, Guangzhou, Guangdong, China; 2 Department of Ophthalmology, Chaoju Inner Mongolia Eye Hospital Co Ltd, Hohhot, China; 3 Department of Ophthalmology, West China Hospital, Sichuan University, Chengdu, Sichuan, China

**Keywords:** Genetics

## Abstract

**Aims:**

To elucidate genetic background of early-onset high myopia (eoHM) and characteristics of *ARR3*-associated MYP26.

**Methods:**

Variants in 14 genes reported to contribute to eoHM, including *ARR3*, were selected from exome sequencing data set and classified into different categories following American College of Medical Genetics and Genomics guidelines based on in silico prediction, associated phenotypes, confirmation and cosegregation analysis. The available clinical data of individuals were summarised.

**Results:**

Pathogenic and likely pathogenic variants in three of 14 genes were identified in 52 of 928 families with eoHM, including 29 in *ARR3*, 22 in *OPN1LW* and 1 in *LRPAP1*. For *ARR3*, 24 pathogenic variants (16 truncation and 8 missense) were identified in 66 women and 12 men, in whom 64 women and 4 men had eoHM by X-linked female-limited inheritance. Refraction ranged from −5.00 to −28.75 diopter (−12.58±4.83). Mild-to-moderately reduced cone responses were recorded in 76.9% (10/13) of patients with electroretinogram recordings. Most patients (75.9%, 41/54) had mild myopic fundus changes (C0 to C1). Genotype–phenotype analysis suggested that the myopic retinopathy degree was correlated with age and the variant’s nature. Peripheral retinal degeneration was observed in 38.5% (5/13) patients using wide-field examinations.

**Conclusion:**

This study reveals *ARR3* as the most frequently implicated gene for Mendelian eoHM. Truncations and highly scored missense variants in *ARR3* are pathogenic. Myopia due to *ARR3* mutations is transmitted in X-linked female-limited inheritance, manifests with mild cone impairment and slowly progresses to pathologic myopia. Identification of the most common cause for Mendelian eoHM provides a valuable starting point into the molecular mechanism of myopia.

**WHAT IS ALREADY KNOWN ON THIS TOPIC**

- Heterozygous variants in *ARR3* lead to early-onset high myopia (eoHM) in a unique X-linked female-limited inheritance.

**WHAT THIS STUDY ADDS**

- Variants in *ARR3* are the most frequent cause of Mendelian eoHM. Truncation and highly scored missense variants in *ARR3* contributed to eoHM, which is characterised by mild cone impairment.

**HOW THIS STUDY MIGHT AFFECT RESEARCH, PRACTICE OR POLICY**

- Revealing the most frequently implicated genes responsible for Mendelian eoHM might shed light on the myopic aetiology and potential targets for interventions.

## Introduction

Pathologic ocular changes associated with high myopia (HM) have become a leading cause of blindness and low vision.^1 2^ Genetic studies on HM, especially early-onset high myopia (eoHM), provide an important avenue for elucidating its underlying molecular basis. In the background of a major contribution of polygenic inheritance, clarifying the contribution of reported monogenic genes in eoHM was also valuable.^3–5^ In particular, mutations in *ARR3* were suggested to be the most common cause of Mendelian eoHM based on our in-house data and a recent study.^6 7^

Our previous study first reported that MYP26—an eoHM transmitted in a unique X-linked female-limited inheritance—is caused by heterozygous variants of *ARR3* (HGNC: 710, OMIM: 301770).^8^ *ARR3* locates at *Xq13.1* and encodes a 388-amino acid cone arrestin, which is mainly expressed in cones.^9^ To date, X-linked female-limited inheritance has been only observed in two diseases—MYP26 due to *ARR3* mutations and epilepsy and mental retardation restricted to women due to *PCDH19* mutations.^8 10^ *ARR3*-associated MYP26 has been further confirmed in other studies.^7 11 12^ Understanding the genetic and clinical landscape of *ARR3*-associated MYP26 is interesting considering that it is a potential most frequently implicated gene for Mendelian eoHM with a unique pattern of inheritance.

In the current study, potentially pathogenic variants (PPVs) in known genes associated with eoHM were selected from 928 families with eoHM collected in our clinic and thoroughly evaluated. Specifically, *ARR3* variants were systemically and strictly analysed. Comparative analysis was performed using three data sets: (1) *ARR3* variants in 6386 subjects with other eye conditions obtained by in-house exome sequencing data, (2) *ARR3* variants in gnomAD and (3) reported *ARR3* variants. Clinical data of patients with PPVs in *ARR3* were summarised and analysed. A total of 24 disease-causing mutations in *ARR3* were identified in 29 families. Genotype and phenotype analysis of data of 78 subjects provided us with a brief genetic and clinical landscape of MYP26. Our data confirmed *ARR3* as the most frequently implicated gene for Mendelian eoHM. Further study on the molecular pathogenesis of eoHM caused by mutant cone-specific arrestin may shed light on the mechanisms of myopia related to abnormal cone signals, especially common myopia.

## Method

### Subjects

This study was approved by the institutional review board of the Zhongshan Ophthalmic Center. In total, data of 928 families with eoHM were collected in the Pediatric and Genetic Clinic at the Zhongshan Ophthalmic Center, Guangzhou, China. All probands from these families were initially considered as isolated eoHM based on the initial complaint and routine examination at the out-patient clinic (some of them have been shown to be at the early stage of other diseases, such as syndromic high myopia, revealed by longitudinal observation or further specific examinations, as shown in our previous studies).^13–15^ An additional 6386 individuals with different eye conditions, including retinitis pigmentosa, glaucoma and others and subjects with normal eyes, served as controls. Written informed consent consistent with the tenets of the Declaration of Helsinki was obtained from the participants or their guardians before the collection of peripheral venous blood samples and clinical data. The genomic DNA was obtained using a previously described method.^16^

### Sequencing analysis

Whole-exome sequencing (WES) and targeting exome sequencing (TES) of genomic DNA samples were performed as previous study.^16^ Of the 928 families with eoHM and 6386 control families, at least the proband of each family underwent WES or TES, and some families had trio-based WES/TES or linkage analysis. Variants in the 14 genes reported to contribute to eoHM, including *OPN1LW*, *SCO2*, *ZNF644*, *CCDC111*, *LRPAP1*, *SLC39A5*, *P4HA2*, *ARR3*, *BSG*, *DZIP1*, *XYLT1*, *NDUFAF7*, *CPSF1* and *TNFRSF21*,^6 17^ were collected from exome sequencing data sets. To obtain a landscape view of variants in the 14 genes in all 928 families, all previously described data, including one family in *LRPAP1*,^18^ four families in *OPN1LW* 19 and three families in *ARR3*,^8^ were included in the current study.

Variants were filtered by multistep bioinformatics following previously described procedures.^20^ First, all variants were annotated with the allele frequency in the general population using the gnomAD database (http://gnomad.broadinstitute.org/). The variants with allele frequencies more than 0.01 were excluded. Missense variants were evaluated in two ways, that is, effects on splicing by three tools described later and effects on protein structure and function predicted by five in silico tools: SIFT http://sift.jcvi.org/www/SIFT.enstsubmit.html), PolyPhen-2 (http://genetics.bwh.harvard.edu/pph2/index.shtml), PROVEAN (https://provean.jcvi.org/genome_submit_2/), REVEL (https://sites.google.com/site/revelgenomics/) and CADD (https://cadd.gs.washington.edu/). The splicing influence of variants was predicted by three tools: BDGP (http://www.fruitfly.org/ (in the public domain)), HSF (https://hsf.genomnis.com) and NetGene2 (http://www.cbs.dtu.dk/services/NetGene2/). Subsequently, the missense and truncation variants in genes responsible for eoHM were excluded as PPVs if they were evenly distributed in different groups (including normal controls) but not clustered in eoHM by comparative analysis of large data sets (some reported variants in most of the 14 genes, as those seen in *FSCN2* and *RCBTB1*,^21 22^ were considered to be likely benign or to have uncertain significance and were filtered out by this stringent strategy). The associated phenotypes were analysed, and cosegregation analysis was performed to evaluate the pathogenicity of individual variants. Candidate pathogenic variants in *ARR3* were finally validated by Sanger sequencing. Finally, all variants were defined according to the guidelines of the American College of Medical Genetics and Genomics and the Association for Molecular Pathology (ACMG/AMP).

### Phenotype summarisation

The available clinical data of individuals with PPVs in *ARR3* were sorted. The criteria of eoHM were defined as a refractive error of ≤−5.0 diopter (D) (or axial length ≥25 mm) in children under 7 years old or ≤−6.0D (or axial length ≥26 mm) in persons with age over 7 years old who complained of HM before 7 years old but examined after 7 years old. Clinical data were summarised, and data included the best-corrected visual acuity (BCVA), refraction recording, axial length, fundus photography, scanning laser ophthalmoscope, optical coherence tomography (OCT) and electroretinogram (ERG) following the International Society for Clinical Electrophysiology of Vision (ISCEV). The high myopic fundus was classified according to the META-analysis for Pathologic Myopia (META-PM) classification.^23^

### Statistical analysis

Statistical analysis was performed by SPSS Statistics V.25.0, and statistically significant was verified when the p value was less than 0.05. *Fisher’s* exact test was performed to evaluate the distribution differences of PPVs in families with eoHM versus families with other eye conditions or those in the general population, phenotypes differences between affected women and men and genotype-phenotype correlations. A Bonferroni correction was used when comparing the distribution of PPVs in 14 genes. The intraclass correlation coefficient (ICC) was performed to assess the correlation of refraction between eyes. The *t* test was used to evaluate the differences in visual acuity between affected women and men.

## Results

### Genetic spectrum and variants identification

**Figure 1:**
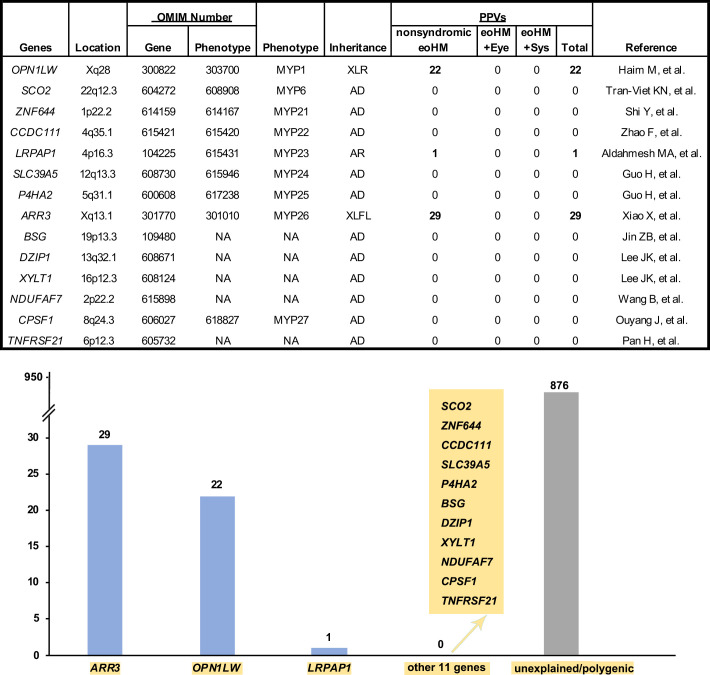
Genetic landscape of Mendelian eoHM based on our in-house 928 families with eoHM. The upper table and column chart below shows the count of in-house families in whom pathogenic or likely pathogenic variants in the 14 reported causative genes for nonsyndromic eoHM were identified. AD, autosomal dominant; AR, autosomal recessive; eoHM, early-onset high myopia; eoHM+Eye, early-onset high myopia with other eye conditions; eoHM+Sys, early-onset high myopia with systematical dystrophy; NA, not available; PPVs, pathogenic or likely pathogenic variants; XLR, X-linked recessive; XLFL, X-linked female-limited.

PPVs were identified in 52 families with eoHM, including 29 families in *ARR3*, 22 in *OPN1LW* (18 with LVAVA haplotype, 4 with c.739C>T/ p. Arg247*, c.417_418insGGTCTCT, c.519_520insCCCTG and c.617_620dup, respectively),^19^ and one in *LRPAP1*,^18^ all were significantly clustered in families with eoHM. No specific variants in the remaining 11 genes were clustered in families with eoHM, including a specific class of variants, variants in a specific region or variants with overall high predicted scores based on multiple tools. Some known variants in the 11 genes were classified as likely benign or variants of uncertain significance for their equal distribution in controls and the general population based on comparative analysis of large data sets or they were tolerable in the general population without any significant enrichment in the large data sets (figure 1, online supplemental tables s1 and s2).

10.1136/bjo-2022-321511.supp6 Supplementary data

10.1136/bjo-2022-321511.supp7 Supplementary data

**Figure 2:**
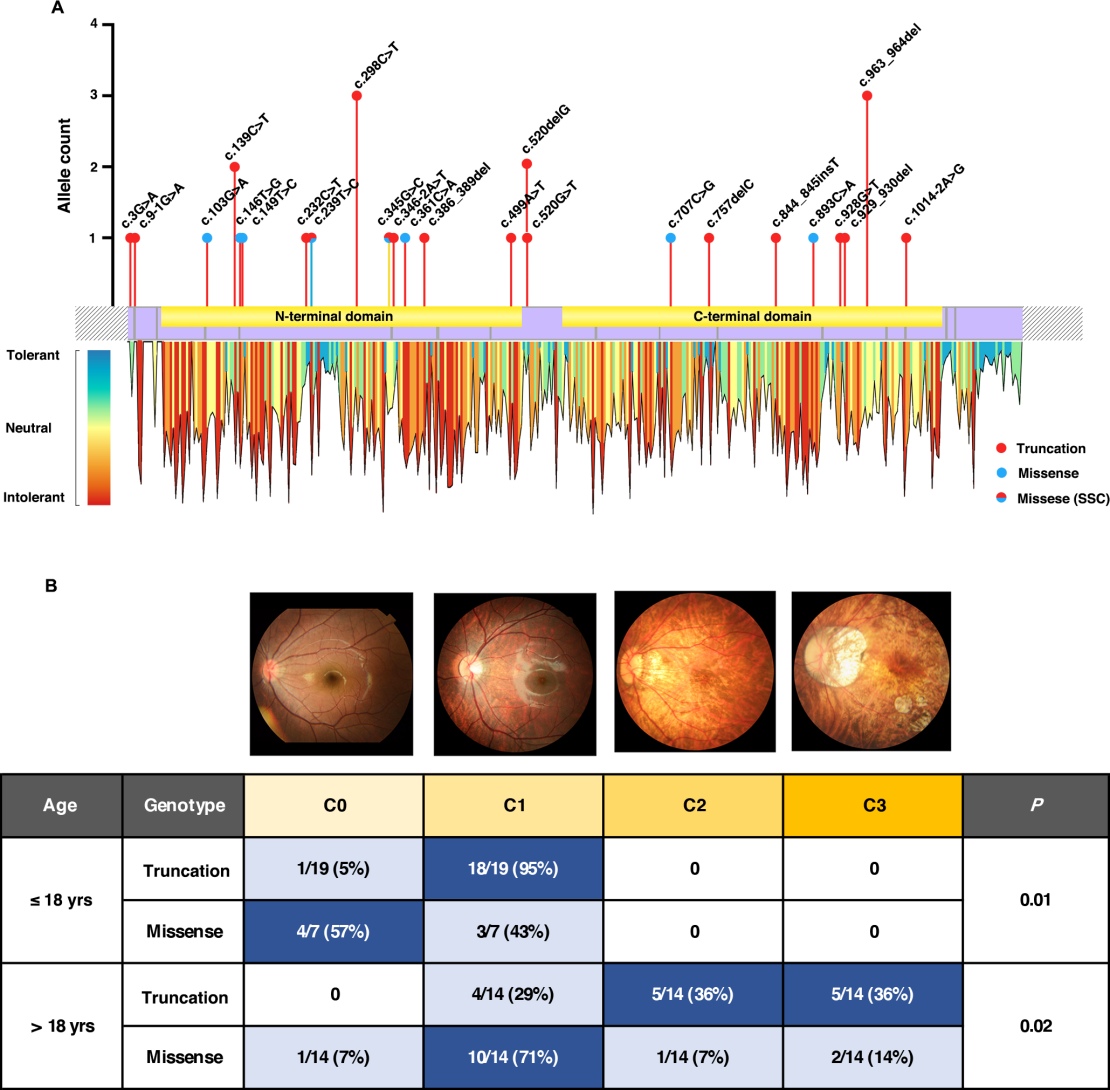
Comprehensive genetic and phenotypic analysis of *ARR3*-associated MYP26. (A) The distribution and allele count of the 24 pathogenic variants in *ARR3* identified in our cohort. To get a bird’s-eye view of variant effects across the entire *ARR3* gene, possible changes of every residue (NM_004312.2; UniProt: P36575) were sequentially predicted by the five in-silico missense prediction tools. The tolerance of mutation at each amino acid was calculated by summing up the score of each potential change and then ranked to draw a tolerance landscape. The tolerance landscape was used as a reference to assess variants in *ARR3*. The position and allele number of all in-house pathogenic variants are located above the mRNA structure diagram. Truncating mutations are presented as red dots, while missense variants are blue. Two missense variants (c.239C>T and c.345G>C) were predicted to cause splicing site changes and are marked with both colours. The bar colours for each missense variant represent the residue pathogenicity and tolerance according to the heatmap below, and all truncation variants are shown as red bars. (B) Genotype-phenotype correlation matrix based on the fundus images of 54 in-house patients with *ARR3* variants. All individuals younger than 18 presented with C0 or C1 fundus changes, and patients with truncation variants were more likely to exhibit C1 compared with those with missense variants (p=0.01). With progression, pathogenic myopic lesions could be observed in patients older than 18, in whom truncation variants led to severer consequences (C2 and C3) than missense variants with statistical differences (p=0.02). SSC, splicing site change.

A total of 72 rare variants (29 missense, 15 synonymous, 14 splicing regions’ changes, 6 nonsense, 6 frameshift, 1 start loss and one 5′-UTR alternation) in *ARR3* were detected. Among the 72 variants, 24 were classified as PPVs (online supplemental table s3, figure 2A), including eight missense and 16 truncation variants (six nonsense, six frameshift, three splice-site acceptor and one start loss). Of the 24, 21 were novel. Of the eight missense variants, six were predicted to be damaged by at least four in silico tools, and two (c.239T>C and c.345G>C) were predicted to be damaged by one or two tools and also affect splicing. Two-thirds of the PPVs in *ARR3* (66.7%, 16/24), were truncations, distributed across the entire coding frame without significant clustering in individual exons. All 16 truncation variants were detected in 21 of 928 families with eoHM but in none of the 6386 control families (p=1.84E-19). Compared with the relatively rare frequency of *ARR3* truncation variants in the gnomAD database, truncation variants are significantly clustered in eoHM families (p=1.43E-45) (online supplemental figure s1).

10.1136/bjo-2022-321511.supp8 Supplementary data

10.1136/bjo-2022-321511.supp1 Supplementary data

**Figure 3:**
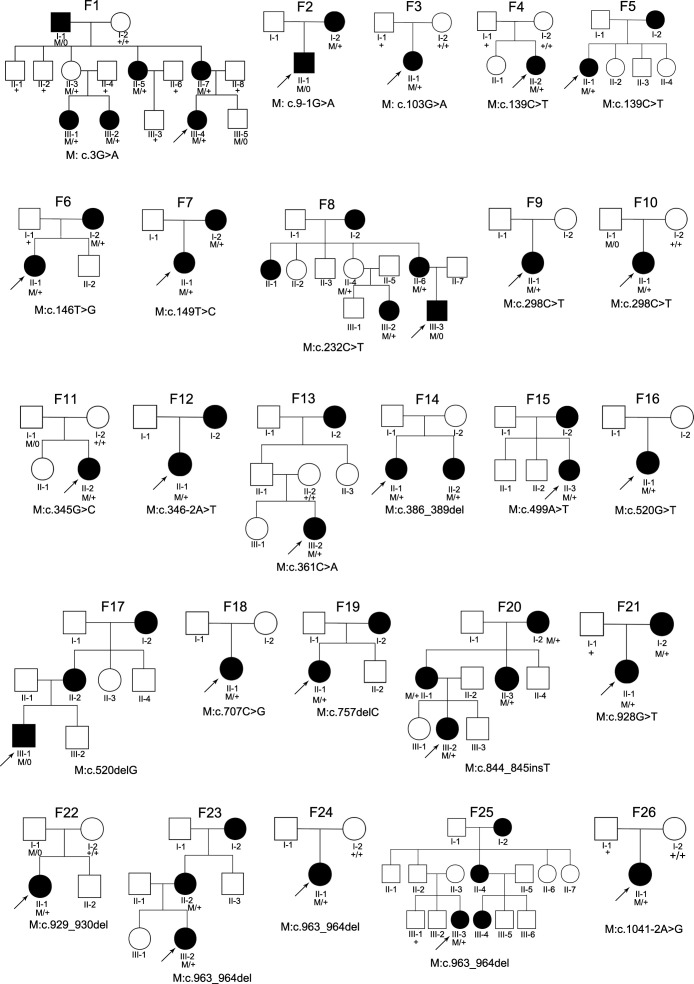
The pedigrees of 26 newly identified families with pathogenic variants in *ARR3*. Male individuals are represented with squares, while female individuals are shown as circles. The shading indicates an affected patient. The proband in each family is indicated by arrows. Successive family numbers are on the top of the pedigrees while variants are listed under the pedigrees.

Of the 29 families with *ARR3* variants, an average of four individuals was sequenced per family. Totally, 78 individuals in the 29 families (26 new families) harboured the 24 PPVs in *ARR3* (figure 3, online supplemental figure s2), including 66 heterozygous women and 12 hemizygous men, in whom eoHM was presented in 97.0% (64/66) women and 33.3% (4/12) men. Of the 29 families, *ARR3* mutations were de novo in three families (F3, F4 and F26), transmitted from affected women to affected women in 14 families, from unaffected male carriers to affected women in three families (F10, F11 and F22), from affected women to affected men in three families (F2, F8 and F17), and of unknown origin in six families (F9, F13, F14, F16, F18 and F24). The transmission of eoHM in these families conformed to X-linked female-limited inheritance, contrary to classic X-linked traits (online supplemental table s4).

10.1136/bjo-2022-321511.supp2 Supplementary data

10.1136/bjo-2022-321511.supp9 Supplementary data

### Clinical characterisation of patients with *ARR3* variants

Among the 78 individuals with variants in *ARR3*, 68 were affected (64 women and 4 men), and 10 individuals (8 men and 2 women) were unaffected. All the 68 affected patients complained about near vision, but no photophobia. Sixty-two patients (58 women and 4 men) had available refraction data, the other 5 women only had axial length, and one complained about eoHM without detailed clinical information (online supplemental table s5). The bilateral refraction recordings showed a strong correlation (ICC>0.75), which could be averaged by following conventional statistics. The spherical equivalent refraction ranged from −5.00 D to −28.75 D (−12.58D±4.83D) (online supplemental figure s3A). Based on follow-up data of eight affected women and the trend line fitted out by all patients’ refraction data, the *ARR3*-associated MYP26 presented with the trend of rapid progression at first followed by slow increase (online supplemental figure s3A,B). Astigmatism ranged from −0.25 D to −6.00 D (−2.63 D±1.32D) (online supplemental figure s3C). BCVA, measurable in 57 patients (53 women and 4 men), ranged from 0.01 to 1.20 (median 0.40, decimal). Approximately, 82.5% (47/57) of patients had BCVA of no less than 0.3 at initial. Of the remaining 10 patients with BCVA of less than 0.3, four had vitreous opacity or age-related cataract and three were too young to get BCVA examined (online supplemental figure s3D).

10.1136/bjo-2022-321511.supp10 Supplementary data

10.1136/bjo-2022-321511.supp3 Supplementary data

**Figure 4:**
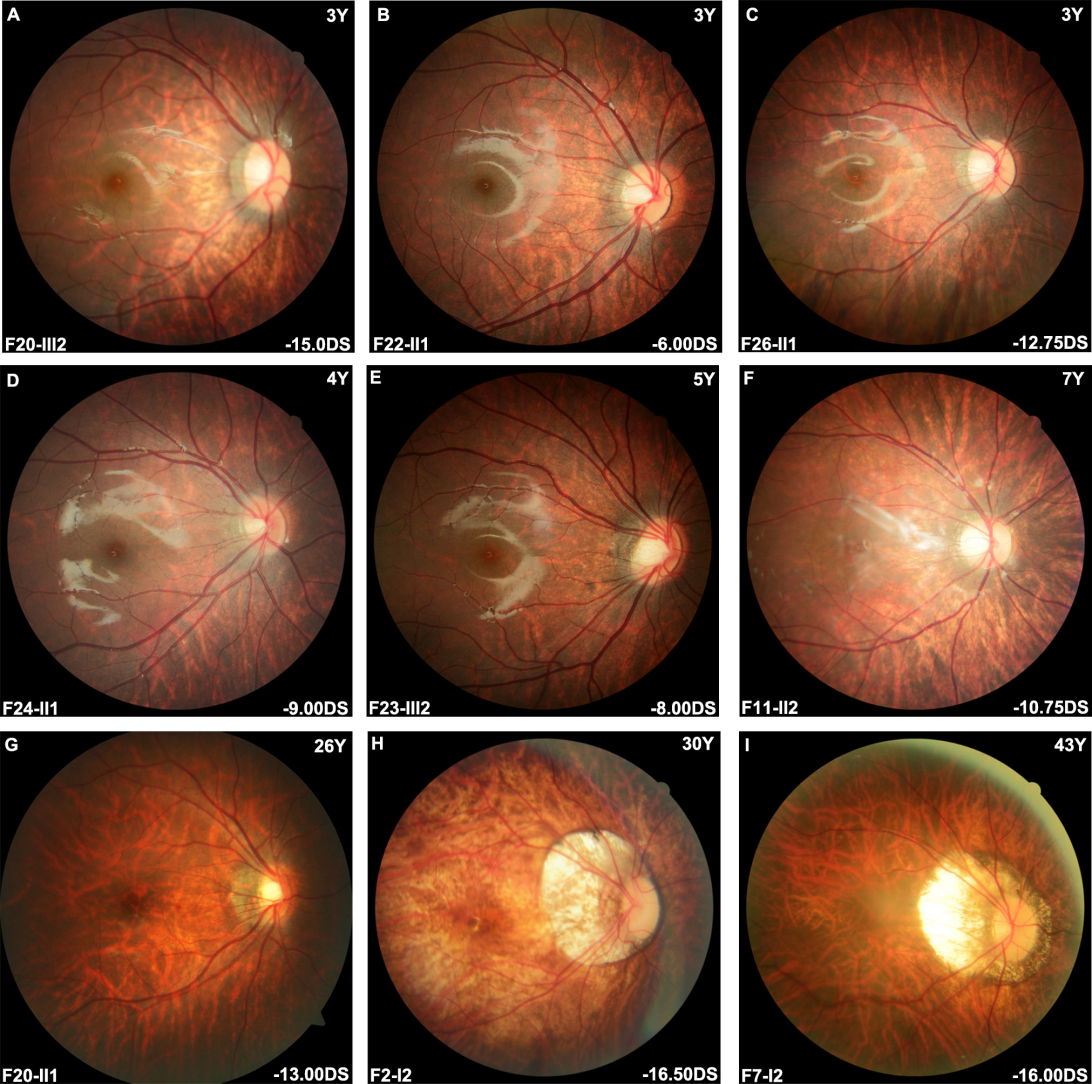
Posterior fundus imaging of female patients with heterozygous *ARR3* variants. (A–F) Most in-house patients with heterozygous *ARR3* variants exhibited a simple high myopia tessellated fundus with or without peripapillary crescent enlargement. Macula foveal area was usually relatively preserved, and chorioretinal atrophy was rare among young individuals. (G–I) Some in-house adult patients showed mild tessellated fundus changes as young individuals, whereas most elder patients presented with diffuse or patchy chorioretinal atrophy, accompanied by further enlarging peripapillary crescent.

Fundus images, available for 50 affected women and four affected men, were classified into four categories (C0, C1, C2 and C3) (figure 4).^23^ Relatively normal posterior fundus without myopic retinal degeneration (C0) was observed in six women (11.1%, 6/54). Almost two-thirds of patients (32 women and 3 men; 64.8%, 35/54) showed high myopic tessellated fundus with clearly visible choroidal vessels in the posterior area (C1). Yellowish white fundus due to diffuse chorioretinal atrophy (C2) was observed in five women and one man (11.1%, 6/54), whose atrophic area was restricted to the optic disc in three and extended to the macula area in three. Well-defined patchy white atrophy (C3) was observed in seven women (13.0%). None had the macular atrophy lesions (C4) involving the central fovea. Apart from macular changes, peripapillary crescent enlargement was observed in 40 affected women and three affected men (79.6%, 43/54) and was measurable in 36 patients, in whom 18 patients (16 women and 2 men) had crescent shorter than 1 papillary diameter (PD) and 18 patients (17 women and 1 man) had crescent larger than 1 PD. Posterior staphyloma was observed in 33 of 55 patients, including 30 affected women and three affected men. According to Curtin’s classification,^24^ posterior staphyloma in 16 women and 2 men (18/33, 54.5%) involved the macula area, including six women with type I and 12 with type II. Posterior staphyloma not including the macula was observed in 14 women and 1 man (15/33, 45.5%), in which 11 women and 1 man showed peripapillary (type III) and two involved the inferior area (type V).

**Figure 5:**
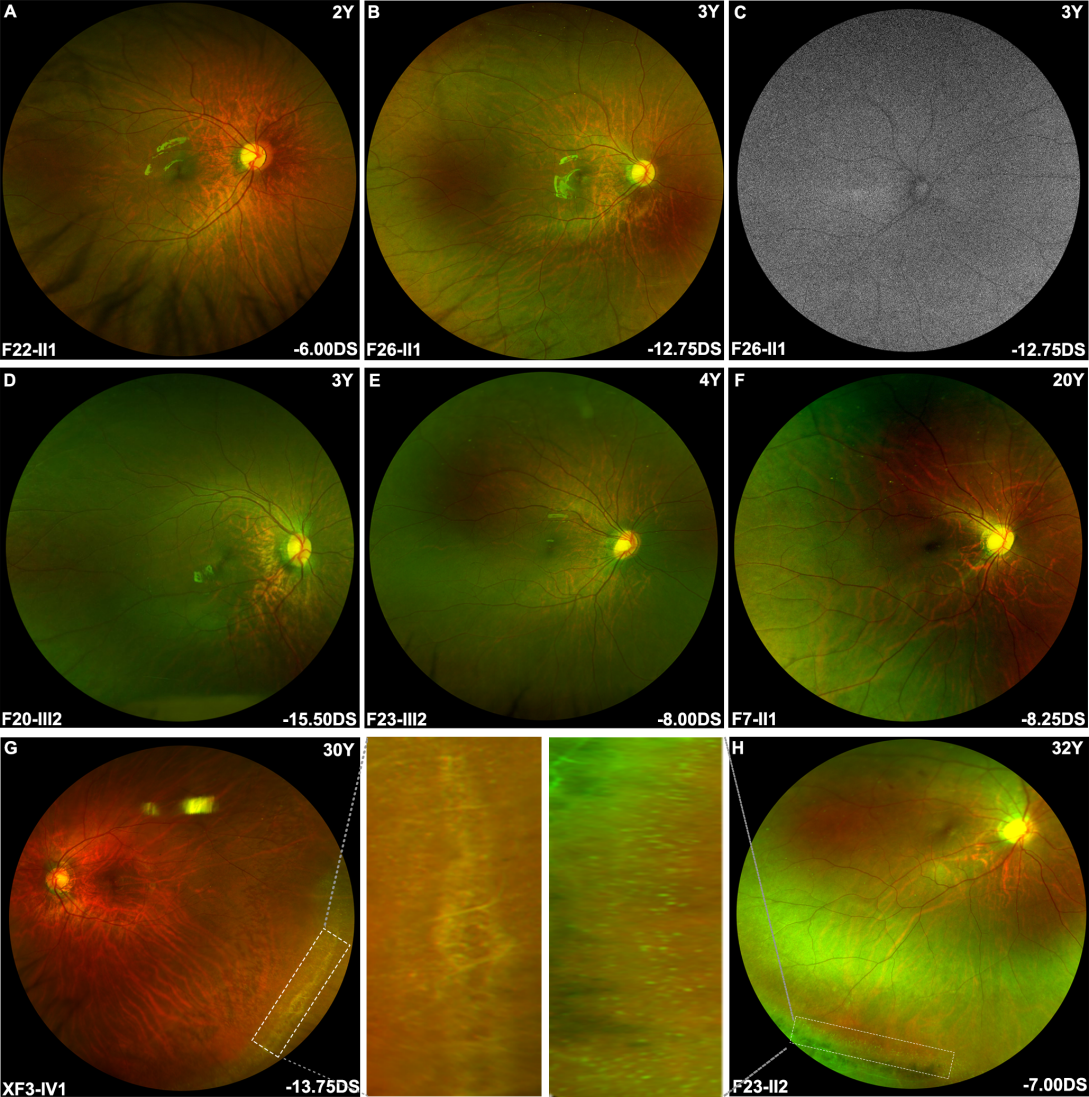
Wide-field fundus photography of in-house patients with heterozygous *ARR3* variants. (A–F) More than half of in-house patients presented with tessellated fundus to various extents without obvious retinal degeneration in the peripheral area. There was no autofluorescence abnormality in the fundus autofluorescence examination (C). (G–H) The appearance of specific white dots and lattice retinal degeneration in the peripheral retina area was mostly observed among elder female patients in our cohort. The middle parts of the two images correspond to magnified images of the peripheral retinal degeneration area.

Wide-field fundus examinations were available in 13 affected women, among whom 5 women older than 20 years old had various peripheral retinal degenerations; three patients had myopic holes in the periphery and two with lattice degeneration. The remaining eight children had preserved peripheral retinas. No conspicuous hyper-or-hypo-autofluorescence lesions were observed in the whole retina (figure 5). Normal foveal structure was observed in 21 affected women and two affected men (85.2%, 23/27) with OCT. Myopic maculopathy change was found in the remaining four, in whom one woman had atrophy, two showed tractions and one had neovascularisation change (online supplemental figure s4). Twelve affected women and one affected man had ERG, including three women (23.1%, 3/13) had normal photopic and scotopic responses, five women and one man (46.2%, 6/13) showed normal scotopic responses but mild-to-moderately decrease a-wave and b-wave amplitude in cones, four women (30.8%, 4/13) showed mild-to-moderately decreased b-wave amplitude in both rods and cones and decreased a-wave amplitude in cones (online supplemental figure s5). Nine affected women and one man from four families had colour vision tests. All had a normal colour vision. No significant difference was observed for phenotypes, including refraction, BCVA, fundus manifestation, structural and functional changes, between affected women and men (p>0.05).

10.1136/bjo-2022-321511.supp4 Supplementary data

10.1136/bjo-2022-321511.supp5 Supplementary data

### Genotype–phenotype correlation of *ARR3*

Fundus images of the 54 patients were split into two groups by age: ages below or over 18 years old (figure 2B). Advanced categories (C2 and C3) were only observed in patients older than 18 years old, whereas early categories (C0 and C1) were more common among patients younger than 18 compared with those over 18, with statistical significance (p<0.0001). Of the 26 patients with age equal to or less than 18 years, all had milder categories of maculopathy (C0 or C1), and most patients with truncating mutation (94.7%,18/19) had C1, while the majority with missense (57.1%, 4/7) had C0 (p=0.01). Among the 28 patients with ages older than 18 years, almost half of them (13/28, 46.4%) presented with advanced myopic macular change, in whom majority with truncating mutation (71.4%, 10/14) showed severer categories of maculopathy (C2 or C3), whereas most patients with missense variants (78.6%, 11/14) had relatively milder maculopathy (C0 or C1) (p=0.02).

## Discussion

This study sheds light on the genetic background of eoHM based on a large data set of 928 families with eoHM, of which *ARR3* is the most frequently implicated gene by a unique X-linked female-limited inheritance, out of the 14 genes investigated. The large case series of 29 families with *ARR3* mutations provide valuable recognition of the special *ARR3*-associated MYP26. All truncations and highly scored missense mutations in *ARR3* were intolerant and caused eoHM with cone impairment in unique X-linked female-limited mode. Overall, our study largely expanded our knowledge of eoHM, especially *ARR3*-associated MYP26, which will bring a strong impact on a broad audience.

Non-syndromic HM is diagnosed by a high degree of refraction (<−6.0D) and an exclusion process of abnormality involving other ocular segments or other systems, which is the diagnosis requirement of syndromic HM. Precise ophthalmology and whole physical check-up are essential in the differential diagnosis of syndromic and non-syndromic HM. Pathologic myopia is not always occurred with HM and is diagnosed by the presence of posterior structural changes (posterior staphyloma or myopic maculopathy). It has been identified that eoHM might be accompanied by reduced scotopic and photopic amplitudes, which was strongly correlated with the degree of myopia or myopic maculopathy in the late stage.^25 26^ Different from cone dystrophy, patients with *ARR3*-associated MYP26 usually presented with reports of near vision and myopia diagnosis by routine examinations, but no photophobia or colour blindness, which is characteristics of cone dystrophy. Macular involvement earlier occurred in cone dystrophy than *ARR3*-related myopic maculopathy. The cone involvement in ERG of patients with MYP26 and the strong association between the underlying pathogenic mechanism of *ARR3* and cones suggested that *ARR3*-associated MYP26 and cone dystrophy are distinct but not completely separate. For the absence of completely ophthalmic examinations from some patients and not enough follow-up data for each patient, considering disease progression and phenotype variability, whether the MYP26 is non-syndromic or syndromic is eager to be clarified in more patients with detailed tests.

HM has drawn wide public attention for its increasing prevalence, poor understanding of pathogenesis mechanism and complex interaction.^1^ Identification of causative genes and exploration of underlying pathogenesis mechanism will open new avenues for potential intervention. A recent study found that *ARR3* mutations were responsible for 5% of cases and concluded as the most common cause of eoHM.^7^ Based on our cohort of eoHM, *ARR3* was the most frequently implicated gene for Mendelian eoHM (~3.1%), and *OPN1LW* ranked the second (~2.4%). Cone-specific expression of both *ARR3* and *OPN1LW* reminds cones to play an important role in the development of eoHM.^8 27^ Complex interactions involving numerous pathways for myopia development have been raised.^28^ A meta-analysis revealed that the light-dependent retina-to-sclera signalling cascade is an essential trigger of refractive error.^29^ A ‘contrast hypothesis’ of *OPN1LW* though that mosaics status of cones with variable amount of photopigment leads to abnormal contrast and stimulates of eyeball growth.^30^ Recently, an *ARR3* related cone-associated hypothesis postulated that X-arrestin dystrophy in long and medium (LM) cones results in more sensitive function-to-colour stimuli, leading to higher luminance contrast and elongation of eyeballs.^11^ The exact underlying mechanisms of both *ARR3* and *OPN1LW* are unknown. Functional studies that provide insights into the molecular pathogenesis of cone-dysfunctional-related eoHM may shed light on effective intervention for eoHM. The absence of evidence of the other 11 genes with eoHM was based on updated criteria at the individual gene level, which might be explained by interaction effect with other factors, susceptible genetic contribution, incomplete penetrance and polygenic inheritance patterns of high myopia, which need to be uncovered in further research.

Recently, a man with a nonsense variant in *ARR3* was found to display eoHM, which reminded the non-zero penetrance of MYP26 in men.^12^ Four affected men and two unaffected women in this study revealed the non-zero penetrance of MYP26 in hemizygous men (~33.3%) and not the 100% penetrance in women (~97.0%). The female-to-male sex ratio could be up to ~20:1, considering affected members without genotype. Female carriers of several genes in X-linked recessive traits exhibit the identical or milder phenotype than affected men, such as *RPGR*, *FRMD7* and *GPR143*.^31^ These men with *ARR3* variants and eoHM might provide insights into the mechanisms of the unique X-linked female-limited inheritance. The X-inactivation mechanism results in a somatic mosaicism cell status, in which mutant and wild-type cells coexist, mutually compete, and lead to unique female-limited inheritance.^32^ Affected men with mental retardation restricted to women due to *PCDH19* mutations were also identified and were explained by the mosaicism status mimicking the cellular interference pathogenic mechanism of women,^33^ which has been tested by sophisticated mouse models.^34^ A few unaffected women might be explained by the irregular dominance, defects in other unknown hyperopia-associated genes or bidirectional regulation mechanisms.^35^ Ten unaffected individuals (8 men and 2 women) with mutations did not report of other clinical symptoms, had refraction no more than −6.0D, and normal fundus manifestations as well as retinal structure. It is unknown whether they had functional impairment even though the normal fundus manifestation and whether there are abnormalities in far-periphery that is hard to find in routine posterior photography, which needs more attention in future studies.

In conclusion, our study enriched our knowledge regarding eoHM, especially *ARR3-*associated MYP26 with cone involvement, which develops into pathologic myopia with age. The nature of variants might affect the progression and be an important prognostic decider, in which truncation variants result in a severer phenotype. The potential pathogenesis mechanism of *ARR3*, the most frequently implicated genes responsible for Mendelian eoHM, might provide new insights into the myopic aetiology as well as additional underlying targets for therapeutic interventions. Furthermore, confirmation of the unique X-linked female-limited inheritance highlights the underlying genetic defects for additional hereditary diseases and may be a significant breakpoint to solve more problems in inherited diseases of unknown genetic defects.

## Data Availability

Data are available upon reasonable request.
