# Supplementary material for: Genetic and clinical landscape of ARR3-associated MYP26: the most common cause of Mendelian early-onset high myopia with a unique inheritance
Source: Br J Ophthalmol. 2022 Sep 30;107(10):1545–53. doi: 10.1136/bjo-2022-321511 (PMC10579186; doi:10.1136/bjo-2022-321511)
Supplement: Supplementary data [file bjo-2022-321511supp006.pdf]

BMJ Publishing Group Limited (BMJ) disclaims all liability and responsibility arising from any reliance placed on this supplemental material which has been supplied by the author(s)

Supplementary Table 1. Variants identified in the 11 genes responsible for non-syndromic eoHM based on our cohort.

chr#	Gene	Position	Nucleotide Change	Effect	sample count	Diagnosis		gnomAD		SIFT	PROVEAN	Polyphen-2	CADD	REVEL	HGMD
						eoHM	Others	Count	Allele Frequency						
chr22	*SCO2*	50962077	c.764G>A	p.R255Q	1	0	1	11	3.89E-05	T(0.565)	N(-0.62)	B(0.053)	11.16	0.235	
chr22	*SCO2*	50962089	c.752C>T	p.S251L	29	4	25	48	0.000169785	D(0.028)	N(-2.16)	B(0.05)	15.17	0.148	
chr22	*SCO2*	50962117	c.724G>A	p.G242S	1	0	1	1	3.18E-05	T(0.051)	D(-4.69)	P(0.758)	15.93	0.369	
chr22	*SCO2*	50962128	c.713C>T	p.T238M	2	0	2	37	0.000130835	T(0.088)	N(1.17)	P(0.512)	22.6	0.401	
chr22	*SCO2*	50962165	c.676A>G	p.I226V	1	0	1	1	3.97722E-06	T(0.067)	N(-0.78)	B(0.026)	16.4	0.326	
chr22	*SCO2*	50962177	c.664G>A	p.V222M	2	0	2	9	3.18226E-05	D(0.001)	D(-2.88)	D(0.994)	26.3	0.774	
chr22	*SCO2*	50962222	c.619G>A	p.V207M	1	0	1	14	4.9505E-05	D(0)	D(-2.94)	D(0.999)	24	0.802	
chr22	*SCO2*	50962264	c.577G>A	p.G193S	1	0	1	8	3.18281E-05	D(0)	D(-5.89)	D(1)	24.1	0.831	
chr22	*SCO2*	50962300	c.541G>A	p.V181I	5	1	4	22	7.78243E-05	T(0.165)	N(-0.64)	B(0.262)	15.42	0.474	
chr22	*SCO2*	50962306	c.535C>T	p.R179C	1	0	1	13	4.59936E-05	D(0.004)	D(-4.68)	D(0.969)	24.6	0.639	
chr22	*SCO2*	50962321	c.520G>A	p.V174I	2	0	2	2	7.96248E-06	T(0.245)	N(-0.86)	B(0.014)	13.79	0.388	
chr22	*SCO2*	50962395	c.446G>A	p.R149Q	1	0	1	14	4.95642E-05	T(0.263)	N(1.04)	B(0.005)	6.637	0.16	
chr22	*SCO2*	50962434	c.407T>C	p.I136T	2	2	0	/	/	D(0.001)	D(-4.48)	D(0.99)	26.2	0.937	
chr22	*SCO2*	50962455	c.386G>T	p.G129V	3	1	2	1	3.98206E-06	D(0)	D(-8.44)	D(1)	26.8	0.82	
chr22	*SCO2*	50962458	c.383T>A	p.F128Y	1	0	1	/	/	D(0)	D(-2.89)	D(0.998)	27	0.929	
chr22	*SCO2*	50962482	c.359G>A	p.R120Q	1	0	1	6	2.12681E-05	T(0.113)	N(-0.37)	B(0.021)	19.59	0.262	
chr22	*SCO2*	50962483	c.358C>T	p.R120W	3	1	2	1	3.98765E-06	D(0.04)	N(-1.17)	P(0.881)	26.7	0.83	
chr22	*SCO2*	50962492	c.349G>A	p.A117T	1	0	1	/	/	T(0.239)	N(-0.2)	B(0.333)	18.92	0.225	
chr22	*SCO2*	50962500	c.341G>A	p.R114H	1	0	1	212	0.000752269	D(0.019)	D(-3.47)	D(0.958)	25.7	0.62	Khanh-Nhat TV, et al.
chr22	*SCO2*	50962507	c.334C>T	p.R112W	7	1	6	14	4.96789E-05	D(0.003)	D(-3.52)	P(0.567)	23	0.74	
chr22	*SCO2*	50962514	c.327C>G	p.H109Q	1	0	1	6	2.39674E-05	D(0.049)	D(-4.57)	P(0.51)	14.33	0.365	

chr22	*SCO2*	50962549	c.292G>T	p.A98S	1	0	1	1	4.01532E-06	T(0.18)	N(-1.88)	B(0.034)	10.03	0.124	Tomotaka W, et al.
chr22	*SCO2*	50962551	c.290C>T	p.A97V	7	0	7	7	2.81253E-05	T(0.35)	N(-1.75)	P(0.679)	17.42	0.306	
chr22	*SCO2*	50962612	c.210_229del	p.L71Pfs*4	2	0	2	/	/	NA	NA	NA	NA	NA	
chr22	*SCO2*	50962627	c.214G>A	p.G72S	5	0	5	7	2.55199E-05	D(0.0490	N(0.3)	B(0.045)	9.33	0.215	
chr22	*SCO2*	50962652	c.189C>G	p.I63M	2	0	2	2	8.25962E-06	D(0.032)	N(-1.29)	B(0.146)	13.35	0.223	
chr22	*SCO2*	50962719	c.122A>C	p.Q41P	1	0	1	1	3.1953E-05	T(0.079)	N(0.1)	B(0.001)	8.088	0.26	
chr22	*SCO2*	50962786	c.55C>T	p.P19S	2	0	2	1	4.24203E-06	D(0.035)	N(0.45)	B(0.001)	2.265	0.132	
chr22	*SCO2*	50962802	c.39delG	p.R13Sfs*21	1	0	1	/	/	NA	NA	NA	NA	NA	
chr01	*ZNF644*	91382402	c.3937A>G	p.I1313V	1	0	1	34	0.000120207	D(0.002)	N(-0.14)	P(0.748)	23.1	0.187	
chr01	*ZNF644*	91383615	c.3785G>A	p.R1262Q	1	0	1	1	3.18431E-05	D(0.006)	N(-1.57)	D(0.919)	26	0.311	
chr01	*ZNF644*	91403051	c.3679A>G	p.M1227V	1	0	1	8	2.83531E-05	T(0.122)	N(-0.57)	B(0)	15.97	0.168	
chr01	*ZNF644*	91403111	c.3619G>A	p.V1207I	1	0	1	12	4.25786E-05	T(0.077)	N(-0.24)	B(0.191)	19.01	0.152	
chr01	*ZNF644*	91403179	c.3551G>T	p.R1184M	8	1	7	13	5.18763E-05	T(0.072)	N(-0.31)	B(0.006)	21.8	0.229	
chr01	*ZNF644*	91403191	c.3539T>C	p.M1180T	1	0	1	/	/	D(0.008)	N(-0.29)	B(0.014)	22.2	0.208	
chr01	*ZNF644*	91403435	c.3295C>T	p.R1099C	1	1	0	9	3.18961E-05	D(0.0)	N(-2)	D(0.993)	30	0.252	Zheng YH, et al.
chr01	*ZNF644*	91403464	c.3266A>G	p.Y1089C	33	1	32	65	0.000230216	D(0.0)	N(-2.24)	D(0.982)	24.4	0.235	
chr01	*ZNF644*	91403469	c.3261A>C	p.E1087D	1	0	1	/	/	D(0.007)	N(-0.89)	B(0.001)	19.17	0.338	Cai XB, et al.
chr01	*ZNF644*	91403597	c.3133G>T	p.G1045C	2	0	2	/	/	D(0.0)	N(-1.66)	D(0.996)	31	0.192	
chr01	*ZNF644*	91403626	c.3104C>A	p.T1035N	1	1	0	/	/	T(0.137)	N(-0.53)	B(0.026)	21.7	0.064	
chr01	*ZNF644*	91404080	c.2831C>T	p.A944V	1	1	0	3	1.19822E-05	D(0.001)	N(-0.79)	B(0.001)	21.5	0.183	
chr01	*ZNF644*	91404107	c.2804A>G	p.D935G	1	0	1	1	3.99642E-06	T(0.148)	N(-1.1)	B(0.018)	22.0	0.201	
chr01	*ZNF644*	91404126	c.2785T>A	p.F929I	1	1	0	/	/	D(0.001)	N(-0.49)	B(0.205)	23.8	0.237	
chr01	*ZNF644*	91404178	c.2733C>A	p.D911E	1	0	1	/	/	T(0.46)	N(0.33)	B(0.001)	12.53	0.212	
chr01	*ZNF644*	91404273	c.2638A>G	p.S880G	1	0	1	/	/	T(0.218)	N(-0.36)	B(0)	15.34	0.072	

chr01	*ZNF644*	91404360	c.2551G>C	p.D851H	1	1	0	/	/	D(0.0)	N(-1.45)	P(0.573)	25.1	0.662	
chr01	*ZNF644*	91404408	c.2503A>G	p.K835Q	1	1	0	/	/	D(0.0)	N(-0.95)	P(0.823)	26.1	0.23	
chr01	*ZNF644*	91404438	c.2473G>T	p.D825Y	1	0	1	/	/	D(0.001)	N(-1.18)	B(0.34)	22.9	0.085	
chr01	*ZNF644*	91404576	c.2335A>G	p.S779G	1	1	0	26	9.20354E-05	T(0.097)	N(-0.62)	B(0)	15.21	0.039	
chr01	*ZNF644*	91404576	c.2332_2334del	p.Ser779del	1	0	1	3	1.06195E-05	NA	NA	NA	NA	NA	
chr01	*ZNF644*	91404614	c.2297C>A	p.A766D	1	0	1	/	/	D(0.001)	N(-0.53)	B(0.001)	15.64	0.141	
chr01	*ZNF644*	91404614	c.2294_2296del	p.Glu765del	1	0	1	19	6.72986E-05	NA	NA	NA	NA	NA	
chr01	*ZNF644*	91404630	c.2281T>C	p.F761L	1	1	0	/	/	T(0.55)	N(0.87)	B(0)	17.92	0.236	
chr01	*ZNF644*	91404725	c.2186A>G	p.K729R	1	1	0	/	/	D(0.005)	N(-0.36)	B(0.009)	22.2	0.225	
chr01	*ZNF644*	91404749	c.2162A>G	p.Y721C	4	1	3	11	3.89568E-05	D(0.0)	N(-1.03)	B(0.01)	22.5	0.142	
chr01	*ZNF644*	91404780	c.2131A>G	p.I711V	1	0	1	64	0.000226643	T(0.627)	N(0.2)	B(0)	0.001	0.03	
chr01	*ZNF644*	91404783	c.2128A>G	p.T710A	1	0	1	/	/	T(0.091)	N(0.23)	B(0.002)	2.965	0.031	
chr01	*ZNF644*	91404794	c.2117A>G	p.H706R	1	0	1	/	/	T(0.112)	N(-0.15)	B(0)	5.645	0.06	
chr01	*ZNF644*	91404863	c.2048G>C	p.R683T	1	1	0	38	0.000134702	D(0.034)	N(-0.58)	P(0.558)	24.8	0.681	
chr01	*ZNF644*	91404897	c.2014A>G	p.S672G	5	1	4	1	3.98629E-06	D(0.001)	N(-0.71)	B(0.282)	16.42	0.157	Shi Y, et al.
chr01	*ZNF644*	91404947	c.1964C>G	p.S655C	1	0	1	3	1.19619E-05	D(0.0)	N(-1.59)	D(0.993)	24.7	0.364	
chr01	*ZNF644*	91404953	c.1958A>G	p.K653R	1	0	1	4	1.59537E-05	D(0.001)	N(-0.9)	D(0.98)	24.2	0.191	
chr01	*ZNF644*	91404960	c.1951T>C	p.F651L	1	0	1	/	/	D(0.007)	N(-1.3)	D(0.952)	24.5	0.259	
chr01	*ZNF644*	91404978	c.1933A>G	p.K645E	1	0	1	1	3.18552E-05	D(0.023)	N(-0.67)	B(0.192)	22.6	0.237	
chr01	*ZNF644*	91405108	c.1803A>T	p.L601F	1	0	1	20	7.96134E-05	T(0.13)	D(-2.5)	B(0.004)	16.26	0.184	
chr01	*ZNF644*	91405152	c.1759A>G	p.I587V	1	0	1	7	2.47774E-05	T(0.646)	N(0.2)	B(0)	0.009	0.025	Shi Y, et al.
chr01	*ZNF644*	91405217	c.1694A>G	p.K565R	1	0	1	31	0.00011004	T(0.102)	N(-0.52)	D(0.98)	23.3	0.185	
chr01	*ZNF644*	91405229	c.1682T>C	p.I561T	1	0	1	/	/	T(0.079)	N(-0.74)	B(0)	2.008	0.077	
chr01	*ZNF644*	91405274	c.1637T>C	p.I546T	1	0	1	/	/	T(0.228)	N(0.16)	B(0.005)	17.88	0.133	

chr01	*ZNF644*	91405427	c.1484G>T	p.R495L	1	0	1	/	/	D(0.005)	D(-3.7)	D(0.932)	26.9	0.239	
chr01	*ZNF644*	91405508	c.1403T>C	p.I468T	1	0	1	/	/	T(0.068)	N(-0.08)	D(0.998)	24	0.439	
chr01	*ZNF644*	91405512	c.1399A>G	p.M467V	1	0	1	5	1.9911E-05	T(0.006)	N(-1.8)	D(0.977)	24.5	0.437	Cai XB, et al.
chr01	*ZNF644*	91405754	c.1157C>G	p.T386S	1	0	1	/	/	T(0.259)	N(-0.37)	B(0.001)	17.01	0.296	
chr01	*ZNF644*	91405772	c.1139G>A	p.S380N	1	0	1	/	/	T(0.232)	N(0.01)	B(0.01)	19.25	0.249	
chr01	*ZNF644*	91405782	c.1129G>A	p.V377I	1	0	1	/	/	T(0.189)	N(-0.26)	B(0.001)	21.5	0.298	
chr01	*ZNF644*	91405805	c.1106A>T	p.K369M	1	1	0	/	/	D(0.005)	N(-1.24)	P(0.453)	24.2	0.19	
chr01	*ZNF644*	91405811	c.1100C>T	p.P367L	36	2	34	23	8.13981E-05	D(0.001)	D(-2.62)	B(0.033)	23	0.159	
chr01	*ZNF644*	91405817	c.1094A>G	p.Y365C	1	0	1	1	3.98089E-06	T(0.139)	N(-1.36)	B(0.001)	19.09	0.28	
chr01	*ZNF644*	91405869	c.1042G>C	p.E348Q	1	0	1	/	/	D(0.009)	N(-0.2)	D(0.966)	23.2	0.195	
chr01	*ZNF644*	91405943	c.965_967del	p.Glu322del	4	0	4	2	7.97137E-06	NA	NA	NA	NA	NA	
chr01	*ZNF644*	91405948	c.963A>T	p.Q321H	1	0	1	/	/	D(0.022)	N(0.03)	B(0.002)	14.87	0.073	
chr01	*ZNF644*	91405979	c.932C>G	p.S311C	1	0	1	/	/	D(0.028)	N(-1.41)	P(0.533)	21.8	0.166	
chr01	*ZNF644*	91405998	c.913G>A	p.E305K	83	2	81	202	0.000716058	D(0.002)	N(-1.15)	D(0.097)	26.2	0.23	Liu F, et al.
chr01	*ZNF644*	91406007	c.904C>T	p.R302C	1	0	1	5	1.99461E-05	D(0.002)	N(-1.39)	D(0.988)	28.5	0.356	
chr01	*ZNF644*	91406082	c.829G>A	p.D277N	4	0	4	/	/	D(0.004)	N(-1.21)	B(0.205)	22.9	0.247	
chr01	*ZNF644*	91406351	c.560C>T	p.P187L	6	0	6	10	3.54313E-05	T(0.078)	N(-1.03)	B(0.001)	20.8	0.216	
chr01	*ZNF644*	91406391	c.520G>C	p.V174L	1	0	1	/	/	T(0.158)	N(-0.45)	B(0.002)	21.3	0.229	
chr01	*ZNF644*	91406417	c.494C>G	p.T165R	4	0	4	9	3.18972E-05	D(0.001)	N(-1.56)	B(0.091)	23.3	0.295	
chr01	*ZNF644*	91406495	c.416C>G	p.T139S	1	0	1	/	/	D(0.007)	N(-0.6)	P(0.901)	22.9	0.28	
chr01	*ZNF644*	91406607	c.304A>G	p.T102A	1	0	1	7	2.47884E-05	T(0.551)	N(-0.2)	B(0)	13	0.203	
chr01	*ZNF644*	91406741	c.170A>G	p.H57R	1	0	1	2	8.01462E-06	D(0.027)	N(-0.43)	B(0)	9.452	0.129	
chr01	*ZNF644*	91406799	c.112G>A	p.A38T	1	0	1	1	4.14983E-06	D(0.0)	N(-1.07)	D(0.989)	25.3	0.272	
chr01	*ZNF644*	91447882	c.29A>T	p.N10I	1	0	1	1	4.01745E-06	T(1)	N(0.91)	B(0.274)	22.5	0.047	

chr01	*ZNF644*	91447889	c.22G>T	p.D8Y	1	0	1	/	/	D(0.0)	N(-0.41)	B(0.001)	25.5	0.163	
chr04	*CCDC111*	185578298	c.4A>G	p.N2D	1	0	1	/	/	T(0.422)	N(-0.78)	B(0.016)	19.06	0.055	
chr04	*CCDC111*	185578346	c.52C>G	p.H18D	1	0	1	/	/	T(0.235)	N(-0.94)	B(0.032)	12.99	0.068	
chr04	*CCDC111*	185578374	c.80T>C	p.V27A	1	0	1	/	/	T(0.641)	N(-0.72)	B(0.152)	16.45	0.038	
chr04	*CCDC111*	185578410	c.116C>G	p.P39R	1	0	1	/	/	T(0.064)	N(-0.86)	B(0.277)	22.9	0.109	
chr04	*CCDC111*	185580539	c.226C>T	p.R76C	2	0	2	15	5.96749E-05	D(0.0)	D(-6.64)	D(1.0)	27.2	0.672	
chr04	*CCDC111*	185580578	c.265T>G	p.Y89D	42	1	41	122	0.000431391	D(0.009)	D(-4.19)	P(0.771)	23.6	0.265	Zhao FX, et al.
chr04	*CCDC111*	185580582	c.269A>G	p.Y90C	1	0	1	4	1.41451E-05	D(0.001)	D(-5.65)	D(1.0)	26.3	0.639	
chr04	*CCDC111*	185583016	c.367C>T	p.P123S	5	0	5	7	2.78567E-05	D(0.006)	D(-3.23)	P(0.756)	24.8	0.211	
chr04	*CCDC111*	185583038	c.390dupG	p.V131Gfs*6	137	16	121	276	0.000976887	NA	NA	NA	NA	NA	
chr04	*CCDC111*	185583041	c.392T>G	p.V131G	3	1	2	/	/	D(0.001)	D(-5.48)	D(1.0)	29.5	0.776	
chr04	*CCDC111*	185583044	c.395C>T	p.A132V	1	0	1	/	/	T(0.146)	N(-1.58)	B(0.208)	23.8	0.141	
chr04	*CCDC111*	185587098	c.436T>C	p.Y146H	5	1	4	6	2.43681E-05	D(0.036)	D(-4.31)	B(0.067)	17.06	0.157	
chr04	*CCDC111*	185587099	c.437A>G	p.Y146C	1	0	1	/	/	D(0.002)	D(-7.19)	D(0.916)	23	0.369	
chr04	*CCDC111*	185587140	c.478T>C	p.S160P	1	0	1	/	/	D(0.006)	D(-4.25)	P(0.898)	26.7	0.604	
chr04	*CCDC111*	185587164	c.502C>T	p.Q168*	1	0	1	16	6.39044E-05	NA	NA	NA	39	NA	
chr04	*CCDC111*	185587188	c.526G>A	p.D176N	1	0	1	/	/	T(0.755)	N(0.61)	B(0.002)	16.54	0.055	
chr04	*CCDC111*	185587215	c.553G>A	p.V185I	12	2	10	42	0.000155987	T(0.112)	N(-0.83)	B(0.026)	22.3	0.2	
chr04	*CCDC111*	185593346	c.576T>G	p.I192M	5	1	4			T(0.195)	N(-1.58)	D(0.947)	21	0.239	
chr04	*CCDC111*	185593584	c.814A>G	p.M272V	1	0	1	/	/	T(0.3)	N(-0.37)	B(0.0)	0.001	0.012	
chr04	*CCDC111*	185599423	c.882A>C	p.K294N	1	1	0	2	8.52798E-06	D(0.025)	N(-2.38)	D(0.995)	23.9	0.186	
chr04	*CCDC111*	185599443	c.902G>A	p.R301H	1	0	1	48	0.000176651	T(0.551)	N(-1.00)	T(0.001)	5.586	0.021	
chr04	*CCDC111*	185599473	c.932A>G	p.K311R	2	0	2	3	1.22054E-05	T(0.788)	N(0.02)	B(0.001)	0.001	0.077	
chr04	*CCDC111*	185599478	c.937T>G	p.F313V	1	0	1	2	8.13464E-06	T(0.639)	N(0.64)	B(0.003)	12.98	0.042	

chr04	*CCDC111*	185599544	c.1003G>A	p.V335I	1	0	1	1	4.16438E-06	T(0.126)	N(-0.36)	B(0.032)	16.62	0.033	
chr04	*CCDC111*	185603419	c.1025G>A	p.R342Q	1	0	1	1	4.01287E-06	D(0.033)	N(-2.08)	B(0.028)	23.1	0.155	
chr04	*CCDC111*	185603427	c.1033A>G	p.T345A	1	0	1	/	/	D(0.040)	N(-1.48)	B(0.137)	22.3	0.064	
chr04	*CCDC111*	185606572	c.1106T>C	p.I369T	12	0	12	30	0.000106151	T(0.31)	N(0.17)	B(0.031)	15.95	0.09	
chr04	*CCDC111*	185606598	c.1132C>A	p.P378T	1	0	1	1	3.97962E-06	D(0.009)	D(-6.03)	P(0.863)	25	0.246	
chr04	*CCDC111*	185606611	c.1145A>G	p.H382R	2	0	2	2	7.95957E-06	T(0.553)	N(0.32)	B(0.0)	5.323	0.045	
chr04	*CCDC111*	185606650	c.1184G>C	p.G395A	4	0	4	17	6.01485E-05	T(0.146)	D(-4.14)	D(0.998)	31	0.466	
chr04	*CCDC111*	185606738	c.1195C>T	p.R399C	1	0	1	4	1.41751E-05	D(0.046)	D(-2.73)	B(0.018)	23.9	0.12	
chr04	*CCDC111*	185606739	c.1196G>A	p.R399H	2	0	2	7	2.78951E-05	D(0.029)	N(-1.54)	B(0.018)	19.57	0.133	
chr04	*CCDC111*	185606778	c.1235A>G	p.D412G	1	0	1	/	/	D(0.0)	D(-4.51)	D(0.991)	29.2	0.54	
chr04	*CCDC111*	185606792	c.1249C>T	p.R417W	2	0	2	19	6.73315E-05	D(0.0)	D(-7.73)	D(1.0)	33	0.644	
chr04	*CCDC111*	185606793	c.1250G>A	p.R417Q	1	0	1	10	3.98708E-05	D(0.0)	D(-3.87)	D(0.999)	32	0.646	
chr04	*CCDC111*	185612822	c.1381T>C	p.F461L	1	1	0	2	7.96197E-06	T(0.491)	N(-1.25)	B(0.001)	20.9	0.063	
chr04	*CCDC111*	185615675	c.1431_1433delAGA	p.Glu478del	27	5	22	62	0.000220164	NA	NA	NA	NA	NA	
chr04	*CCDC111*	185615718	c.1468_1471del	p.N490Kfs*33	1	0	1	/	/	NA	NA	NA	NA	NA	
chr04	*CCDC111*	185615843	c.1593_1594insGC	p.E533Lfs*22	1	0	1	/	/	NA	NA	NA	NA	NA	
chr12	*SLC39A5*	56625089	c.31G>A	p.A11T	1	1	0	2	8.11004E-06	T(0.175)	N(-0.34)	B(0.001)	12.6	0.06	
chr12	*SLC39A5*	56625098	c.40T>C	p.C14R	1	0	1	1	4.03672E-06	D(0.004)	N(-1.83)	B(0.102)	17.07	0.17	
chr12	*SLC39A5*	56625099	c.41G>C	p.C14S	1	1	0	5	2.01475E-05	D(0.009)	N(-1.07)	B(0.002)	13.02	0.057	
chr12	*SLC39A5*	56625199	c.141C>G	p.Y47*	4	0	4	6	2.12441E-05	NA	NA	NA	28.4	NA	Guo H, et al.
chr12	*SLC39A5*	56625237	c.179G>A	p.R60Q	1	0	1	58	0.000205894	D(0)	N(-2.23)	P(0.637)	25	0.069	
chr12	*SLC39A5*	56625260	c.202G>C	p.G68R	1	0	1	/	/	D(0)	D(-6)	D(0.999)	27.3	0.393	
chr12	*SLC39A5*	56625270	c.212A>G	p.Q71R	2	0	2	1	4.03584E-06	T(0.325)	N(-0.47)	P(0.489)	24.3	0.082	
chr12	*SLC39A5*	56625308	c.250C>T	p.R84W	2	0	2	28	0.000124739	D(0.001)	N(-1.97)	P(0.635)	23.8	0.107	Cai XB, et al.

chr12	*SLC39A5*	56625309	c.251G>A	p.R84Q	2	0	2	19	7.41475E-05	T(0.06)	N(-1.24)	B(0.008)	5.113	0.016	
chr12	*SLC39A5*	56626510	c.325G>T	p.G109W	17	1	16	30	0.000107374	D(0)	D(-3.23)	P(0.868)	25.3	0.167	
chr12	*SLC39A5*	56626574	c.389G>A	p.R130H	16	0	16	45	0.000160594	T(0.586)	N(0.82)	B(0.0)	5.244	0.017	
chr12	*SLC39A5*	56626589	c.404C>T	p.S135L	10	0	10	23	8.18867E-05	D(0.007)	N(-1.75)	P(0.576)	26.7	0.051	
chr12	*SLC39A5*	56626624	c.439G>C	p.D147H	1	0	1	/	/	T(0.089)	N(-1.85)	D(0.997)	25	0.17	
chr12	*SLC39A5*	56626628	c.443A>C	p.H148P	1	0	1	/	/	D(0)	D(-5.5)	D(0.991)	25.7	0.147	
chr12	*SLC39A5*	56626657	c.471+1G>T	splicing	2	0	2	/	/	NA	NA	NA	33	NA	
chr12	*SLC39A5*	56628660	c.524C>G	p.P175R	1	0	1	/	/	T(0.248)	N(-2.05)	B(0.174)	20.9	0.212	
chr12	*SLC39A5*	56628672	c.536G>C	p.R179P	1	0	1	/	/	T(0.244)	N(-1.01)	B(0.071)	10.68	0.159	
chr12	*SLC39A5*	56628690	c.554G>A	p.C185Y	1	0	2	5	1.76921E-05	D(0)	D(-9.64)	D(0.997)	27.4	0.841	
chr12	*SLC39A5*	56628712	c.576C>G	p.I192M	1	0	1	/	/	D(0)	N(-1.88)	P(0.682)	14.93	0.489	
chr12	*SLC39A5*	56628713	c.577G>A	p.D193N	7	0	7	24	8.49642E-05	D(0.002)	D(-3.91)	P(0.533)	25.8	0.382	
chr12	*SLC39A5*	56628722	c.586G>A	p.V196I	1	0	1	18	7.16994E-05	T(0.116)	N(-0.66)	P(0.887)	23.5	0.237	
chr12	*SLC39A5*	56628738	c.602C>T	p.P201L	1	0	1	18	6.37945E-05	T(0.763)	N(-0.83)	B(0.002)	12.47	0.052	
chr12	*SLC39A5*	56628954	c.648T>G	p.S216R	2	0	2	1	5.07882E-06	D(0.003)	D(-2.65)	B(0.158)	17.77	0.322	
chr12	*SLC39A5*	56629010	c.704G>A	p.R235Q	2	0	2	10	3.90183E-05	D(0.023)	N(0.21)	D(0.914)	23.7	0.261	
chr12	*SLC39A5*	56629025	c.719G>A	p.R240H	1	0	1	3	1.33407E-05	T(0.192)	N(-0.35)	B(0.003)	11.51	0.055	
chr12	*SLC39A5*	56629033	c.727C>T	p.R243W	1	0	1	15	5.94036E-05	D(0.001)	D(-3.65)	P(0.88)	25.5	0.302	
chr12	*SLC39A5*	56629055	c.749G>A	p.G250E	1	1	0	/	/	T(0.082)	N(-0.38)	D(0.958)	24.1	0.236	
chr12	*SLC39A5*	56629064	c.758C>A	p.A253E	1	0	1	3	1.54316E-05	D(0)	D(-4.68)	D(1.0)	32	0.821	
chr12	*SLC39A5*	56629362	c.823G>A	p.A275T	7	1	6	23	9.15744E-05	T(0.57)	N(-0.17)	B(0.002)	0.002	0.014	
chr12	*SLC39A5*	56629374	c.835G>A	p.G279R	1	0	1	43	0.000152145	T(0.423)	N(-0.48)	P(0.78)	18.66	0.2	
chr12	*SLC39A5*	56629394	c.858_904dupCCCG(	p.E302Afs*117	1	0	1	3	1.19402E-05	NA	NA	NA	NA	NA	
chr12	*SLC39A5*	56629399	c.860C>T	p.P287L	22	0	22	57	0.000201713	D(0.047)	N(-0.57)	B(0.034)	20.3	0.191	Feng CY, et al.

chr12	*SLC39A5*	56629414	c.875T>C	p.L292P	2	0	2	3	1.19384E-05	D(0)	D(-5.11)	D(0.999)	24.6	0.55	
chr12	*SLC39A5*	56629416	c.877G>A	p.G293R	1	0	1	3	1.19384E-05	D(0.001)	D(-3.83)	D(0.993)	25.5	0.408	Wang L, et al.
chr12	*SLC39A5*	56629417	c.878G>C	p.G293A	1	0	1	64	0.000226374	T(0.086)	N(-1.46)	P(0.736)	24	0.12	
chr12	*SLC39A5*	56629471	c.932G>A	p.R311Q	1	0	1	16	5.66436E-05	T(0.249)	N(-0.49)	B(0.006)	16.69	0.034	
chr12	*SLC39A5*	56629474	c.935G>C	p.G312A	1	0	1	/	/	D(0.017)	N(-0.62)	P(0.668)	23.3	0.119	
chr12	*SLC39A5*	56629483	c.944C>A	p.P315Q	1	0	1	/	/	T(0.445)	N(0.81)	P(0.701)	24.2	0.136	
chr12	*SLC39A5*	56630192	c.958C>T	p.R320*	2	0	2	3	1.19307E-05	NA	NA	NA	36	NA	
chr12	*SLC39A5*	56630198	c.964C>T	p.R322*	5	0	5	128	0.000452505	NA	NA	NA	33	NA	
chr12	*SLC39A5*	56630199	c.965G>A	p.R322Q	1	0	1	2	7.95349E-06	T(0.403)	N(0.5)	P(0.763)	18.25	0.116	
chr12	*SLC39A5*	56630203	c.971dupA	p.N324Lfs*59	2	0	1	2	7.95323E-06	NA	NA	NA	NA	NA	
chr12	*SLC39A5*	56630216	c.982C>T	p.R328C	1	0	1	24	8.48404E-05	D(0.031)	N(-0.7)	B(0.376)	16.62	0.099	
chr12	*SLC39A5*	56630217	c.983G>A	p.R328H	1	0	1	70	0.000247453	T(0.289)	N(1.97)	B(0.286)	14.23	0.14	
chr12	*SLC39A5*	56630229	c.995C>T	p.P332L	1	0	1	4	1.59065E-05	T(0.346)	N(-0.41)	B(0.011)	17.77	0.03	
chr12	*SLC39A5*	56630255	c.1022dupA	p.Q341fs	1	0	1	/	/	NA	NA	NA	NA	NA	Zheng YH, et al.
chr12	*SLC39A5*	56630271	c.1037C>T	p.A346V	1	0	1	32	0.000113137	T(0.149)	N(-0.27)	B(0.003)	20.8	0.032	
chr12	*SLC39A5*	56630368	c.1045C>A	p.P349T	2	0	2	1	3.1837E-05	T(0.388)	N(0.57)	B(0.022)	15.2	0.056	
chr12	*SLC39A5*	56630369	c.1046C>A	p.P349Q	3	1	2	5	1.77486E-05	T(0.329)	N(-0.01)	P(0.712)	17.14	0.11	
chr12	*SLC39A5*	56630377	c.1054C>T	p.Q352*	1	0	1	/	/	NA	NA	NA	38	NA	
chr12	*SLC39A5*	56630389	c.1071_1073delGAA	p.K357del	1	0	1	24	8.51619E-05	NA	NA	NA	NA	NA	
chr12	*SLC39A5*	56630394	c.1071G>T	p.K357N	1	0	1	1	3.99278E-06	T(0.214)	N(-0.07)	B(0.016)	13.2	0.036	
chr12	*SLC39A5*	56630404	c.1081C>T	p.H361Y	1	0	1	/	/	T(0.09)	N(-0.81)	B(0.194)	0.188	0.163	
chr12	*SLC39A5*	56630474	c.1151C>T	p.T384M	1	0	1	10	3.55023E-05	D(0.003)	N(-1.25)	P(0.636)	23.1	0.203	
chr12	*SLC39A5*	56630481	c.1158G>T	p.M386I	1	0	1	1	4.01313E-06	D(0.004)	D(-3.65)	D(0.992)	26.8	0.427	
chr12	*SLC39A5*	56630527	c.1204A>G	p.I402V	3	0	3	1	4.19794E-06	D(0.007)	N(-0.65)	P(0.506)	24.2	0.136	

chr12	*SLC39A5*	56630729	c.1222G>A	p.D408N	1	1	0	/	/	T(0.069)	N(-0.26)	P(0.673)	24.1	0.095	
chr12	*SLC39A5*	56630744	c.1237G>A	p.G413S	1	0	1	5	1.76967E-05	D(0)	D(-5.77)	D(1.0)	28.6	0.73	
chr12	*SLC39A5*	56630745	c.1238G>C	p.G413A	1	1	0	/	/	D(0)	D(-5.77)	D(1.0)	27.5	0.904	
chr12	*SLC39A5*	56630975	c.1330C>T	p.R444W	5	0	5	7	2.80359E-05	D(0.018)	D(-4.81)	P(0.828)	22.4	0.193	
chr12	*SLC39A5*	56631093	c.1448G>A	p.G483E	1	0	1	5	2.04705E-05	D(0)	D(-7.69)	B(0.311)	23.7	0.937	
chr12	*SLC39A5*	56631110	c.1465G>A	p.A489T	1	0	1	/	/	D(0)	D(-3.11)	D(0.96)	28.2	0.426	
chr12	*SLC39A5*	56631388	c.1496G>A	p.R499H	1	1	0	24	9.56244E-05	T(1)	N(2.55)	B(0.061)	20.5	0.075	
chr12	*SLC39A5*	56631394	c.1502C>T	p.P501L	1	1	0	274	0.000970633	D(0.035)	N(-1.68)	P(0.888)	24.1	0.135	
chr12	*SLC39A5*	56631409	c.1517C>T	p.T506M	166	9	157	210	0.000744713	T(0.256)	N(-0.01)	B(0.024)	0.681	0.005	
chr12	*SLC39A5*	56631486	c.1594C>T	p.R532W	1	0	1	2	8.96049E-06	D(0.002)	D(-3.4)	B(0.23)	24.9	0.185	
chr12	*SLC39A5*	56631487	c.1595G>A	p.R532Q	2	0	2	11	4.30616E-05	T(0.54)	N(0.9)	B(0.0)	12.93	0.03	
chr05	*P4HA2*	131531143	c.1402G>A	p.V468I	1	1	0	20	7.07774E-05	D(0.002)	N(-0.97)	D(0.984)	28.7	0.558	
chr05	*P4HA2*	131534013	c.1365+1G>C	splicing	2	1	1	7	2.78392E-05	NA	NA	NA	27.6	NA	
chr05	*P4HA2*	131533917	c.1352_1353del	p.V451Gfs*9	4	0	4	3	1.19316E-05	NA	NA	NA	NA	NA	Guo H, et al.
chr05	*P4HA2*	131533921	c.1349G>C	p.R450P	1	0	1	3	1.19312E-05	D(0.000)	N(-6.55)	D(1.000)	31	0.851	
chr05	*P4HA2*	131533955	c.1315C>T	p.R439*	1	0	1	/	/	NA	NA	NA	47	NA	
chr05	*P4HA2*	131539490	c.1202G>A	p.R401Q	1	0	1	4	1.41456E-05	D(0.001)	D(-3.77)	D(0.975)	33	0.646	
chr05	*P4HA2*	131539788	c.1138C>T	p.R380W	3	0	3	3	1.19297E-05	D(0.000)	D(-7.77)	D(1.000)	24.3	0.654	
chr05	*P4HA2*	131539826	c.1100G>A	p.R367H	2	1	1	2	7.07014E-06	T(0.649)	N(0.02)	B(0.009)	23.5	0.139	
chr05	*P4HA2*	131543448	c.1033A>G	p.M345V	1	0	1	1	3.98035E-06	T(0.117)	N(0.15)	B(0.002)	20.2	0.234	
chr05	*P4HA2*	131543457	c.1024T>G	p.Y342D	3	1	2	2	7.96052E-06	D(0.005)	D(-3.29)	B(0.171)	25.6	0.294	
chr05	*P4HA2*	131543489	c.992A>G	p.D331G	1	0	1	/	/	D(0.033)	D(-4.16)	P(0.781)	31	0.575	
chr05	*P4HA2*	131543519	c.962A>G	p.Q321R	1	1	0	/	/	T(0.636)	N(0.27)	B(0.0)	22	0.146	
chr05	*P4HA2*	131543538	c.943C>G	p.H315D	3	0	3	1	4.1953E-06	T(1.0)	N(1.95)	B(0.0)	16.4	0.239	

chr05	*P4HA2*	131543571	c.910C>T	p.R304C	10	0	10	32	0.000134767	D(0.001)	D(-4.65)	P(0.556)	26.6	0.299	
chr05	*P4HA2*	131544847	c.887G>A	p.G296E	1	0	1	/	/	D(0.002)	D(-6.75)	D(0.997)	31	0.578	
chr05	*P4HA2*	131544851	c.883C>T	p.R295C	1	0	1	8	2.83358E-05	D(0.000)	D(-7.83)	D(0.985)	32	0.472	
chr05	*P4HA2*	131544863	c.871G>A	p.E291K	9	2	7	14	5.57387E-05	D(0.001)	D(-3.8)	D(0.984)	32	0.824	Guo H, et al.
chr05	*P4HA2*	131544998	c.736A>T	p.N246Y	1	0	1	1	3.98191E-06	D(0.000)	D(-7.56)	D(0.996)	29.5	0.728	
chr05	*P4HA2*	131545007	c.727G>A	p.A243T	2	0	2	/	/	D(0.001)	D(-3.61)	P(0.806)	29.9	0.473	
chr05	*P4HA2*	131545013	c.721G>A	p.E241K	1	0	1	1	3.98661E-06	T(0.063)	N(-1.75)	B(0.069)	23.6	0.147	
chr05	*P4HA2*	131545988	c.698T>G	p.L233R	1	0	1	/	/	D(0.000)	D(-5.74)	D(0.924)	26.1	0.779	
chr05	*P4HA2*	131545994	c.692delG	p.R231Pfs*32	1	0	1	/	/	NA	NA	NA	NA	NA	
chr05	*P4HA2*	131546009	c.677C>T	p.A226V	1	0	1	1	3.98057E-06	D(0.003)	D(-3.62)	D(0.912)	23.9	0.607	
chr05	*P4HA2*	131546060	c.626T>G	p.V209G	1	0	1	/	/	D(0.000)	D(-6.37)	B(0.342)	23.8	0.362	
chr05	*P4HA2*	131546132	c.554C>T	p.T185M	1	0	1	6	2.12116E-05	D(0.000)	D(-5.2)	D(0.931)	26.8	0.571	
chr05	*P4HA2*	131549659	c.419A>G	p.Q140R	8	0	8	17	6.01047E-05	D(0.001)	D(-3.88)	D(0.993)	27	0.934	Guo H, et al.
chr05	*P4HA2*	131549683	c.395C>A	p.A132D	1	0	1	/	/	D(0.000)	D(-5.69)	D(0.998)	29	0.786	
chr05	*P4HA2*	131549719	c.359G>A	p.R120Q	3	0	3	3	1.19357E-05	D(0.029)	D(-2.94)	P(0.844)	31	0.331	
chr05	*P4HA2*	131552925	c.296C>T	p.A99V	1	0	1	6	2.38698E-05	T(0.11)	N(-0.83)	B(0.105)	22.5	0.15	
chr19	*BSG*	572668	c.34G>A	p.A12T	1	1	0	/	/	T(0.65)	N(-0.44)	B(0.001)	9.58	0.104	
chr19	*BSG*	577776	c.70G>A	p.G24S	24	2	22	9	0.000101338	D(0)	D(-3.45)	D(0.996)	23.9	0.324	
chr19	*BSG*	577792	c.86C>T	p.P29L	1	0	1	6	8.52418E-05	D(0.001)	D(-6.11)	D(1)	23.3	0.301	
chr19	*BSG*	577845	c.139G>T	p.V47L	4	0	4	2	1.21886E-05	D(0.006)	N(-1.41)	B(0.243)	17.73	0.044	
chr19	*BSG*	577855	c.149C>T	p.P50L	1	0	1	/	/	D(0)	D(-5.98)	D(1)	23.9	0.666	
chr19	*BSG*	577863	c.157G>A	p.E53K	1	0	1	10	4.35297E-05	D(0)	N(-2.38)	D(0.979)	25	0.37	
chr19	*BSG*	577869	c.163C>T	p.Q55*	1	0	1	/	/	NA	NA	NA	43	NA	
chr19	*BSG*	577911	c.205C>T	p.Q69*	2	0	2	/	/	NA	NA	NA	42	NA	Jin ZB, et al.

chr19	*BSG*	577947	c.241A>G	p.I81V	2	0	2	/	/	T(0.29)	N(-0.48)	D(0.992)	23	0.192	
chr19	*BSG*	578022	c.316T>C	p.Y106H	1	0	1	/	/	D(0.002)	D(-3.21)	D(1)	23.3	0.425	
chr19	*BSG*	578031	c.325C>T	p.R109W	1	0	1	27	9.78374E-05	D(0.001)	D(-4.75)	D(1)	26	0.537	
chr19	*BSG*	578047	c.341C>T	p.P114L	1	0	1	2	8.24076E-06	D(0)	D(-6.44)	D(1)	25	0.269	
chr19	*BSG*	578091	c.385C>T	p.R129C	4	1	3	4	1.76252E-05	D(0.001)	D(-4.63)	D(0.983)	25.7	0.421	
chr19	*BSG*	578104	c.398T>A	p.V133D	3	0	3	1	4.63302E-06	D(0.012)	N(-0.79)	B(0.087)	17.45	0.139	
chr19	*BSG*	578122	c.415+1G>A	splicing	1	1	0	7	3.30613E-05	/	/	/	29	/	Jin ZB, et al.
chr19	*BSG*	579502	c.418G>C	p.G140R	1	0	1	5	2.01055E-05	T(0.066)	D(-2.71)	P(0.806)	15.26	0.056	
chr19	*BSG*	579557	c.473C>T	p.S158F	2	0	2	2	8.00839E-06	T(0.082)	N(-2.24)	B(0.046)	8.711	0.055	
chr19	*BSG*	579571	c.487G>A	p.A163T	1	0	1	5	1.77895E-05	T(0.305)	N(-0.48)	B(0.004)	0.001	0.018	
chr19	*BSG*	579637	c.553G>A	p.G185S	2	1	1	12	4.35202E-05	T(1.0)	N(0.35)	B(0.067)	8.635	0.087	
chr19	*BSG*	579647	c.563C>T	p.T188M	3	0	3	9	3.28194E-05	T(0.555)	N(-1.56)	P(0.856)	11.08	0.081	
chr19	*BSG*	580389	c.583G>C	p.D195H	140	11	129	178	0.000637381	T(0.111)	N(-1.87)	B(0.105)	14.98	0.077	
chr19	*BSG*	580399	c.593G>T	p.W198L	1	0	1	/	/	T(0.199)	N(-1.07)	B(0.023)	9.234	0.068	
chr19	*BSG*	580428	c.622G>A	p.E208K	2	0	2	15	5.36765E-05	T(0.279)	N(-2.06)	B(0.224)	14.56	0.156	
chr19	*BSG*	580436	c.630G>C	p.M210I	1	0	1	/	/	T(0.184)	N(-0.19)	B(0.0)	1.816	0.034	
chr19	*BSG*	580684	c.694A>C	p.I232L	1	0	1	/	/	T(0.785)	N(-0.23)	B(0.038)	12.38	0.114	
chr19	*BSG*	580700	c.710C>T	p.T237M	2	0	2	3	1.06557E-05	T(0.084)	D(-2.72)	P(0.86)	17.58	0.206	
chr19	*BSG*	580730	c.740T>A	p.V247E	1	0	1	2	7.99603E-06	D(0.007)	N(-1.8)	B(0.102)	12.86	0.102	
chr19	*BSG*	580757	c.767A>C	p.Y256S	1	0	1	/	/	T(0.133)	D(-6.56)	P(0.863)	12.99	0.148	
chr19	*BSG*	580762	c.772A>T	p.I258F	1	0	1	/	/	T(0.078)	N(-1.82)	B(0.279)	0.516	0.282	
chr19	*BSG*	580765	c.775A>G	p.T259A	1	0	1	/	/	T(0.701)	N(-1.01)	B(0.001)	0.061	0.084	
chr19	*BSG*	580766	c.776C>T	p.T259I	2	0	2	/	/	T(0.182)	N(-2.04)	B(0.046)	0.018	0.156	
chr19	*BSG*	580771	c.781T>A	p.S261T	1	0	1	/	/	T(0.269)	N(-1.44)	B(0.001)	0.098	0.167	

chr19	*BSG*	580774	c.784G>A	p.E262K	1	0	1	/	/	T(0.776)	N(-0.93)	B(0.03)	9.462	0.069
chr19	*BSG*	580778	c.788A>G	p.D263G	1	0	1	/	/	T(0.414)	N(-2.17)	B(0.105)	9.424	0.115
chr19	*BSG*	580780	c.790A>C	p.K264Q	1	0	1	/	/	T(0.958)	N(1.12)	B(0.0)	0.249	0.086
chr19	*BSG*	580781	c.791A>G	p.K264R	2	0	2	/	/	T(0.516)	N(-0.24)	B(0.012)	21.9	0.235
chr19	*BSG*	580782	c.792G>T	p.K264N	1	0	1	/	/	T(0.38)	N(-1.1)	B(0.003)	27.1	0.137
chr19	*BSG*	580783	c.792+1G>T	splicing	1	0	1	/	/	NA	NA	NA	33	NA
chr19	*BSG*	581333	c.811G>A	p.E271K	4	0	4	1	4.02557E-06	T(0.163)	N(-1.45)	B(0.009)	9.808	0.172
chr19	*BSG*	581369	c.847T>A	p.S283T	1	0	1	/	/	T(1.0)	N(0.97)	B(0.017)	1.203	0.115
chr19	*BSG*	581405	c.883G>A	p.D295N	1	0	1	/	/	D(0.001)	D(-4.78)	D(0.999)	25.6	0.78
chr19	*BSG*	581418	c.896A>G	p.Y299C	1	0	1	/	/	D(0.0)	D(-8.6)	D(1.0)	23.3	0.762
chr19	*BSG*	581495	c.973C>T	p.L325F	1	0	1	/	/	D(0.029)	D(-3.53)	P(0.669)	24.8	0.194
chr19	*BSG*	581516	c.994G>A	p.V332M	2	0	2	/	/	D(0.005)	D(-2.92)	D(0.992)	29.4	0.287
chr19	*BSG*	582308	c.1072_1074del	p.358_358del	1	0	1	/	/	NA	NA	NA	NA	NA
chr19	*BSG*	582317	c.1081G>A	p.G361S	1	0	1	5	1.87418E-05	D(0.017)	D(-5.26)	P(0.676)	25	0.179
chr19	*BSG*	582535	c.1116G>C	p.Q372H	1	0	1	/	/	NA	NA	NA	18.51	0.037
chr19	*BSG*	582539	c.1120G>A	p.D374N	1	0	1	/	/	T(0.181)	N(-0.56)	B(0.232)	23.5	0.103
chr13	*DZIP1*	96237112	c.2402T>G	p.I801R	1	0	1	/	/	T(0.518)	N(0.91)	B(0.002)	7.901	0.023
chr13	*DZIP1*	96237143	c.2314G>T	p.D772Y	1	0	1	/	/	D(0.001)	D(-6.2)	D(0.993)	26	0.332
chr13	*DZIP1*	96237152	c.2362G>A	p.E788K	1	0	1	/	/	D(0.007)	N(-2.43)	B(0.155)	23	0.055
chr13	*DZIP1*	96237163	c.2351G>T	p.C784F	1	0	1	3	1.22498E-05	T(0.696)	D(-2.6)	B(0.003)	14.43	0.081
chr13	*DZIP1*	96238267	c.2342A>C	p.E781A	1	1	0	1	4.21596E-06	T(0.171)	D(-2.68)	P(0.525)	24.4	0.068
chr13	*DZIP1*	96238373	c.2236C>T	p.P746S	1	1	0	6	2.43942E-05	D(0.014)	N(-0.11)	B(0.044)	13.43	0.033
chr13	*DZIP1*	96238382	c.2227G>A	p.V743I	1	0	1	7	2.55437E-05	T(0.832)	N(0.0)	B(0.002)	0.14	0.023
chr13	*DZIP1*	96238385	c.2224G>A	p.A742T	1	0	1	7	2.898E-05	T(0.776)	N(0.21)	B(0.005)	1.731	0.004

chr13	*DZIP1*	96239858	c.2153A>G	p.K718R	1	0	1	5	1.9885E-05	T(0.37)	N(-1.06)	B(0.036)	12.62	0.069	
chr13	*DZIP1*	96239873	c.2138G>C	p.G713A	67	3	64	85	0.000300525	T(0.318)	N(-1.39)	B(0.03)	0.076	0.023	Lee JK, et al.
chr13	*DZIP1*	96239900	c.2111C>T	p.P704L	1	0	1	4	1.59084E-05	T(0.235)	N(-1.25)	B(0.006)	3.427	0.01	
chr13	*DZIP1*	96239909	c.2102T>A	p.L701H	1	0	1	/	/	D(0.005)	N(-2.28)	D(0.934)	15.81	0.099	
chr13	*DZIP1*	96239940	c.2070_2071insGAC	p.L691delinsDL	2	0	2	/	/	NA	NA	NA	NA	NA	
chr13	*DZIP1*	96241449	c.1983_1985del	p.K661del	3	1	2	4	1.45369E-05	NA	NA	NA	NA	NA	
chr13	*DZIP1*	96242562	c.1814A>C	p.K605T	4	0	4	9	3.18857E-05	D(0.005)	D(-3.49)	B(0.107)	19.57	0.096	
chr13	*DZIP1*	96242599	c.1720C>T	p.Q574*	1	0	1	/	/	NA	NA	NA	NA	NA	
chr13	*DZIP1*	96242685	c.1634G>A	p.G545D	1	0	1	25	9.28436E-05	D(0.0)	D(-5.29)	P(0.787)	23.4	0.166	
chr13	*DZIP1*	96242689	c.1687C>T	p.R563C	1	0	1	5	2.09865E-05	D(0.002)	D(-4.02)	P(0.681)	23.4	0.249	
chr13	*DZIP1*	96251636	c.1520T>G	p.L507R	31	2	29	59	0.000208674	T(0.082)	D(-2.53)	B(0.221)	23	0.117	
chr13	*DZIP1*	96251642	c.1512_1514del	p.E506del	1	0	1	/	/	NA	NA	NA	NA	NA	
chr13	*DZIP1*	96251645	c.1511T>C	p.I504T	1	1	0	5	1.98923E-05	T(0.3)	N(-1.66)	B(0.018)	27.4	0.122	
chr13	*DZIP1*	96251663	c.1493C>T	p.S498L	1	0	1	2	7.95855E-06	T(0.125)	N(-1.34)	B(0.011)	23.5	0.032	
chr13	*DZIP1*	96251672	c.1484C>G	p.A495G	1	1	0	/	/	T(0.188)	N(-1.19)	B(0.015)	21.8	0.024	
chr13	*DZIP1*	96258266	c.1468A>G	p.M490V	1	0	1	1	3.97763E-06	T(0.694)	N(-0.51)	B(0.0)	0.006	0.043	
chr13	*DZIP1*	96261723	c.1408C>T	p.P470S	1	0	1	/	/	D(0.036)	N(-0.73)	B(0.116)	15.74	0.048	
chr13	*DZIP1*	96263705	c.1357C>T	p.P453S	1	0	1	/	/	T(0.424)	N(-1.61)	B(0.001)	10.95	0.037	
chr13	*DZIP1*	96264395	c.1210A>G	p.I404V	3	1	2	/	/	T(0.637)	N(-0.17)	B(0.078)	16.69	0.065	
chr13	*DZIP1*	96264398	c.1207A>G	p.M403V	1	0	1	1	4.02998E-06	T(0.504)	N(-1.2)	B(0.0)	12.18	0.042	
chr13	*DZIP1*	96272137	c.1173+2T>A	splicing	2	0	2	6	2.12355E-05	NA	NA	NA	33	NA	
chr13	*DZIP1*	96272141	c.1171C>T	p.R391W	1	1	0	5	1.99102E-05	D(0.018)	D(-2.62)	P(0.804)	32	0.151	
chr13	*DZIP1*	96274615	c.1092G>A	p.M364I	1	1	0	/	/	T(0.335)	N(-1.24)	B(0.001)	18.24	0.058	
chr13	*DZIP1*	96274646	c.1061C>T	p.S354F	1	0	1	/	/	T(0.074)	N(-1.3)	B(0.103)	20.3	0.038	

chr13	*DZIP1*	96274665	c.1042G>A	p.E348K	7	4	3	14	4.95137E-05	D(0.018)	N(-2.1)	P(0.831)	26.2	0.209
chr13	*DZIP1*	96274670	c.1037C>T	p.A346V	2	0	2	4	1.59146E-05	T(0.708)	N(-0.99)	B(0.022)	16.15	0.026
chr13	*DZIP1*	96274702	c.1005G>C	p.Q335H	5	0	5	13	4.59708E-05	D(0.014)	D(-2.64)	B(0.3)	20.2	0.073
chr13	*DZIP1*	96274706	c.1001T>C	p.M334T	1	0	1	/	/	T(0.278)	N(-1.21)	B(0.288)	21.8	0.118
chr13	*DZIP1*	96277044	c.950C>T	p.S317L	6	1	5	47	0.000174648	D(0.036)	D(-3.75)	B(0.217)	24	0.071
chr13	*DZIP1*	96277076	c.916_918del	p.306_306del	1	0	1	/	/	NA	NA	NA	NA	NA
chr13	*DZIP1*	96277078	c.916G>A	p.E306K	1	1	0	1	4.04855E-06	T(0.329)	N(-1.34)	B(0.272)	24.3	0.077
chr13	*DZIP1*	96277130	c.864G>T	p.R288S	1	0	1	1	4.01101E-06	T(0.262)	D(-2.84)	B(0.044)	13.54	0.063
chr13	*DZIP1*	96277151	c.843C>G	p.D281E	2	1	1	6	2.15558E-05	T(1.0)	N(0.62)	B(0.004)	9.541	0.034
chr13	*DZIP1*	96282260	c.793G>A	p.A265T	1	0	1	6	2.15558E-05	T(0.337)	N(-0.51)	B(0.239)	13.75	0.061
chr13	*DZIP1*	96282302	c.751C>A	p.Q251K	1	1	0	1	3.98067E-06	T(0.452)	N(-0.74)	D(0.997)	23.8	0.154
chr13	*DZIP1*	96282320	c.733G>C	p.V245L	2	0	2	3	1.19464E-05	T(0.383)	N(-0.26)	B(0.022)	11.55	0.031
chr13	*DZIP1*	96282334	c.719G>A	p.R240Q	1	1	0	/	/	T(1.0)	N(1.53)	B(0.001)	14.77	0.077
chr13	*DZIP1*	96282335	c.718C>T	p.R240W	1	0	1	7	2.79272E-05	D(0.016)	D(-2.97)	B(0.003)	23.9	0.13
chr13	*DZIP1*	96293581	c.565C>A	p.L189M	1	0	1	/	/	T(0.324)	N(-0.61)	B(0.074)	13.78	0.011
chr13	*DZIP1*	96293593	c.553T>C	p.S185P	1	0	1	/	/	D(0.011)	D(-3.0)	P(0.806)	20.8	0.093
chr13	*DZIP1*	96293658	c.488T>C	p.L163P	2	0	2	/	/	D(0.001)	D(-5.09)	D(0.962)	20.7	0.189
chr13	*DZIP1*	96293724	c.422A>G	p.Q141R	1	0	1	1	3.98565E-06	T(0.053)	N(-1.97)	D(0.989)	22.4	0.14
chr13	*DZIP1*	96293760	c.386A>G	p.E129G	1	0	1	3	1.0629E-05	D(0.0)	D(-6.44)	D(0.999)	27.5	0.634
chr13	*DZIP1*	96293764	c.382A>G	p.I128V	1	0	1	/	/	T(0.196)	N(-0.56)	P(0.644)	22.1	0.1
chr13	*DZIP1*	96293822	c.324C>A	p.H108Q	1	0	1	/	/	D(0.021)	D(-3.77)	D(0.993)	20.3	0.186
chr13	*DZIP1*	96293953	c.193G>A	p.E65K	1	0	1	/	/	D(0.005)	D(-3.41)	D(0.999)	25.7	0.347
chr13	*DZIP1*	96293958	c.188G>A	p.R63Q	1	0	1	/	/	D(0.0)	D(-3.68)	D(0.999)	25.8	0.408
chr13	*DZIP1*	96294052	c.94G>T	p.A32S	1	0	1	/	/	T(1.0)	N(0.26)	B(0.001)	0.001	0.033

chr13	*DZIP1*	96294096	c.50A>T	p.H17L	1	0	1	3	9.74089E-05	D(0.003)	D(-3.23)	P(0.466)	22.9	0.074
chr16	*XYLT1*	17202647	c.2785G>T	p.V929F	1	0	1	/	/	D(0.045)	N(-1.87)	D(0.972)	23.5	0.207
chr16	*XYLT1*	17202649	c.2783C>T	p.P928L	1	0	1	6	2.14359E-05	T(0.117)	D(-5.65)	D(0.934)	22.9	0.345
chr16	*XYLT1*	17202693	c.2739G>A	p.M913I	9	0	9	7	2.48777E-05	T(0.1)	N(-0.5)	B(0.001)	16.42	0.055
chr16	*XYLT1*	17202704	c.2728delG	p.V910Wfs*74	1	0	1	/	/	NA	NA	NA	NA	
chr16	*XYLT1*	17202733	c.2699C>T	p.T900I	1	0	1	1	3.99632E-06	T(0.151)	N(-1.53)	B(0.035)	10.44	0.039
chr16	*XYLT1*	17202742	c.2690C>T	p.S897F	5	2	3	6	2.39754E-05	T(0.297)	N(-0.03)	B(0.002)	12.98	0.055
chr16	*XYLT1*	17202743	c.2689T>C	p.S897P	1	0	1	/	/	T(0.114)	N(-1.45)	B(0.169)	12.98	0.072
chr16	*XYLT1*	17202835	c.2597C>T	p.A866V	1	0	1	/	/	T(0.249)	N(-1.01)	P(0.585)	23.3	0.141
chr16	*XYLT1*	17202842	c.2590C>T	p.R864C	1	0	1	6	2.78458E-05	D(0.032)	D(-3.16)	P(0.855)	25.4	0.193
chr16	*XYLT1*	17202853	c.2579A>G	p.N860S	1	0	1	1	4.97676E-06	T(0.331)	N(-0.24)	B(0.007)	7.411	0.138
chr16	*XYLT1*	17202871	c.2561A>C	p.E854A	22	0	22	67	0.000312599	T(0.149)	D(-3.08)	B(0.256)	24.2	0.129
chr16	*XYLT1*	17211608	c.2452C>T	p.P818S	1	0	1	/	/	D(0.007)	D(-7.32)	D(0.999)	27.7	0.643
chr16	*XYLT1*	17211656	c.2404A>G	p.T802A	1	0	1	/	/	T(0.141)	N(-0.93)	B(0.073)	22.2	0.127
chr16	*XYLT1*	17211674	c.2386G>A	p.D796N	1	0	1	1	3.97636E-06	D(0.047)	D(-4.05)	D(0.998)	29.2	0.363
chr16	*XYLT1*	17211682	c.2378C>T	p.A793V	2	0	2	4	1.59057E-05	D(0.031)	N(-2.43)	D(1)	28.3	0.541
chr16	*XYLT1*	17211686	c.2374G>A	p.A792T	1	0	1	4	1.59056E-05	T(0.068)	D(-3.01)	B(0.387)	24.7	0.325
chr16	*XYLT1*	17211722	c.2338G>A	p.V780M	1	0	1	34	0.000120202	D(0.007)	N(-1.79)	D(0.995)	27.3	0.235
chr16	*XYLT1*	17211748	c.2312A>C	p.K771T	1	1	0	1	3.97671E-06	T(0.067)	D(-3.54)	D(0.971)	24.3	0.212
chr16	*XYLT1*	17211799	c.2261G>A	p.R754H	2	1	1	18	6.38257E-05	D(0.001)	D(-4.73)	D(1)	31	0.738
chr16	*XYLT1*	17211833	c.2227G>A	p.G743S	2	1	1	4	1.61998E-05	T(0.225)	D(-4.45)	D(1)	25.1	0.361
chr16	*XYLT1*	17228367	c.1990G>A	p.E664K	3	0	3	9	3.18356E-05	T(0.755)	N(-0.44)	P(0.464)	23.4	0.173
chr16	*XYLT1*	17228372	c.1985G>A	p.R662Q	8	3	5	34	0.000120273	T(0.272)	N(-1.06)	B(0.257)	23.7	0.174
chr16	*XYLT1*	17228381	c.1976G>A	p.G659D	2	0	2	/	/	D(0.014)	D(-3.58)	D(1)	28.8	0.655

chr16	*XYLT1*	17228421	c.1936G>A	p.D646N	1	0	1	/	/	D(0)	D(-4.47)	D(0.967)	29.6	0.37	
chr16	*XYLT1*	17228439	c.1918G>A	p.G640S	6	2	4	154	0.000544447	T(0.203)	D(-2.87)	P(0.892)	25.1	0.236	
chr16	*XYLT1*	17228474	c.1883G>A	p.R628H	1	0	1	8	2.82833E-05	T(0.538)	N(-1.05)	B(0.055)	23	0.058	
chr16	*XYLT1*	17228483	c.1874C>T	p.P625L	1	0	1	27	9.54549E-05	D(0.035)	D(-5.8)	P(0.906)	24.7	0.283	
chr16	*XYLT1*	17228516	c.1829_1841del	p.G610Vfs*31	1	1	0	/	/	NA	NA	NA	NA		
chr16	*XYLT1*	17228520	c.1837G>T	p.D613Y	2	0	2	/	/	D(0)	D(-7.28)	D(0.991)	31	0.644	
chr16	*XYLT1*	17228550	c.1807G>A	p.V603M	1	0	1	57	0.000201546	D(0.034)	N(-1.06)	P(0.488)	23.3	0.051	
chr16	*XYLT1*	17228583	c.1774C>T	p.R592W	2	0	2	3	1.19386E-05	D(0)	D(-6.75)	D(0.969)	29.5	0.346	
chr16	*XYLT1*	17232210	c.1764+2T>C	splicing	1	0	1	/	/	NA	NA	NA	32	#NNAA	
chr16	*XYLT1*	17232219	c.1757G>A	p.R586H	1	0	1	5	1.99173E-05	D(0.001)	D(-4.69)	D(0.994)	31	0.525	
chr16	*XYLT1*	17232220	c.1756C>T	p.R586C	1	0	1	63	0.000223026	D(0)	D(-7.41)	D(0.994)	31	0.315	
chr16	*XYLT1*	17232259	c.1717G>A	p.G573S	1	0	1	23	8.13209E-05	D(0)	D(-5.8)	D(1)	27.9	0.578	
chr16	*XYLT1*	17232271	c.1705G>A	p.V569M	1	0	1	4	1.59066E-05	D(0)	D(-2.9)	D(0.984)	27.6	0.46	
chr16	*XYLT1*	17232306	c.1670G>A	p.R557H	1	0	1	4	1.59056E-05	D(0.001)	D(-4.83)	D(0.984)	31	0.333	
chr16	*XYLT1*	17232324	c.1652G>A	p.R551H	1	0	1	9	3.57878E-05	T(0.319)	N(-2.27)	D(0.97)	26.4	0.445	
chr16	*XYLT1*	17232353	c.1623C>G	p.H541Q	1	0	1	/	/	T(0.236)	N(-2.16)	P(0.876)	22.7	0.212	
chr16	*XYLT1*	17232375	c.1601C>T	p.T534M	3	1	2	16	6.36826E-05	D(0)	D(-5.83)	D(0.984)	28.8	0.716	
chr16	*XYLT1*	17235071	c.1523_1525delTCT	p.F508del	2	0	2	2	7.95311E-06	NA	NA	NA	NA		
chr16	*XYLT1*	17235132	c.1465G>A	p.V489M	1	0	1	9	3.18246E-05	T(0.064)	N(-1.9)	D(0.998)	25.2	0.246	Lee JK, et al.
chr16	*XYLT1*	17235180	c.1417G>A	p.A473T	1	0	1	/	/	T(1)	N(0.87)	B(0.011)	17.92	0.08	
chr16	*XYLT1*	17235207	c.1390C>G	p.L464V	1	1	0	/	/	D(0)	D(-2.9)	D(0.999)	27	0.309	
chr16	*XYLT1*	17252703	c.1353C>A	p.H451Q	1	0	1	/	/	D(0.001)	D(-7.69)	D(1)	22.6	0.397	
chr16	*XYLT1*	17252728	c.1328G>A	p.R443Q	2	0	2	13	4.59611E-05	D(0.015)	D(-3.75)	D(0.999)	29.6	0.304	
chr16	*XYLT1*	17252746	c.1310C>T	p.A437V	1	1	0	16	5.65723E-05	D(0.043)	D(-2.64)	D(0.985)	28.2	0.23	

chr16	*XYLT1*	17252749	c.1307T>C	p.V436A	1	0	1	11	4.37459E-05	D(0.009)	D(-3.39)	D(0.997)	27.2	0.362	
chr16	*XYLT1*	17292087	c.1271C>T	p.A424V	1	1	0	4	1.42148E-05	D(0.017)	D(-2.99)	D(0.993)	29.7	0.545	
chr16	*XYLT1*	17292121	c.1237G>A	p.D413N	1	0	1	15	5.31026E-05	T(0.196)	D(-2.37)	D(1)	27.1	0.245	
chr16	*XYLT1*	17292129	c.1229A>T	p.E410V	1	0	1	4	1.59258E-05	D(0.026)	D(-4.35)	P(0.577)	27.2	0.176	
chr16	*XYLT1*	17292142	c.1216C>T	p.R406W	2	1	1	20	7.07704E-05	D(0.002)	D(-4.64)	D(0.957)	25.6	0.293	
chr16	*XYLT1*	17292182	c.1176G>A	p.W392*	1	0	1	/	/	NA	NA	NA	43	#NNAA	
chr16	*XYLT1*	17292252	c.1106G>A	p.R369Q	1	0	1	3	1.19715E-05	D(0.005)	D(-3.81)	D(0.999)	32	0.365	
chr16	*XYLT1*	17294392	c.1033A>C	p.M345L	1	0	1	37	0.000130822	T(1)	N(1.1)	B(0.018)	17.74	0.122	
chr16	*XYLT1*	17294449	c.976G>T	p.V326F	1	0	1	/	/	D(0.001)	D(-4.35)	D(0.972)	28.2	0.481	
chr16	*XYLT1*	17352862	c.896G>A	p.R299Q	4	1	3	6	2.89226E-05	T(0.101)	D(-2.6)	D(0.996)	24.8	0.333	Lee JK, et al.
chr16	*XYLT1*	17352901	c.857G>A	p.R286H	1	0	1	1	5.56347E-06	T(0.102)	N(-1.84)	P(0.584)	23.7	0.11	
chr16	*XYLT1*	17352919	c.839T>C	p.I280T	1	0	1	2	9.30224E-06	D(0)	D(-4.93)	D(0.993)	26.7	0.662	
chr16	*XYLT1*	17352928	c.830G>A	p.R277H	1	0	1	5	2.28795E-05	D(0.003)	D(-3.29)	D(0.997)	29.6	0.341	
chr16	*XYLT1*	17353002	c.756C>A	p.D252E	1	0	1	/	/	T(0.321)	N(-0.34)	B(0.004)	19.54	0.104	
chr16	*XYLT1*	17353034	c.724A>G	p.T242A	3	0	3	7	2.56694E-05	T(0.316)	N(-0.15)	B(0.001)	6.661	0.015	
chr16	*XYLT1*	17353066	c.692A>G	p.K231R	3	0	3	4	1.59252E-05	T(0.318)	N(-0.27)	B(0.056)	19.59	0.13	
chr16	*XYLT1*	17353118	c.640G>A	p.G214S	2	1	1	5	1.98938E-05	T(0.242)	N(0.28)	B(0)	10.77	0.041	
chr16	*XYLT1*	17353201	c.557C>T	p.P186L	3	2	1	6	2.12116E-05	T(0.427)	N(-0.05)	B(0.001)	13.84	0.068	
chr16	*XYLT1*	17353318	c.440G>T	p.R147L	1	0	1	/	/	D(0.011)	N(-1.5)	P(0.652)	25.4	0.092	
chr16	*XYLT1*	17353333	c.425C>T	p.P142L	1	0	1	35	0.000124658	D(0)	D(-2.76)	D(0.998)	27	0.236	
chr16	*XYLT1*	17353340	c.418C>T	p.H140Y	1	0	1	/	/	D(0.002)	N(-1.68)	D(0.99)	27	0.229	
chr16	*XYLT1*	17353351	c.407G>A	p.G136D	1	0	1	/	/	D(0.024)	N(-1.73)	P(0.623)	24.7	0.107	
chr16	*XYLT1*	17451882	c.389C>T	p.T130I	3	0	3	11	3.89819E-05	T(0.051)	N(-0.33)	B(0.076)	20.4	0.031	
chr16	*XYLT1*	17451891	c.380C>T	p.P127L	1	0	1	7	2.79318E-05	D(0.018)	N(0.08)	D(0.997)	24	0.149	

chr16	*XYLT1*	17564563	c.91G>A	p.V31I	1	0	1	1	4.00994E-05	T(1)	N(-0.06)	B(0)	11.11	0.031	
chr02	*NDUFAF7*	37458854	c.6T>G	p.S2R	1	1	0	2	7.07564E-06	D(0.024)	N(0.33)	B(0.001)	0.846	0.029	
chr02	*NDUFAF7*	37459249	c.56C>T	p.A19V	1	1	0	10	3.97678E-05	T(0.296)	N(0.08)	B(0.0)	10.87	0.023	
chr02	*NDUFAF7*	37459288	c.95G>T	p.G32V	2	0	2	/	/	D(0.011)	N(-0.69)	P(0.731)	21.9	0.086	
chr02	*NDUFAF7*	37459344	c.151A>G	p.I51V	1	0	1	3	1.06045E-05	D(0.02)	N(-0.98)	B(0.32)	23.2	0.458	
chr02	*NDUFAF7*	37459396	c.203C>G	p.T68S	1	0	1	2	7.95425E-06	D(0.007)	D(-3.57)	P(0.589)	24.8	0.599	
chr02	*NDUFAF7*	37463255	c.233G>T	p.R78L	4	1	3	39	0.000138114	D(0.011)	D(-4.24)	B(0.118)	19.74	0.375	
chr02	*NDUFAF7*	37468815	c.503C>T	p.T168I	1	0	1	8	2.82963E-05	T(0.236)	N(-2.1)	P(0.496)	19.18	0.338	
chr02	*NDUFAF7*	37468866	c.554A>T	p.Y185F	1	0	1	/	/	D(0.006)	D(-3.41)	D(0.992)	23.6	0.292	
chr02	*NDUFAF7*	37468869	c.557T>C	p.M186T	1	0	1	/	/	T(0.141)	N(-1.02)	B(0.443)	22.2	0.198	
chr02	*NDUFAF7*	37468893	c.581T>A	p.I194N	2	0	2	6	2.45138E-05	D(0.001)	D(-4.15)	P(0.797)	27.4	0.578	
chr02	*NDUFAF7*	37468896	c.584C>T	p.P195L	1	1	0	/	/	D(0.009)	D(-6.02)	P(0.566)	27.6	0.701	
chr02	*NDUFAF7*	37469792	c.637C>G	p.L213V	17	0	17	75	0.000265858	T(0.254)	N(-0.92)	B(0.163)	20.3	0.32	
chr02	*NDUFAF7*	37473201	c.799G>A	p.E267K	6	0	6	17	6.77199E-05	T(0.108)	D(-2.53)	B(0.362)	26.1	0.376	
chr02	*NDUFAF7*	37474622	c.960T>A	p.H320Q	1	1	0	3	1.19339E-05	D(0.011)	D(-6.63)	D(0.99)	23	0.503	Liu F, et al.
chr02	*NDUFAF7*	37474765	c.1103G>T	p.R368L	1	0	1	6	2.14272E-05	D(0.0)	D(-6.94)	D(0.999)	31	0.916	
chr02	*NDUFAF7*	37474767	c.1105C>G	p.L369V	1	0	1	1	4.02379E-06	D(0.003)	D(-2.71)	P(0.688)	23	0.56	
chr02	*NDUFAF7*	37475432	c.1265C>T	p.A422V	1	0	1	1	3.97719E-06	D(0.029)	N(-0.29)	B(0.008)	11.38	0.072	
chr02	*NDUFAF7*	37475435	c.1268G>A	p.R423H	1	0	1	13	4.59621E-05	T(0.184)	N(-0.12)	B(0.103)	15.6	0.047	
chr08	*CPSF1*	145618552	c.4313G>A	p.R1438H	5	0	5	2	1.25356E-05	D(0.037)	D(-2.98)	D(0.972)	23.6	0.169	
chr08	*CPSF1*	145618558	c.4307C>T	p.T1436M	2	0	2	5	3.86739E-05	D(0.01)	N(0.72)	B(0.001)	23.7	0.107	
chr08	*CPSF1*	145618726	c.4225T>C	p.Y1409H	1	0	1	/	/	T(0.46)	N(0.29)	B(0.028)	22.4	0.193	
chr08	*CPSF1*	145618789	c.4162C>T	p.R1388C	1	0	1	2	8.61171E-06	T(0.073)	D(-3.51)	D(0.997)	23.1	0.317	
chr08	*CPSF1*	145618803	c.4148T>C	p.M1383T	1	0	1	/	/	T(1)	N(-0.35)	B(0.013)	20.8	0.237	

chr08	*CPSF1*	145618807	c.4146-2A>G	splicing	1	1	0	1	4.34938E-06	/	/	/	34	/	Ouyang JM, et al.
chr08	*CPSF1*	145618883	c.4144C>T	p.R1382W	1	0	1	6	3.42853E-05	D(0)	D(-7.14)	D(1)	33	0.714	
chr08	*CPSF1*	145618894	c.4133C>T	p.P1378L	1	0	1	/	/	D(0)	D(-7.56)	D(0.997)	26.9	0.543	
chr08	*CPSF1*	145618907	c.4120G>A	p.A1374T	27	3	24	18	8.75435E-05	D(0.008)	D(-3.08)	D(1)	24.7	0.397	
chr08	*CPSF1*	145618920	c.4107G>A	p.M1369I	2	0	2	1	5.55142E-06	T(0.337)	N(-1.45)	B(0.08)	19.1	0.118	
chr08	*CPSF1*	145618997	c.4030G>A	p.G1344S	1	0	1	8	3.49433E-05	T(0.954)	N(-0.5)	B(0.213)	18.76	0.196	
chr08	*CPSF1*	145619128	c.3985G>A	p.V1329M	1	0	1	9	3.22283E-05	T(0.265)	N(-0.11)	B(0.002)	15.45	0.022	
chr08	*CPSF1*	145619131	c.3982G>A	p.V1328I	1	0	1	118	0.000421947	T(0.46)	N(-0.15)	B(0.0)	15.88	0.095	
chr08	*CPSF1*	145619133	c.3980C>T	p.S1327L	7	4	3	117	0.000418225	T(0.293)	N(-2.12)	B(0.002)	23.2	0.075	
chr08	*CPSF1*	145619139	c.3974A>G	p.K1325R	1	0	1	/	/	T(0.654)	N(-0.6)	B(0.005)	20	0.061	
chr08	*CPSF1*	145619148	c.3965G>C	p.G1322A	3	0	3	1	4.0151E-06	T(0.679)	N(-0.63)	B(0.0)	17.91	0.069	
chr08	*CPSF1*	145619157	c.3956C>T	p.A1319V	2	0	2	9	3.61803E-05	T(0.615)	N(-1.38)	B(0.008)	20.8	0.115	
chr08	*CPSF1*	145619164	c.3949C>T	p.R1317W	1	0	1	11	4.41044E-05	D(0.007)	D(-4.7)	D(1)	27.4	0.465	
chr08	*CPSF1*	145619251	c.3862_3871dup	p.F1291*	1	1	0	/	/	NA	NA		NA	NA	Ouyang JM, et al.
chr08	*CPSF1*	145619333	c.3854C>T	p.P1285L	1	1	0	2	8.02369E-06	D(0.0)	D(-9.61)	D(1)	28.1	0.609	
chr08	*CPSF1*	145619342	c.3845T>C	p.M1282T	1	1	0	1	4.00921E-06	T(0.109)	D(-3.24)	B(0.274)	22.7	0.208	
chr08	*CPSF1*	145619364	c.3823G>T	p.D1275Y	1	1	0	/	/	D(0.002)	D(-4.39)	D(0.999)	25.2	0.492	Ouyang JM, et al.
chr08	*CPSF1*	145619364	c.3823G>A	p.D1275N	1	1	0	1	4.0151E-06	D(0.008)	N(-1.94)	P(0.784)	23.8	0.096	
chr08	*CPSF1*	145619367	c.3820C>T	p.R1274C	1	1	0	12	4.81928E-05	T(0.066)	D(-2.61)	D(0.998)	27.2	0.25	
chr08	*CPSF1*	145619479	c.3781A>G	p.M1261V	5	0	5	3	1.24259E-05	T(0.22)	N(-1.57)	B(0.003)	21.2	0.116	
chr08	*CPSF1*	145619488	c.3772G>T	p.V1258L	1	0	1	/	/	D(0.032)	N(-1.88)	B(0.218)	22.6	0.123	
chr08	*CPSF1*	145619626	c.3712G>A	p.E1238K	3	0	3	4	1.66287E-05	D(0.02)	D(-3.2)	P(0.731)	26	0.233	
chr08	*CPSF1*	145619674	c.3664A>C	p.I1222L	1	0	1	/	/	T(0.053)	N(-1.39)	B(0.173)	22.7	0.155	
chr08	*CPSF1*	145619696	c.3642G>C	p.Q1214H	1	0	1	1	4.15766E-06	T(0.073)	D(-3.14)	P(0.74)	23.2	0.22	

chr08	*CPSF1*	145619890	c.3536A>G	p.N1179S	1	0	1	1	4.10028E-06	T(0.761)	N(-1.37)	B(0.024)	16.98	0.117	
chr08	*CPSF1*	145619906	c.3520G>A	p.A1174T	3	0	3	10	3.5978E-05	D(0.019)	D(-3.12)	D(0.998)	27.1	0.422	
chr08	*CPSF1*	145620138	c.3373A>T	p.T1125S	2	0	2	/	/	D(0.0)	D(-3.71)	D(1)	26.1	0.493	
chr08	*CPSF1*	145620144	c.3367G>A	p.A1123T	1	1	0	8	0.000028685	D(0.029)	N(-0.64)	P(0.529)	23.1	0.148	
chr08	*CPSF1*	145620147	c.3364G>A	p.A1122T	1	0	1	/	/	D(0.04)	D(-2.72)	D(0.999)	24.5	0.261	
chr08	*CPSF1*	145620150	c.3361G>A	p.V1121M	1	0	1	5	2.01665E-05	D(0.005)	N(-1.16)	P(0.942)	25.6	0.189	
chr08	*CPSF1*	145620171	c.3340G>A	p.V1114M	4	0	4	4	1.43122E-05	T(0.237)	N(0.08)	P(0.725)	23	0.139	
chr08	*CPSF1*	145620176	c.3335A>G	p.E1112G	1	0	1	1	4.0318E-06	T(0.651)	N(1.67)	B(0.004)	22.6	0.204	
chr08	*CPSF1*	145620194	c.3317T>C	p.V1106A	1	1	0	/	/	T(0.094)	D(-3.41)	D(0.998)	25.8	0.505	
chr08	*CPSF1*	145620235	c.3276C>G	p.I1092M	1	0	1	2	7.23223E-06	T(0.124)	N(-1.42)	P(0.493)	17.56	0.1	
chr08	*CPSF1*	145620353	c.3232C>T	p.L1078F	2	0	2	/	/	D(0.003)	D(-3.64)	D(1)	27.5	0.495	
chr08	*CPSF1*	145620359	c.3226A>G	p.I1076V	1	0	1	/	/	T(0.465)	N(-0.53)	B(0.246)	21.3	0.113	
chr08	*CPSF1*	145620542	c.3125C>T	p.T1042M	22	0	22	23	9.19647E-05	D(0.029)	N(-1.19)	B(0.007)	22.3	0.131	
chr08	*CPSF1*	145620545	c.3122A>G	p.N1041S	1	0	1	1	3.9991E-06	T(0.935)	N(0.54)	B(0.0)	13.31	0.137	
chr08	*CPSF1*	145621588	c.2954G>A	p.R985H	6	0	6	34	0.000141333	T(0.164)	N(-0.67)	B(0.001)	14.74	0.269	
chr08	*CPSF1*	145621637	c.2905G>A	p.D969N	1	0	1	/	/	T(0.196)	D(-3.77)	P(0.933)	23.1	0.283	
chr08	*CPSF1*	145621657	c.2885G>A	p.R962Q	1	0	1	7	3.17676E-05	D(0.01)	D(-3.01)	P(0.909)	24.2	0.256	
chr08	*CPSF1*	145621658	c.2884C>T	p.R962W	1	0	1	4	1.80719E-05	D(0.001)	D(-6.7)	D(1)	28.5	0.59	
chr08	*CPSF1*	145621686	c.2856G>T	p.W952C	1	0	1	/	/	D(0.001)	D(-9.33)	D(1)	27.5	0.635	
chr08	*CPSF1*	145621814	c.2823_2824del	p.V943Lfs*65	1	1	0	22	7.83839E-05	NA	NA	NA	NA	NA	Ouyang JM, et al.
chr08	*CPSF1*	145621859	c.2780G>A	p.R927H	7	1	6	14	4.97728E-05	D(0.01)	N(-1.89)	P(0.75)	23.4	0.124	
chr08	*CPSF1*	145621865	c.2774G>A	p.R925Q	1	0	1	7	2.48853E-05	T(0.654)	N(-0.01)	B(0.017)	21.7	0.062	
chr08	*CPSF1*	145621866	c.2773C>G	p.R925G	2	0	2	/	/	T(0.339)	N(-1.77)	B(0.0)	22.7	0.081	
chr08	*CPSF1*	145621869	c.2770G>A	p.A924T	1	1	0	150	0.00053317	T(0.398)	N(0.1)	B(0.0)	11.05	0.025	

chr08	*CPSF1*	145622087	c.2650G>A	p.G884S	2	0	2	8	2.83628E-05	T(0.846)	N(0.85)	B(0.0)	15.39	0.022	
chr08	*CPSF1*	145622434	c.2580delG	p.R860Sfs*96	1	0	1	/	/	NA	NA	NA	NA	NA	
chr08	*CPSF1*	145622445	c.2569C>T	p.R857C	6	1	5	12	6.57239E-05	T(0.078)	D(-3.89)	P(0.927)	22.8	0.18	
chr08	*CPSF1*	145622447	c.2567G>T	p.S856I	1	0	1	/	/	T(0.379)	N(-1.22)	B(0.0)	19.36	0.083	
chr08	*CPSF1*	145622450	c.2564G>T	p.G855V	2	0	2	/	/	D(0)	D(-8.49)	D(1)	24.5	0.639	
chr08	*CPSF1*	145622498	c.2516G>A	p.R839H	1	0	1	2	1.3129E-05	T(0.128)	N(-0.82)	D(0.997)	24.1	0.16	
chr08	*CPSF1*	145622501	c.2513C>T	p.T838M	1	0	1	141	0.000764534	T(0.154)	N(-1.15)	P(0.954)	23	0.082	
chr08	*CPSF1*	145622613	c.2401G>A	p.D801N	1	0	1	2	1.097E-05	D(0.014)	D(-3.92)	D(0.992)	26.6	0.428	
chr08	*CPSF1*	145622722	c.2365C>T	p.R789W	1	0	1	24	9.04146E-05	D(0)	D(-7.33)	D(1)	31	0.378	
chr08	*CPSF1*	145622764	c.2323G>A	p.A775T	1	0	1	/	/	T(0.65)	N(-0.55)	B(0.0)	12.64	0.034	
chr08	*CPSF1*	145622767	c.2320C>T	p.P774S	1	0	1	/	/	T(0.574)	N(-0.73)	B(0.003)	19.78	0.066	
chr08	*CPSF1*	145622772	c.2315G>A	p.R772Q	4	0	4	4	1.64391E-05	T(0.537)	N(-0.42)	B(0.01)	22	0.032	
chr08	*CPSF1*	145622773	c.2314C>T	p.R772W	6	0	6	8	3.78544E-05	T(0.217)	N(-0.79)	D(0.98)	23.3	0.139	
chr08	*CPSF1*	145622795	c.2292A>C	p.R764S	5	0	5	96	0.00041301	T(0.275)	N(-0.81)	B(0.033)	20.6	0.173	
chr08	*CPSF1*	145622835	c.2252C>T	p.S751L	6	0	6	7	3.59059E-05	D(0.007)	D(-3.61)	P(0.915)	25.4	0.163	Zheng YH, et al.
chr08	*CPSF1*	145622998	c.2170C>T	p.R724C	4	0	4	22	8.10498E-05	D(0.046)	N(-2.21)	D(0.977)	23.5	0.117	
chr08	*CPSF1*	145623004	c.2164_2165insGG	p.R724Afs*86	1	0	1	/	/	NA	NA	NA	NA	NA	
chr08	*CPSF1*	145623007	c.2161C>G	p.L721V	1	1	0	7	2.90028E-05	T(0.545)	N(-0.07)	B(0.009)	9.008	0.007	
chr08	*CPSF1*	145623013	c.2155G>C	p.D719H	1	0	1	/	/	D(0.023)	N(-1.92)	P(0.576)	22.8	0.084	
chr08	*CPSF1*	145623015	c.2153G>A	p.R718H	1	0	1	2	8.24328E-06	T(0.093)	N(-0.87)	B(0.001)	22.4	0.073	
chr08	*CPSF1*	145623016	c.2152C>T	p.R718C	1	0	1	6	2.47243E-05	D(0.041)	N(-2.26)	B(0.203)	24.5	0.092	
chr08	*CPSF1*	145623066	c.2102A>T	p.Y701F	1	0	1	/	/	D(0.03)	D(-3.43)	D(0.999)	24.3	0.234	
chr08	*CPSF1*	145623078	c.2090C>T	p.T697M	1	0	1	4	1.61633E-05	D(0.024)	N(-1.97)	P(0.482)	22.7	0.158	
chr08	*CPSF1*	145623087	c.2081A>G	p.K694R	2	0	2	1	4.04655E-06	T(0.64)	N(-0.99)	B(0.001)	18.57	0.09	

chr08	*CPSF1*	145623194	c.2048C>T	p.A683V	1	0	1	1	3.19836E-05	T(0.476)	N(-1.41)	B(0.022)	22.8	0.114	
chr08	*CPSF1*	145623209	c.2033G>A	p.R678H	1	0	1	5	1.80074E-05	T(0.148)	N(-0.84)	B(0.002)	22.9	0.065	
chr08	*CPSF1*	145623210	c.2032C>T	p.R678C	8	1	7	20	7.18943E-05	D(0.022)	N(-1.12)	P(0.857)	23.2	0.125	
chr08	*CPSF1*	145623216	c.2026G>A	p.G676S	1	0	1	5	1.79107E-05	T(0.171)	N(-0.59)	B(0.047)	17.48	0.046	
chr08	*CPSF1*	145623230	c.2012delA	p.K671Rfs*25	1	0	1	/	/	NA	NA	NA	NA	NA	
chr08	*CPSF1*	145623266	c.1976T>C	p.M659T	5	0	5	3	1.20504E-05	D(0.012)	D(-3.16)	P(0.623)	23.9	0.432	
chr08	*CPSF1*	145623278	c.1964A>G	p.Y655C	1	0	1	3	1.20649E-05	D(0.001)	D(-8.57)	D(1.0)	25.9	0.346	
chr08	*CPSF1*	145623312	c.1930G>A	p.A644T	1	0	1	3	1.22508E-05	T(0.091)	N(-0.88)	B(0.007)	18.23	0.057	
chr08	*CPSF1*	145623598	c.1882_1894del	p.L628*	2	0	2	/	/	NA	NA	NA	NA	NA	
chr08	*CPSF1*	145623719	c.1867C>A	p.P623T	1	1	0	/	/	T(0.52)	D(-2.62)	B(0.213)	22	0.231	
chr08	*CPSF1*	145623728	c.1858C>T	p.Q620*	1	1	0	/	/	NA	NA	NA	39	NA	Ouyang JM, et al.
chr08	*CPSF1*	145623746	c.1840G>T	p.D614Y	1	0	1	/	/	D(0.019)	D(-4.68)	D(0.984)	31	0.386	
chr08	*CPSF1*	145623749	c.1837G>A	p.G613R	1	0	1	8	3.18312E-05	D(0.001)	D(-5.83)	D(1.0)	30	0.507	
chr08	*CPSF1*	145623751	c.1835T>C	p.I612T	1	0	1	/	/	D(0.005)	D(-3.61)	D(0.971)	26.7	0.608	
chr08	*CPSF1*	145623949	c.1718G>A	p.G573E	1	0	1	/	/	D(0.024)	N(-1.83)	P(0.872)	23.2	0.28	
chr08	*CPSF1*	145623991	c.1676C>T	p.P559L	1	0	1	3	9.56328E-05	D(0.012)	N(-2.17)	B(0.007)	15.46	0.119	
chr08	*CPSF1*	145624009	c.1658A>T	p.E553V	1	0	1	/	/	D(0.047)	N(-2.38)	B(0.189)	23.4	0.103	
chr08	*CPSF1*	145624021	c.1646A>G	p.N549S	4	0	4	6	2.12483E-05	T(0.696)	N(-0.13)	B(0.0)	4.441	0.018	
chr08	*CPSF1*	145624180	c.1627C>T	p.R543C	1	0	1	5	0.000018171	D(0.046)	N(-2.04)	D(0.981)	25	0.159	
chr08	*CPSF1*	145624195	c.1612G>C	p.V538L	2	0	2	3	1.21036E-05	T(0.089)	N(-2.32)	P(0.938)	25.6	0.168	
chr08	*CPSF1*	145624552	c.1433C>T	p.A478V	12	0	12	16	6.26846E-05	T(1)	N(0.46)	B(0.085)	15.63	0.207	
chr08	*CPSF1*	145624697	c.1361C>T	p.S454L	1	0	1	2	8.02227E-06	T(0.192)	D(-2.92)	D(0.989)	25.5	0.2	
chr08	*CPSF1*	145624745	c.1313C>T	p.P438L	1	0	1	15	5.33375E-05	T(0.866)	N(-0.62)	B(0.0)	15.36	0.212	
chr08	*CPSF1*	145624866	c.1270G>A	p.V424M	5	0	5	7	2.49636E-05	T(0.081)	N(-0.84)	B(0.034)	21.2	0.179	

chr08	*CPSF1*	145624983	c.1237G>A	p.D413N	2	0	2	38	0.000139144	T(0.307)	N(-0.31)	P(0.924)	20.8	0.114	
chr08	*CPSF1*	145625060	c.1160G>A	p.R387H	6	1	5	8	3.32734E-05	D(0)	D(-4.69)	D(0.996)	32	0.352	
chr08	*CPSF1*	145625082	c.1138G>A	p.G380R	1	0	1	4	1.67531E-05	D(0.037)	D(-4)	D(0.989)	24.6	0.469	
chr08	*CPSF1*	145625214	c.1076C>T	p.A359V	1	0	1	5	2.61876E-05	T(0.15)	N(-2.14)	B(0.06)	14.77	0.069	
chr08	*CPSF1*	145625418	c.995A>G	p.Y332C	1	0	1	/	/	T(0.183)	D(-3.12)	P(0.90)	24.2	0.221	
chr08	*CPSF1*	145625440	c.973G>A	p.A325T	2	0	2	3	1.23001E-05	T(0.094)	N(-1.87)	P(0.638)	20.9	0.216	
chr08	*CPSF1*	145625560	c.937C>G	p.R313G	2	0	2	/	/	T(0.179)	D(-4.25)	B(0.116)	20.2	0.14	
chr08	*CPSF1*	145625605	c.892G>A	p.V298M	1	1	0	/	/	D(0.003)	N(-2.24)	D(0.999)	24.6	0.233	
chr08	*CPSF1*	145625650	c.847G>A	p.V283I	2	0	2	2	7.33181E-06	D(0.039)	N(-0.82)	D(0.979)	27.2	0.156	
chr08	*CPSF1*	145625656	c.841T>G	p.F281V	1	0	1	7	2.53975E-05	T(0.25)	D(-2.89)	B(0.213)	23.3	0.24	
chr08	*CPSF1*	145626088	c.663T>G	p.F221L	1	0	1	/	/	D(0.014)	D(-4.23)	P(0.943)	19.03	0.267	
chr08	*CPSF1*	145626123	c.628C>T	p.H210Y	1	0	1	/	/	T(0.069)	D(-3.91)	B(0.02)	19.99	0.195	
chr08	*CPSF1*	145626141	c.610A>G	p.I204V	14	2	12	6	2.40408E-05	T(0.173)	N(-0.56)	B(0.002)	5.309	0.042	
chr08	*CPSF1*	145626174	c.577G>T	p.V193L	1	0	1	1	4.00279E-06	T(1)	N(1.01)	B(0.002)	14.96	0.253	
chr08	*CPSF1*	145626174	c.577G>A	p.V193M	1	0	1	15	5.33424E-05	D(0.007)	N(-0.22)	P(0.63)	22.4	0.286	
chr08	*CPSF1*	145626438	c.419G>A	p.R140Q	13	6	7	235	0.000861437	T(0.255)	N(-0.13)	B(0.253)	16.43	0.08	
chr08	*CPSF1*	145626616	c.376C>G	p.P126A	1	0	1	7	3.75319E-05	T(0.288)	N(-1.49)	B(0.0)	19.81	0.074	
chr08	*CPSF1*	145626664	c.328C>T	p.P110S	1	0	1	5	3.07072E-05	D(0.013)	D(-4.46)	P(0.885)	23.6	0.31	
chr08	*CPSF1*	145626667	c.325G>A	p.D109N	1	0	1	1	6.08872E-06	T(0.075)	D(-3.04)	P(0.931)	23.7	#NNAA	
chr08	*CPSF1*	145626832	c.298G>A	p.D100N	1	0	1	/	/	D(0.005)	D(-2.99)	D(0.993)	25.1	0.168	
chr08	*CPSF1*	145626889	c.241A>G	p.M81V	1	0	1	11	3.90642E-05	D(0.01)	N(-2.38)	B(0.112)	20.9	0.239	
chr08	*CPSF1*	145626931	c.199G>A	p.E67K	1	0	1	4	1.42605E-05	D(0.009)	D(-3.08)	D(0.964)	23.8	0.277	
chr08	*CPSF1*	145634494	c.49T>A	p.S17T	1	0	1	/	/	T(0.139)	N(-1.32)	B(0.053)	9.883	0.219	
chr08	*CPSF1*	145634528	c.15C>G	p.Y5*	1	1	0	/	/	NA	NA	NA	36	NA	Ouyang JM, et al.

chr06	*TNFRSF21*	47200589	c.1880G>A	p.R627Q	1	0	1	3	1.19325E-05	T(0.095)	N(-1.03)	B(0.02)	23.5	0.123
chr06	*TNFRSF21*	47200631	c.1838G>A	p.R613Q	4	0	4	3	1.0609E-05	D(0.005)	N(-0.39)	B(0.079)	23.4	0.149
chr06	*TNFRSF21*	47200703	c.1766T>G	p.V589G	20	3	17	/	/	D(0.000)	N(-2.27)	D(0.999)	27.4	0.628
chr06	*TNFRSF21*	47202424	c.1720G>A	p.G574S	4	0	4	5	1.82498E-05	D(0.029)	N(-1.94)	D(0.999)	29.7	0.502
chr06	*TNFRSF21*	47202496	c.1648T>C	p.F550L	1	0	1	4	1.44877E-05	D(0)	N(-1.95)	P(0.837)	25.5	0.561
chr06	*TNFRSF21*	47202537	c.1607T>A	p.L536Q	1	0	1	1	4.69096E-06	D(0.001)	N(-1.42)	B(0.23)	22.6	0.332
chr06	*TNFRSF21*	47202564	c.1580A>G	p.N527S	1	0	1	180	0.000892769	T(0.304)	N(-0.38)	B(0.001)	4.572	0.115
chr06	*TNFRSF21*	47221033	c.1468G>A	p.V490I	1	0	1	1	3.99974E-06	D(0)	N(-0.36)	P(0.685)	26.2	0.435
chr06	*TNFRSF21*	47221096	c.1405C>T	p.R469W	1	0	1	7	2.78976E-05	D(0)	D(-2.85)	D(0.987)	32	0.815
chr06	*TNFRSF21*	47221146	c.1355A>G	p.Y452C	1	0	1	2	7.9552E-06	D(0.044)	D(-3.54)	B(0.152)	23.6	0.657
chr06	*TNFRSF21*	47221225	c.1276G>C	p.V426L	1	0	1	2	7.98E-06	D(0.002)	N(-0.99)	P(0.498)	25.1	0.476
chr06	*TNFRSF21*	47251754	c.1163T>C	p.I388T	1	0	1	1	3.97719E-06	D(0.044)	N(-2.09)	B(0.043)	24.1	0.457
chr06	*TNFRSF21*	47251873	c.1044G>C	p.E348D	1	0	1	28	9.90211E-05	D(0)	N(-0.77)	D(0.994)	24.1	0.369
chr06	*TNFRSF21*	47252055	c.862G>A	p.V288M	4	0	4	235	0.0008758	T(0.525)	N(0.27)	B(0.002)	0.49	0.062
chr06	*TNFRSF21*	47252061	c.856G>A	p.E286K	1	1	0	2	8.54139E-06	D(0.045)	N(-0.49)	B(0.005)	19.61	0.11
chr06	*TNFRSF21*	47252157	c.760A>G	p.T254A	2	0	2	1	5.62924E-06	T(0.071)	N(-1.11)	B(0.15)	18.37	0.129
chr06	*TNFRSF21*	47253739	c.689T>C	p.F230S	1	0	1	/	/	T(0.179)	N(-0.42)	B(0.001)	15.23	0.029
chr06	*TNFRSF21*	47253757	c.671C>G	p.S224C	2	0	2	/	/	D(0.001)	N(-0.77)	B(0.319)	18.46	0.101
chr06	*TNFRSF21*	47253779	c.649T>G	p.F217V	1	0	1	/	/	T(0.174)	N(-1.09)	B(0)	5.701	0.022
chr06	*TNFRSF21*	47253823	c.605C>T	p.P202L	1	0	1	4	1.59095E-05	T(0.092)	D(-4.68)	B(0.002)	21.6	0.102
chr06	*TNFRSF21*	47253832	c.596T>G	p.V199G	1	0	1	5	1.99283E-05	D(0)	D(-5.14)	B(0.282)	25.8	0.354
chr06	*TNFRSF21*	47254004	c.424G>A	p.A142T	3	0	3	5	1.99445E-05	T(0.228)	N(-0.23)	B(0)	16.27	0.145
chr06	*TNFRSF21*	47254136	c.292A>T	p.R98W	3	1	2	5	1.99445E-05	D(0)	D(-4.01)	D(0.915)	29.2	0.747
chr06	*TNFRSF21*	47254169	c.259G>A	p.V87I	1	1	0	17	6.02486E-05	T(0.075)	N(-0.83)	B(0.088)	22.7	0.227

chr06	*TNFRSF21*	47254253	c.175C>T	p.R59C	1	1	0	12	4.24845E-05	D(0.008)	N(-2.21)	P(0.685)	27	0.608
chr06	*TNFRSF21*	47254302	c.126G>C	p.Q42H	1	0	1	/	/	D(0.012)	N(-1.86)	P(0.887)	23.2	0.212
chr06	*TNFRSF21*	47254323	c.105C>A	p.F35L	1	0	1	/	/	T(0.169)	N(-0.55)	B(0.001)	16.86	0.037
chr06	*TNFRSF21*	47277195	c.53C>G	p.A18G	2	0	2	/	/	D(0.01)	N(-0.42)	B(0.011)	13.17	0.028

Abbreviations: / indicated none in the gnomAD database; B = benign; D = damage; N = neutral; NA = not applicable; P = probably damage; T = tolerant.
