# Supplementary material for: Genetic and clinical landscape of ARR3-associated MYP26: the most common cause of Mendelian early-onset high myopia with a unique inheritance
Source: Br J Ophthalmol. 2022 Sep 30;107(10):1545–53. doi: 10.1136/bjo-2022-321511 (PMC10579186; doi:10.1136/bjo-2022-321511)
Supplement: Supplementary data [file bjo-2022-321511supp007.pdf]

Supplementary Table 2.The distribution of potential pathogenic variants in 14 genes among eoHM, control, and gnomAD (EA) groups.

Gene	Group	Number of subjects		*P* - value (*P*- corrected)
		Variant	Wild-type	
ARR3	eoHM	29	899	
	Control	0	6386	**6.770E-27 (9.477E-26)**
	gnomAD(EA)	1	13854	**2.550E-34 (3.570E-33)**
OPN1LW	eoHM	22	906	
	Control	0	6386	**1.512E-20 (2.116E-19)**
	gnomAD(EA)	0	14781	**7.383E-28 (1.034E-26)**
LRPAP1	eoHM	2	1854	
	Control	0	12772	**0.016 (0.224)***
	gnomAD(EA)	0	28222	**0.004 (0.054)***
SCO2	eoHM	5	923	
	Control	20	6366	0.237 (3.318)
	gnomAD(EA)	22	9953	0.075 (1.05)
SLC39A5	eoHM	1	927	
	Control	35	6351	0.079 (1.106)
	gnomAD(EA)	15	9961	1 (14)
P4HA2	eoHM	4	924	
	Control	50	6336	0.307 (4.298)
	gnomAD(EA)	64	9913	0.66 (6.0984)
BSG	eoHM	4	924	
	Control	41	6345	0.593 (8.302)
	gnomAD(EA)	12	9737	0.044 (0.616)
DZIP1	eoHM	7	921	
	Control	25	6361	0.115 (1.61)
	gnomAD(EA)	34	9941	0.082 (1.148)
XYLT1	eoHM	8	920	
	Control	37	6349	0.267 (3.738)
	gnomAD(EA)	52	9923	0.166 (2.324)
NDUFAF7	eoHM	2	926	
	Control	6	6380	0.27 (3.78)
	gnomAD(EA)	17	9943	0.674 (9.436)
CPSF1	eoHM	6	922	
	Control	5	6379	0.001 (0.014)#
	gnomAD(EA)	2	9937	0.000009 (0.000126)#
TNFRSF21	eoHM	2	926	
	Control	11	6375	0.676 (9.464)
	gnomAD(EA)	12	9961	0.337 (4.718)
CDCC111	eoHM	18	910	
	Control	183	6203	0.131 (1.834)
	gnomAD(EA)	406	9570	0.001 (0.014)
ZNF644	eoHM	8	920	
	Control	123	6263	0.023 (0.322)
	gnomAD(EA)	249	9728	0.002 (0.028)

Abbreviations: Red cells represents that potential pathogenic variants clustered in eoHM group compared with control or gnomAD(EA) database. Gery cells represents no significantly statistic differences of distribution of specific variants between eoHM group and control or gnomAD(EA) database.Blue cells represents potential pathogenic varaints clustered in gnomAD(EA) families than eoHM group. eoHM = early-onset high myopia; EA = East Asian; * As a recessive disease-causing gene, the comparitive analysis is not not applicable for *LRPAP1* . Only one family with *LRPAP1* frameshift variant in homozygous status was detected in eoHM group, and no family in homozygous or compound heterozygous status in 6386 control families or gmonAD.# Though *CPSF1* mutations were reported to be associated with eoHM in our previous study, a few individuals with truncation in *CPSF1* but no myopia in our furthur study reminds us to be caution in determining and assessing *CPSF1* mutations.
