# Supplementary material for: Genetic and clinical landscape of ARR3-associated MYP26: the most common cause of Mendelian early-onset high myopia with a unique inheritance
Source: Br J Ophthalmol. 2022 Sep 30;107(10):1545–53. doi: 10.1136/bjo-2022-321511 (PMC10579186; doi:10.1136/bjo-2022-321511)
Supplement: Supplementary data [file bjo-2022-321511supp008.pdf]

Supplementary Table 3. Twenty-four pathogenic variants in *ARR3* that identified in our cohort (including three previously reported)

No.	Position(at chrX) (GRCh37/hg19)	NM_004312.2 Change	NM_004312.2 Effect	Family Count	ACMG/AMP Evidence	CA	gnomAD AF	REVEL	CADD	SIFT	Poly-phen2	PROVEAN	BDGP	NetGene2	HSF
1	69489238	c.3G>A	p.M1I?	1	PVS1,PS4,PM2,PP1,PP4	P	none	NA	0.01	NA	NA	NA	NSSC	NSSC	NSSC
2	69489511	c.9-1G>A	SA	1	PVS1,PS4,PM2,PP1	P	none	NA	22.7	NA	NA	NA	SSC	SSC	SSC
3	69489952	c.103G>A	p.G35S	1	PS2,PS4,PM2,PP3	P	none	0.54	28.6	D	D	D	NSSC	NSSC	SSC
4	69489988	c.139C>T	p.R47*	2	PVS1,PS2,PS4,PM2,PP4	P	none	NA	35.0	NA	NA	NA	NSSC	NSSC	NSSC
5	69495932	c.146T>G	p.L49W	1	PS4,PM2,PP1,PP3,PP4	LP	none	0.21	24.1	D	Pr	D	NSSC	NSSC	NSSC
6	69495935	c.149T>C	p.F50S	1	PS4,PM2,PP1,PP3,PP4	LP	5.5E-06	0.43	23.9	D	D	D	NSSC	NSSC	NSSC
7	69496018	c.232C>T	p.Q78*	1	PVS1,PS4,PM2,PP1,PP4	P	none	NA	32.0	NA	NA	NA	SSC	NSSC	NSSC
8	69496025	c.239T>C	p.L80P	1	PS1,PS4,PM2,PP1,PP4	P	none	0.08	9.2	T	B	D	NSSC	SSC	NSSC
9	69496084	c.298C>T	p.R100*	3	PVS1,PS1,PS4,PM2	P	none	NA	34.0	NA	NA	NA	SSC	NSSC	SSC
10	69496131	c.345G>C	p.Q115H	1	PVS1,PS4,PM2	P	none	0.07	34.0	T	Pr	N	SSC	SSC	SSC
11	69496281	c.346-2A>T	SA	1	PVS1,PS4,PM2,PP4	P	none	NA	33.0	NA	NA	NA	SSC	SSC	SSC
12	69496298	c.361C>A	p.P121T	1	PS4,PM2,PM6,PP3	LP	none	0.63	25.3	D	D	D	NSSC	NSSC	NSSC
13	69496323	c.386_389del	4	1	PVS1,PS4,PM2	P	none	NA	NA	NA	NA	NA	NSSC	NSSC	SSC
14	69497269	c.499A>T	p.K167*	1	PVS1,PS4,PM2,PP4	P	none	NA	37.0	NA	NA	NA	SSC	NSSC	SSC
15	69497290	c.520G>T	p.E174*	1	PVS1,PS4,PM2	P	none	NA	35.0	NA	NA	NA	NSSC	NSSC	SSC
16	69497290	c.520delG	0	1	PVS1,PS4,PM2,PM4,PP4	P	none	NA	NA	NA	NA	NA	NSSC	NSSC	SSC
17	69497928	c.707C>G	p.T236R	1	PS4,PM2,PM6,PP3	LP	none	0.33	24.7	D	D	D	NSSC	NSSC	SSC
18	69497978	c.757delC	p.Q253Rfs*7	1	PVS1,PS4,PM2,PP4	P	none	NA	NA	NA	NA	NA	NSSC	NSSC	SSC
19	69498430	c.844_845insT	p.R282Lfs*10	1	PVS1,PS4,PM2,PP1,PP4	P	none	NA	NA	NA	NA	NA	NSSC	NSSC	NSSC
20	69498479	c.893C>A	p.A298D	1	P4	LP	none	0.63	26.2	D	D	D	NSSC	NSSC	NSSC
21	69500067	c.928G>T	p.E310*	1	PVS1,PS4,PM2,PP1,PP4	P	none	NA	38.0	NA	NA	NA	NSSC	NSSC	NSSC
22	69500068	c.929_930del	6	1	PVS1,PS4,PM2	P	none	NA	NA	NA	NA	NA	NSSC	NSSC	SSC
23	69500102	c.963_964del	5	3	PVS1,PS4,PM2,PP4	P	none	NA	NA	NA	NA	NA	NSSC	Un	SSC
24	69500614	c.1014-2A>G	SA	1	PVS1,PS4,PS2,PM2	P	none	NA	32.0	NA	NA	NA	SSC	SSC	SSC

Abbreviations: AF = allele Frequency; B = benign; CA = classification; D = damage; N = neutral; NA = not applicable; NSSC = no splicing site change; P = pathogenic; Pr = probably damage; T = tolerant; Un = unknown splicing effect; SA = splicing acceptor; SSC = splicing site change. The p.L80P, p.R100*, and p.A298D were reported before.
