# Supplementary material for: Genetic and clinical landscape of ARR3-associated MYP26: the most common cause of Mendelian early-onset high myopia with a unique inheritance
Source: Br J Ophthalmol. 2022 Sep 30;107(10):1545–53. doi: 10.1136/bjo-2022-321511 (PMC10579186; doi:10.1136/bjo-2022-321511)
Supplement: Supplementary data [file bjo-2022-321511supp001.pdf]

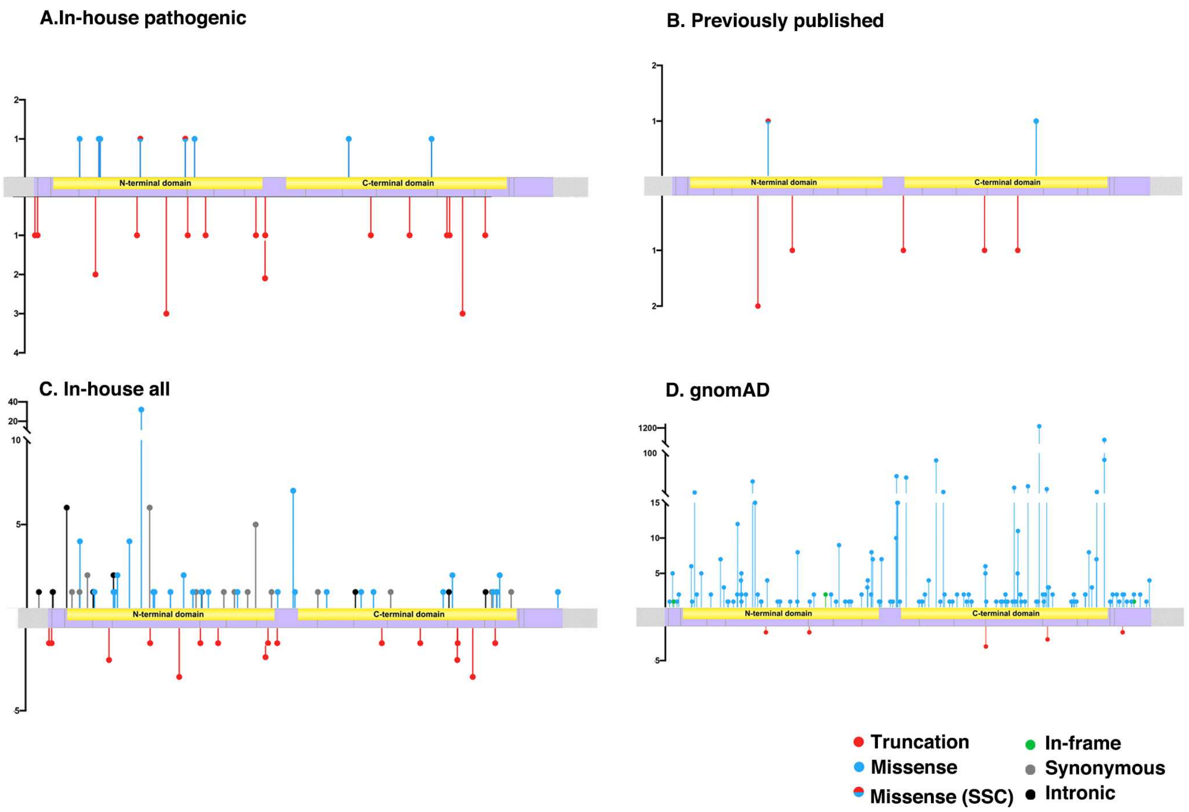

Supplementary Figure 1. The distribution and allele count of variants in *ARR3* based on in-house exome sequencing data, published literature, as well as general population dataset. (A) Pathogenic variants in *ARR3* identified in our cohort; (B) previously published variants; (C) all detected variants in *ARR3* based on our exome sequencing database; and (D) gnomAD database. The position and allele count of truncation variants are drawn below the mRNA structure diagram, whereas those of missense variants, synonymous variants, intronic changes in the splicing regions without splicing site changes effect, and in-frame variants are shown above the mRNA structure diagram. SSC: splicing site changes.
