# Supplementary material for: Genetic and clinical landscape of ARR3-associated MYP26: the most common cause of Mendelian early-onset high myopia with a unique inheritance
Source: Br J Ophthalmol. 2022 Sep 30;107(10):1545–53. doi: 10.1136/bjo-2022-321511 (PMC10579186; doi:10.1136/bjo-2022-321511)
Supplement: Supplementary data [file bjo-2022-321511supp002.pdf]

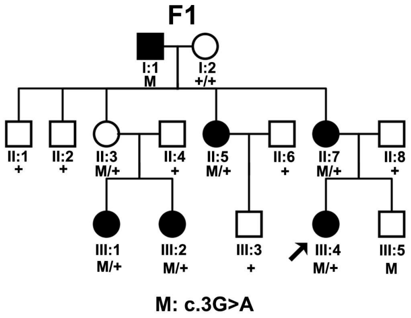
F1
I:1
M
I:2
+/+
II:1
+
II:2
+
II:3
M/+
II:4
+
II:5
M/+
II:6
+
II:7
M/+
II:8
+
III:1
M/+
III:2
M/+
III:3
+
III:4
M/+
III:5
M
M: c.3G>A

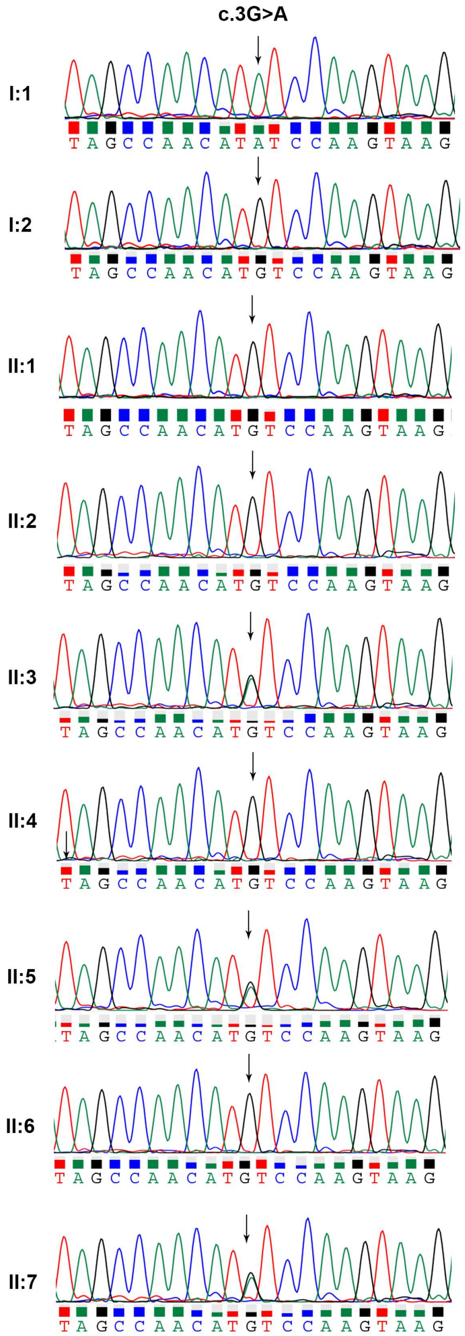
c.3G>A
I:1
TAGCCAACATATCCAAGTAAG
I:2
TAGCCAACATGTCCAAGTAAG
II:1
TAGCCAACATGTCCAAGTAAG
II:2
TAGCCAACATGTCCAAGTAAG
II:3
TAGCCAACATGTCCAAGTAAG
II:4
TAGCCAACATGTCCAAGTAAG
II:5
TAGCCAACATGTCCAAGTAAG
II:6
TAGCCAACATGTCCAAGTAAG
II:7
TAGCCAACATGTCCAAGTAAG

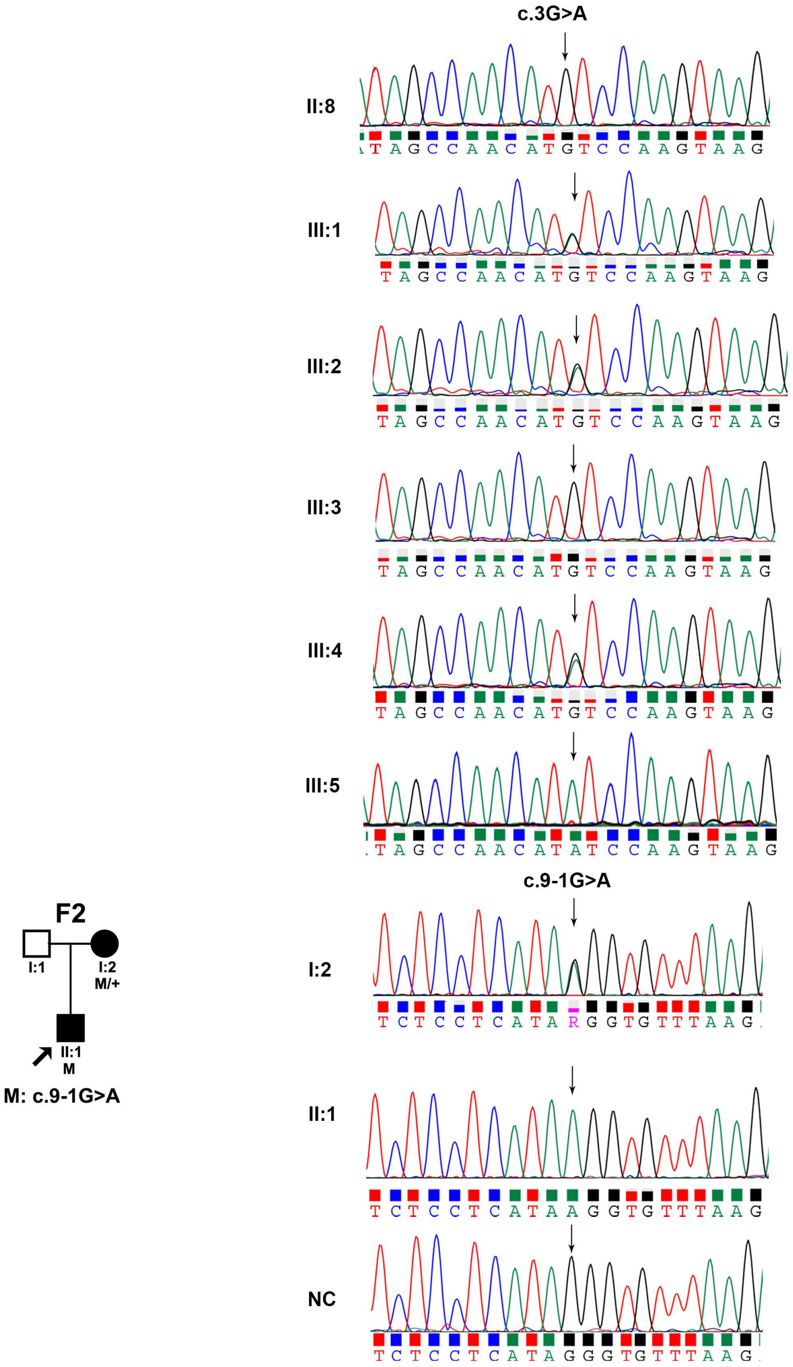
c.3G>A
II:8
III:1
III:2
III:3
III:4
III:5
c.9-1G>A
F2
I:1
I:2
M/+
II:1
M
M: c.9-1G>A
I:2
II:1
NC

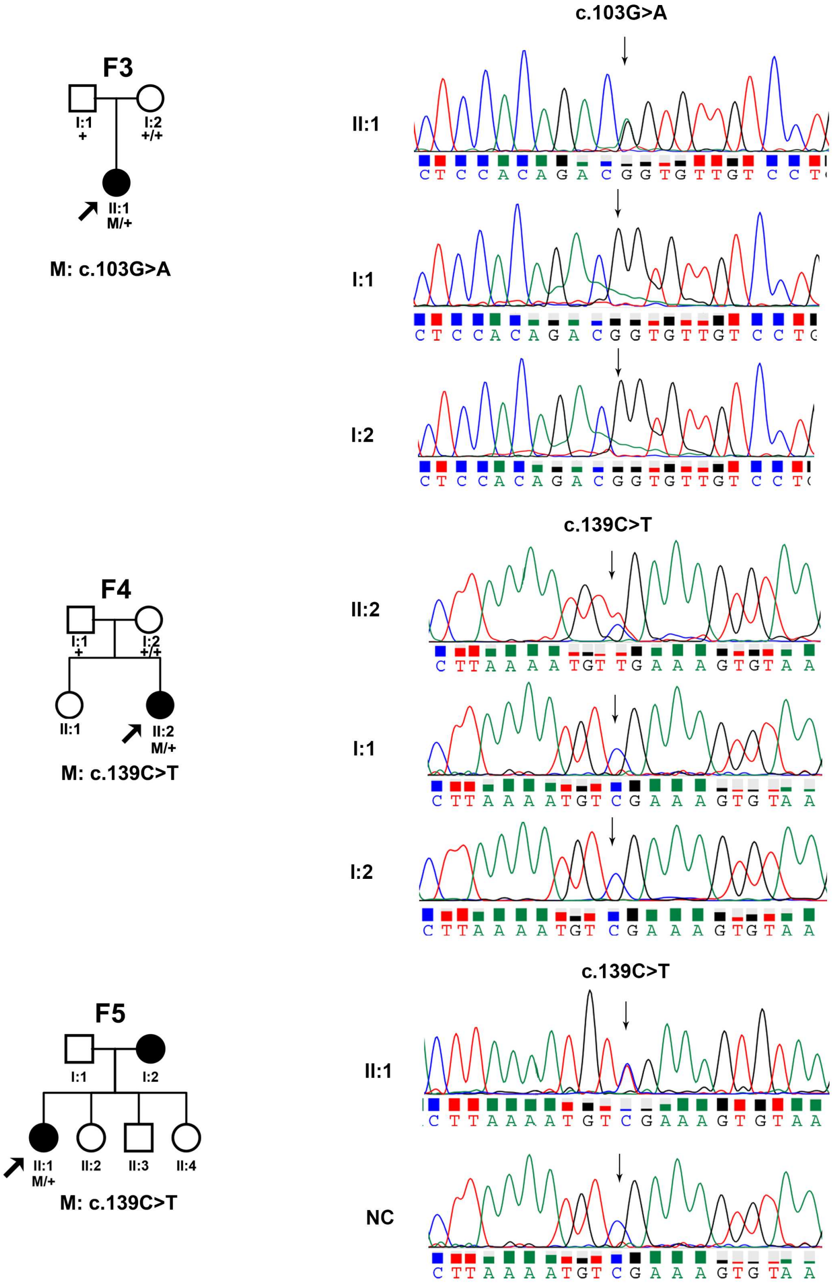
F3
I:1
+
I:2
+/+
II:1
M/+
M: c.103G>A
c.103G>A
II:1
I:1
I:2
F4
I:1
+
I:2
+/+
II:1
II:2
M/+
M: c.139C>T
c.139C>T
II:2
I:1
I:2
F5
I:1
I:2
II:1
M/+
II:2
II:3
II:4
M: c.139C>T
c.139C>T
II:1
NC

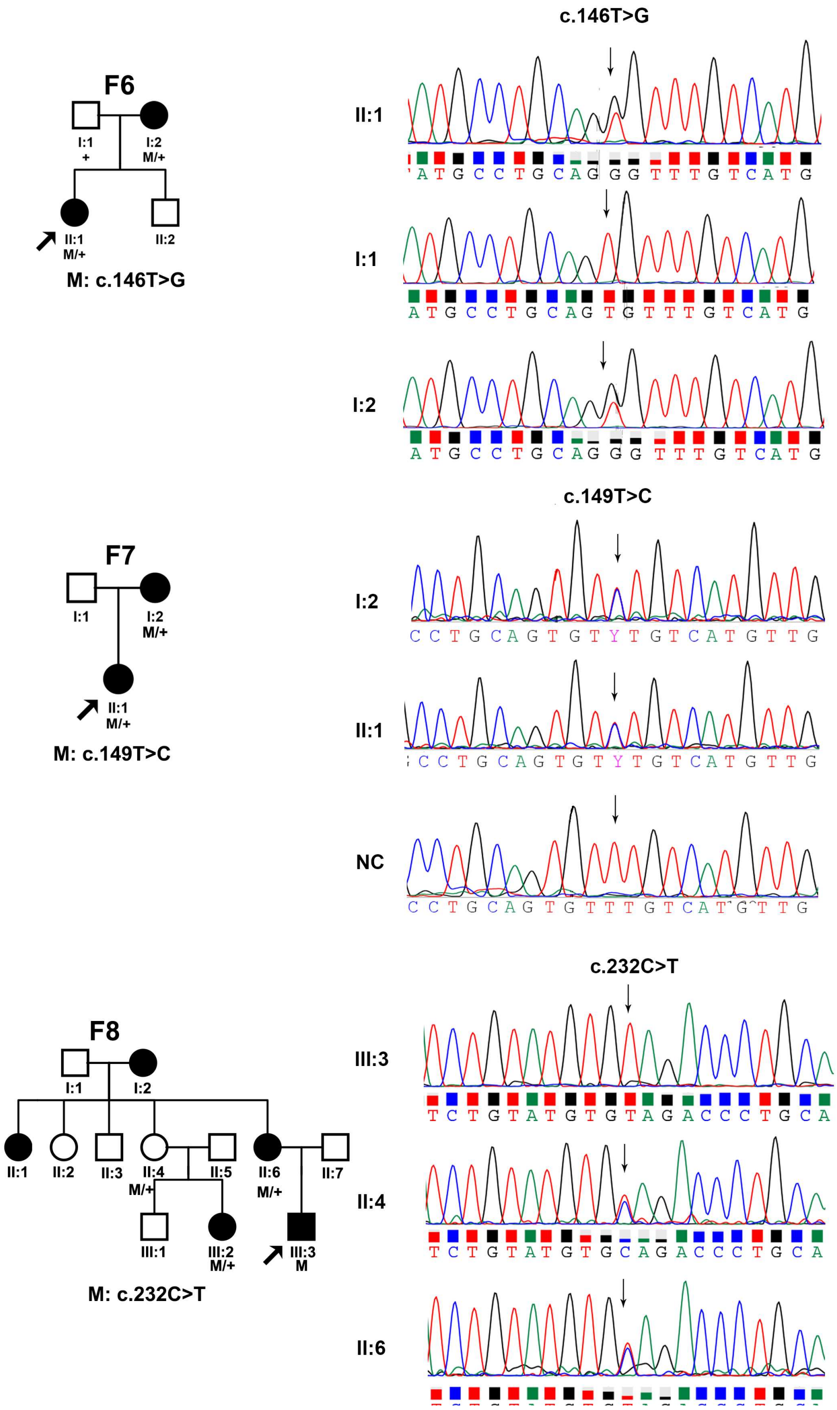
F6
I:1
+
I:2
M/+
II:1
M/+
II:2
M: c.146T>G
c.146T>G
II:1
I:1
I:2
F7
I:1
I:2
M/+
II:1
M/+
M: c.149T>C
c.149T>C
I:2
II:1
NC
F8
I:1
I:2
II:1
II:2
II:3
II:4
M/+
II:5
II:6
M/+
II:7
III:1
III:2
M/+
III:3
M
M: c.232C>T
c.232C>T
III:3
II:4
II:6

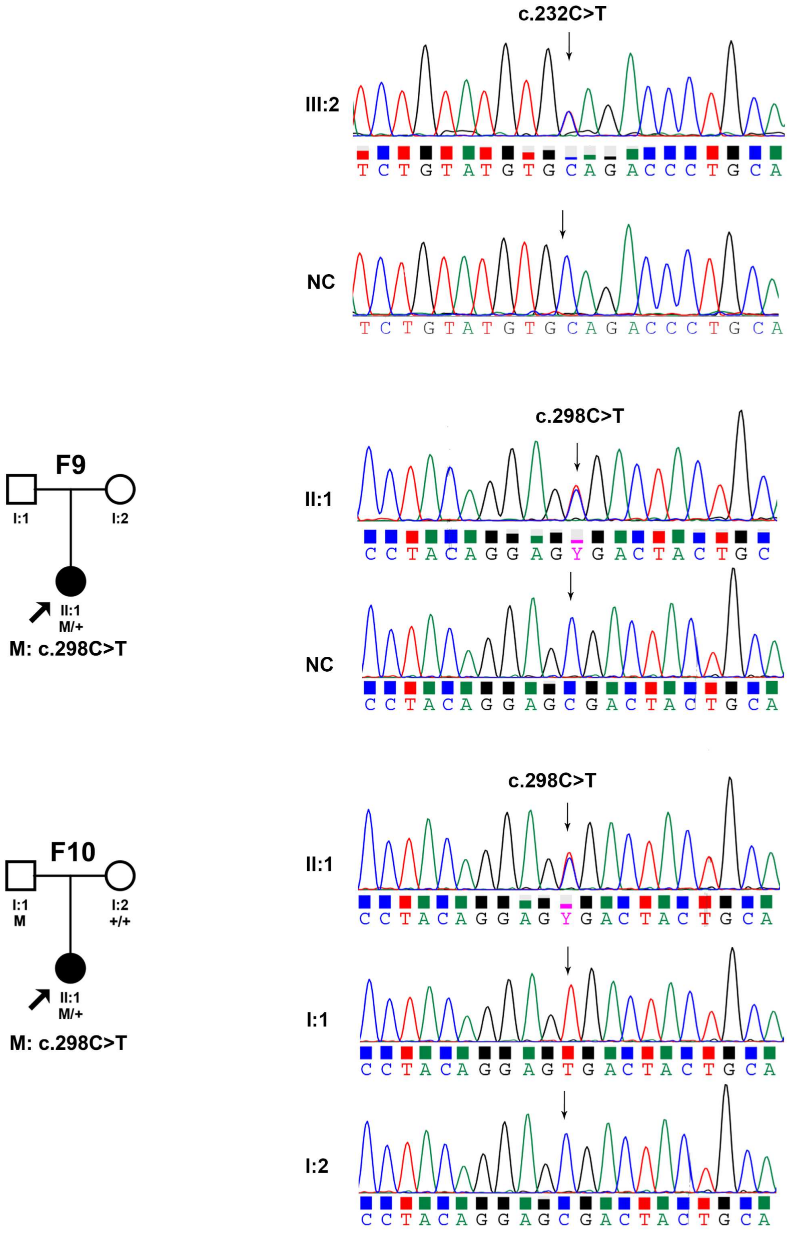
c.232C>T
III:2
TCTGTATGTGCAGACCCTGCA
NC
TCTGTATGTGCAGACCCTGCA
F9
I:1
I:2
II:1
M/+
M: c.298C>T
c.298C>T
II:1
CCTACAGGAGYGACTACTGC
NC
CCTACAGGAGCGACTACTGCA
F10
I:1
M
I:2
+/+
II:1
M/+
M: c.298C>T
c.298C>T
II:1
CCTACAGGAGYGACTACTGCA
I:1
CCTACAGGAGTGACTACTGCA
I:2
CCTACAGGAGCGACTACTGCA

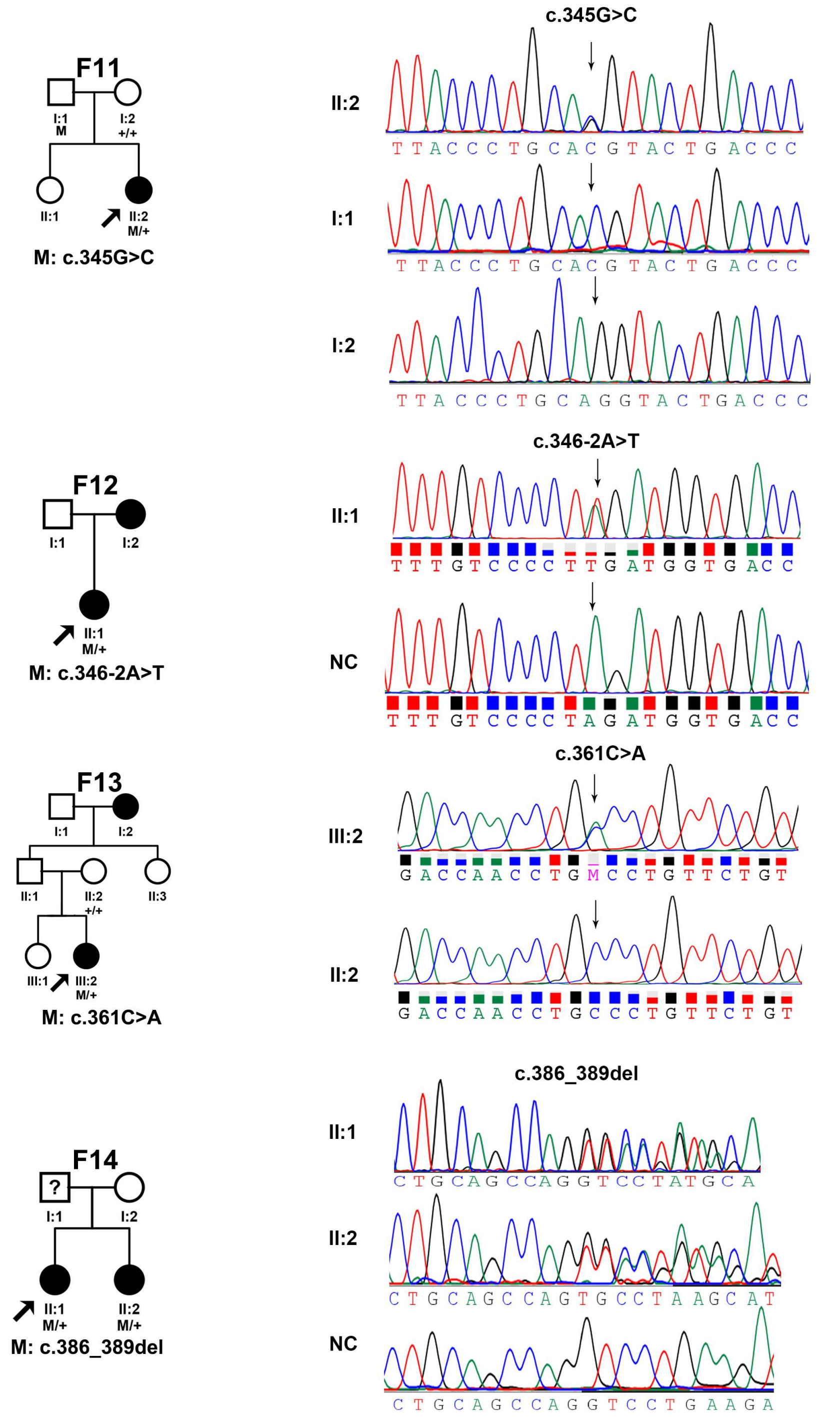
F11
I:1
M
I:2
+/+
II:1
II:2
M/+
M: c.345G>C
c.345G>C
II:2
T T A C C C T G C A C G T A C T G A C C C
I:1
T T A C C C T G C A C G T A C T G A C C C
I:2
T T A C C C T G C A G G T A C T G A C C C
F12
I:1
I:2
II:1
M/+
M: c.346-2A>T
c.346-2A>T
II:1
T T T G T C C C C T T G A T G G T G A C C
NC
T T T G T C C C C T A G A T G G T G A C C
F13
I:1
I:2
II:1
II:2
+/+
II:3
III:1
III:2
M/+
M: c.361C>A
c.361C>A
III:2
G A C C A A C C T G M C C T G T T C T G T
II:2
G A C C A A C C T G C C C T G T T C T G T
F14
?
I:1
I:2
II:1
M/+
II:2
M/+
M: c.386_389del
c.386_389del
II:1
C T G C A G C C A G G T C C T A T G C A
II:2
C T G C A G C C A G T G C C T A A G C A T
NC
C T G C A G C C A G G T C C T G A A G A

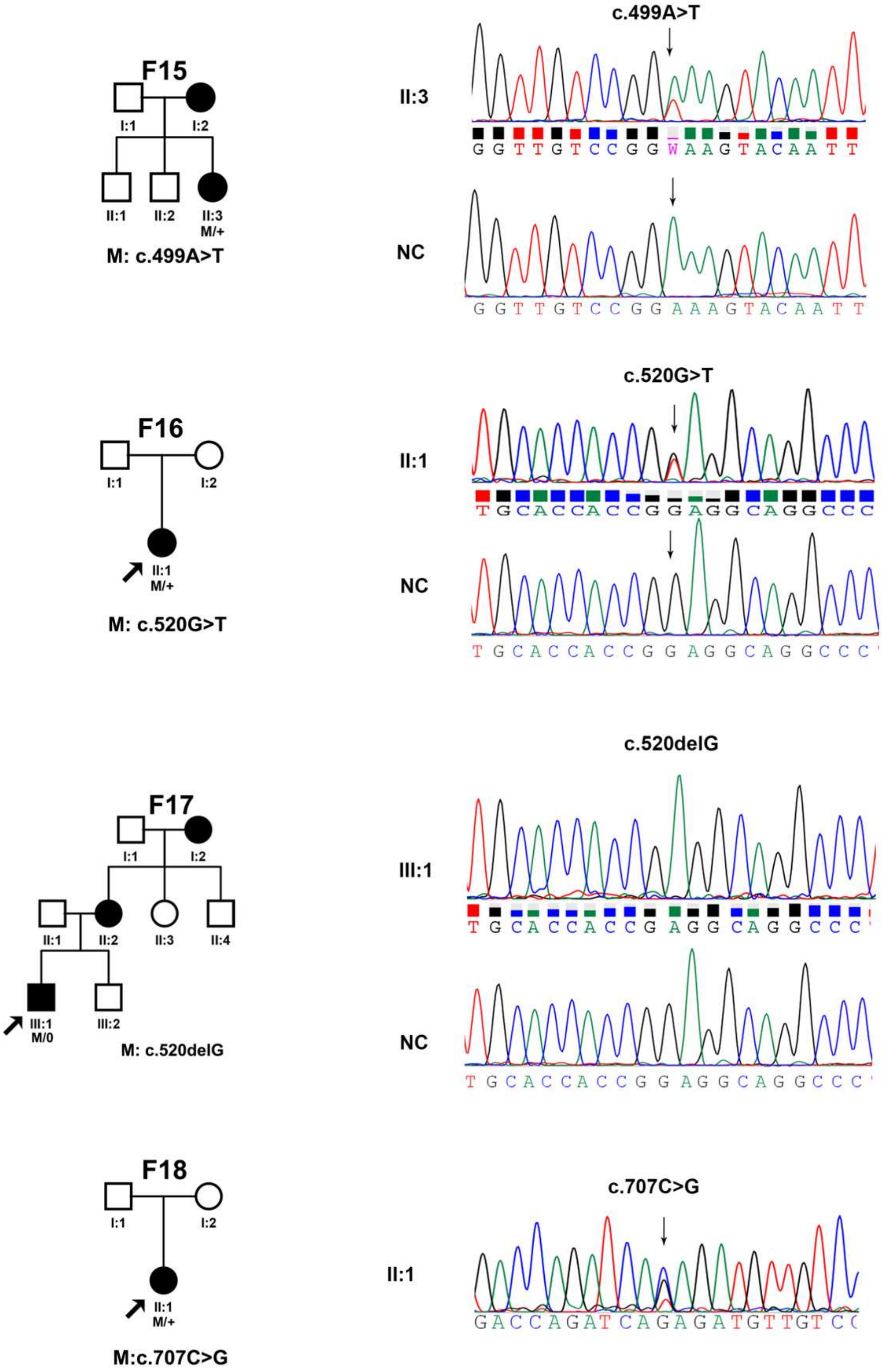
F15
I:1
I:2
II:1
II:2
II:3
M/+
M: c.499A>T
c.499A>T
II:3
G G T T G T C C G G W A A G T A C A A T T
NC
G G T T G T C C G G A A A G T A C A A T T
F16
I:1
I:2
II:1
M/+
M: c.520G>T
c.520G>T
II:1
T G C A C C A C C G G A G G C A G G C C C
NC
T G C A C C A C C G G A G G C A G G C C C
F17
I:1
I:2
II:1
II:2
II:3
II:4
III:1
M/0
III:2
M: c.520delG
c.520delG
III:1
T G C A C C A C C G A G G C A G G C C C
NC
T G C A C C A C C G G A G G C A G G C C C
F18
I:1
I:2
II:1
M/+
M:c.707C>G
c.707C>G
II:1
G A C C A G A T C A G A G A T G T T G T C

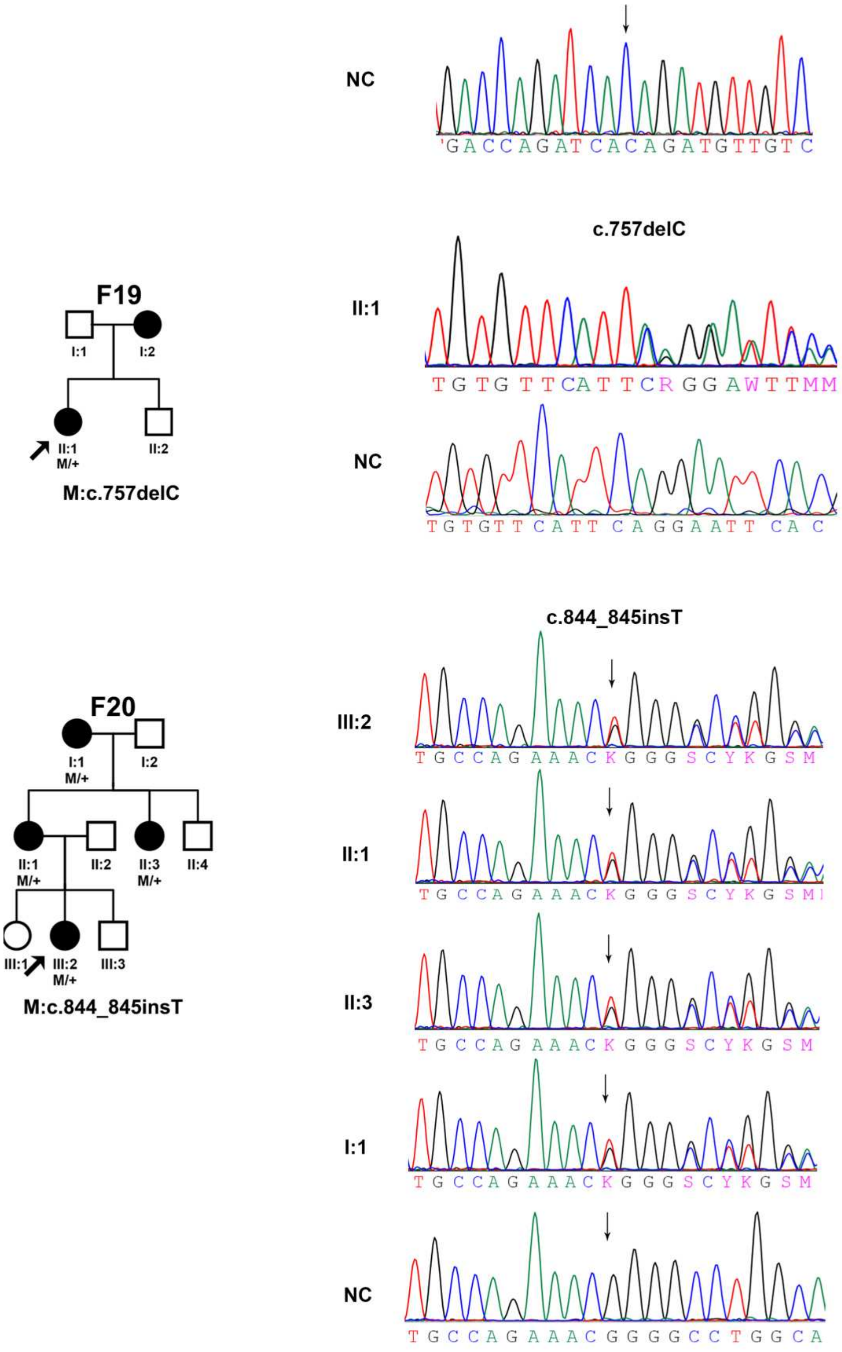
NC
GACCAGATCACAGATGTTGTC
c.757delC
F19
I:1
I:2
II:1
TGTGTTCATTCRGGAWTTMM
NC
TGTGTTCATTCAGGAATTCAC
II:1
M/+
II:2
M:c.757delC
c.844_845insT
F20
III:2
TGCCAGAAACKGGGSCYKGSM
I:1
M/+
I:2
II:1
TGCCAGAAACKGGGSCYKGSM
II:1
M/+
II:2
II:3
M/+
II:4
III:1
III:2
M/+
III:3
II:3
TGCCAGAAACKGGGSCYKGSM
M:c.844_845insT
I:1
TGCCAGAAACKGGGSCYKGSM
NC
TGCCAGAAACGGGGCCTGGCA

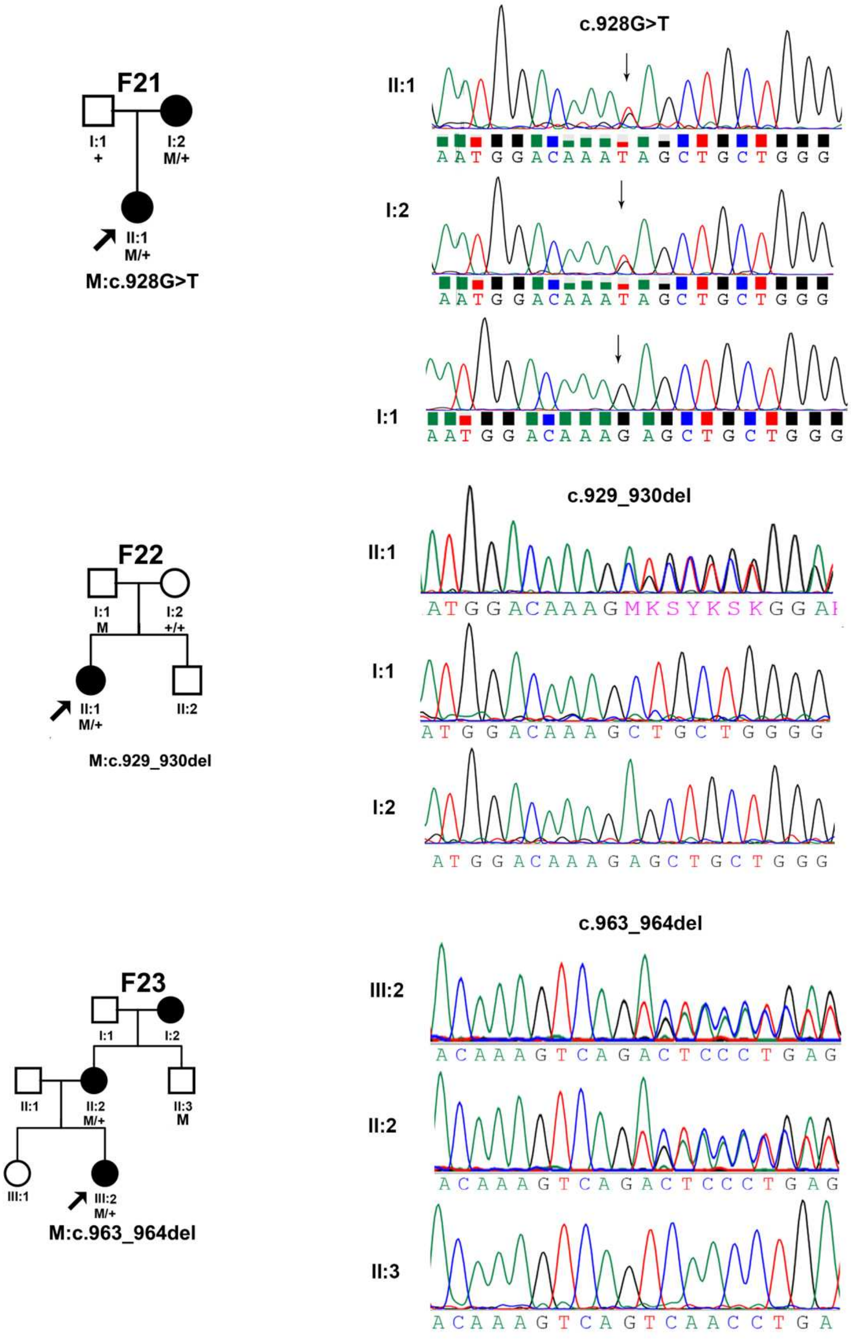
F21
I:1
+
I:2
M/+
II:1
M/+
M:c.928G>T
c.928G>T
II:1
AATGGACAAATAGCTGCTGGG
I:2
AATGGACAAATAGCTGCTGGG
I:1
AATGGACAAAGAGCTGCTGGG
F22
I:1
M
I:2
+/+
II:1
M/+
II:2
M:c.929_930del
c.929_930del
II:1
ATGGACAAAGMKSYKSKGGA
I:1
ATGGACAAAGCTGCTGGGG
I:2
ATGGACAAAGAGCTGCTGGG
F23
I:1
I:2
II:1
II:2
M/+
II:3
M
III:1
III:2
M/+
M:c.963_964del
c.963_964del
III:2
ACAAAGTCAGACTCCCTGAG
II:2
ACAAAGTCAGACTCCCTGAG
II:3
ACAAAGTCAGTCAACCTGA

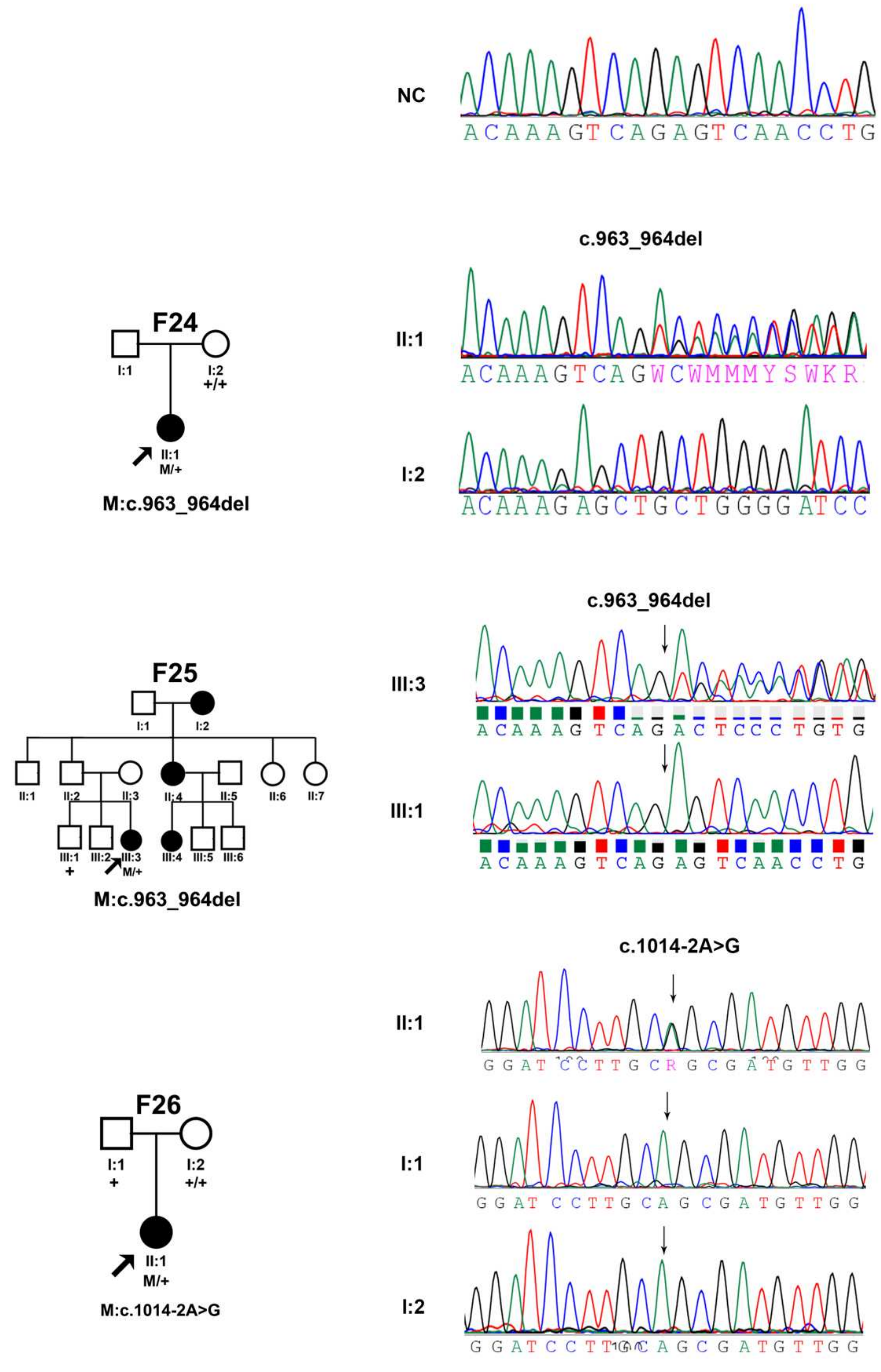


**Supplementary Figure 2.** Sanger sequences and the corresponding pedigrees of 26 newly identified families with pathogenic *ARR3* variants observed in this study.
