# Supplementary material for: Genetic and clinical landscape of ARR3-associated MYP26: the most common cause of Mendelian early-onset high myopia with a unique inheritance
Source: Br J Ophthalmol. 2022 Sep 30;107(10):1545–53. doi: 10.1136/bjo-2022-321511 (PMC10579186; doi:10.1136/bjo-2022-321511)
Supplement: Supplementary data [file bjo-2022-321511supp009.pdf]

Supplementary Table 4. Comparison among X-linked female-limited, X-linked recessive, and X-linked dominant traits.

	X-linked female-limited (XLFL)	X-linked Recessive (XLR)	X-linked dominant (XLD)
Phenotype transmission	from affected females or unaffected males to affected daughters	from unaffected females to affected sons	from affected females to affected sons or daughters; from affected males to affected daughters
Males with mutation	rarely affected	mostly affected	mostly affected
Females with mutation	mostly affected	rarely affected	mostly affected, milder phenotype
Affected genotype	mostly heterozygous	mostly hemizygous	hemizygous or heterozygous
Carrier genotype	hemizygous	heterozygous	none
Representative genes	*PCDH19* , *ARR3*	*CHM*, *RP2* , …	*NHS, GJB1,* …
Male to female ratio	1:20 (*ARR3*)	20:3 (*CHM*)	1:2 (*NHS*)
	1:21(*PCDH19*)	9:1 (*RP2*)	1:2 (*GJB1*)
