# Supplementary material for: Genetic and clinical landscape of ARR3-associated MYP26: the most common cause of Mendelian early-onset high myopia with a unique inheritance
Source: Br J Ophthalmol. 2022 Sep 30;107(10):1545–53. doi: 10.1136/bjo-2022-321511 (PMC10579186; doi:10.1136/bjo-2022-321511)
Supplement: Supplementary data [file bjo-2022-321511supp010.pdf]

Supplementary Table 5. Clinical information of 26 newly identified familes and three reported families with *ARR3* variants.

Family	Mutation	Effect	Dx	Sex	Age (yrs)		RE (OD)		RE (OS)		AL (mm)		BCVA (decimal)		Fundus change				OCT	ERG	
ID					On	Ex	DS	DC	DS	DC	OD	OS	right	left	META	PPA (PD)	Ps	Per		rod	cone
F1-I1	c.3G>A	p.M1?	eoHM	M	EC	74	-20.00	/	-20.00	/	/	/	0.01	0.02	C2	1	II	/	/	/	/
F1-II3	c.3G>A	p.M1?	Un	F	/	51	0.25	-1.25	0.75	-0.25	/	/	0.7	0.9	N	/	/	/	/	/	/
F1-II5	c.3G>A	p.M1?	eoHM	F	EC	41	-10.25	-2.00	-12.00	-0.75	/	/	0.15	0.12	C3	Yes	II	/	/	/	/
F1-II7	c.3G>A	p.M1?	eoHM	F	EC	27	-13.25	-2.25	-3.25	-2.25	/	/	0.4	0.5	C2	1/2	NA	/	/	/	/
F1-III1	c.3G>A	p.M1?	eoHM	F	/	24	-6.25	/	-9.00	/	/	/	0.9	1	C1	Yes	/	/	/	/	/
F1-III2	c.3G>A	p.M1?	eoHM	F	EC	20	-17.00	-1.00	-17.00	2.75	31.76	31.26	0.7	0.9	C2	1/2	II	/	/	MR	MoR
F1-III4	c.3G>A	p.M1?	eoHM	F	EC	8	-13.50	-4.50	-13.50	-4.00	/	/	0.3	0.3	/	/	NA	/	/	/	/
F1-III5	c.3G>A	p.M1?	Un	M	/	4	0.50	-1.00	0.50	-1.00	/	/	/	/	N	/	/	/	/	/	/
F2-II1	c.9-1G>A	SA	eoHM	M	3	6	-7.50	-3.25	-6.00	-0.50	26.41	25.32	0.2-	0.3-	C1	1/2	III	/	N	/	/
F2-I1	c.9-1G>A	SA	eoHM	F	EC	30	-16.50	-3.00	-13.50	-4.75	30.13	28.53	0.3	0.15	C3	2	I	/	D	/	/
F3-II1	c.103G>A	p.G35S	eoHM	F	EC	4	-6.00	-2.00	-8.00	/	/	/	/	/	/	/	/	/	/	/	/
F4-II2	c.139C>T	p.R47*	eoHM	F	EC	8	-8.00	-2.25	-7.75	-3.75	26.06	25.72	0.6	0.5	C1	1/2	III	/	N	/	/
F5-II1	c.139C>T	p.R47*	eoHM	F	EC	47	-20.00	/	-20.00	/	/	/	LP	0.5	C3	3	I	D	D	/	/
F6-I2	c.146T>G	p.L49W	eoHM	F	/	29	-6.12	-1.12	-7.12	-1.25	25.02	25.71	/	/	/	/	/	/	/	/	/
F6-II1	c.146T>G	p.L49W	eoHM	F	EC	7	-5.50	-1.75	-5.50	-0.50	23.52	23.08	0.5	0.5	C1	No	NA	/	/	/	/
F7-I2	c.149T>C	p.F50S	eoHM	F	EC	43	-16.00	-2.00	-14.00	-2.50	29.71	29.08	0.4	0.4	C1	3	II	D	D	/	/

F7-II1	c.149T>C	p.F50S	eoHM	F	7	20	-8.25	-2.50	-9.00	-1.50	28.22	28.73	1	0.9	C0	1/2	/	N	N	N	N
F8-II4	c.232C>T	p.Q78*	Un	F	/	33	-1.00	/	-1.00	-0.75	/	/	1	0.7	NA	NA	NA	/	/	/	/
F8-II6	c.232C>T	p.Q78*	eoHM	F	/	31	-5.50	-0.25	-5.25	-2.25	/	/	0.8	1	NA	NA	NA	/	/	/	/
F8-III2	c.232C>T	p.Q78*	eoHM	F	EC	6	-8.00	-5.00	-8.00	-4.50	/	/	/	/	NA	NA	NA	/	/	/	/
F8-III3	c.232C>T	p.Q78*	eoHM	M	EC	6	-10.50	-3.50	-8.75	-0.75	27.77	27.18	0.4	0.4	C1	1/3	II	/	/	N	MoR
XF3-II1*	c.239T>C	p.L80P	Un	M	/	67	0.50	/	0.50	/	/	/	/	/	NA	NA	NA	/	/	/	/
XF3-III1*	c.239T>C	p.L80P	eoHM	F	EC	45	-20.00	-0.50	-20.25	-0.50	/	29.68	/	0.5	C2	Yes	NA	/	/	/	/
XF3-III3*	c.239T>C	p.L80P	eoHM	F	EC	43	/	/	/	/	/	/	/	/	NA	NA	NA	/	/	/	/
XF3-IV1*	c.239T>C	p.L80P	eoHM	F	EC	27	-14.00	-2.50	-13.00	-2.00	28.26	28.08	0.4	0.4	C1	1/2	III	D	N	/	/
XF3-V1*	c.239T>C	p.L80P	Un	M	/	6	-0.75	-3.25	/	-3.50	23.38	23.04	0.6	0.6	N	/	/	/	N	/	/
XF3-V2*	c.239T>C	p.L80P	eoHM	F	EC	4	-2.25	-5.50	-2.50	-5.00	/	/	0.3	0.3	C0	No	/	N	/	/	/
F9-II1	c.298C>T	p.R100*	eoHM	F	EC	3	-8.00	-5.00	-8.50	-5.25	26.39	26.54	/	/	C1	1/3	/	/	N	/	/
F10-I1	c.298C>T	p.R100*	Un	M	/	/	-3.50	-3.62	-4.00	-3.87	24.18	24.51	/	/	N	/	/	/	N	/	/
F10-II1	c.298C>T	p.R100*	eoHM	F	EC	3	-11.75	-1.50	-10.50	-1.50	24.86	24.58	/	/	C1	1/2	/	/	N	/	/
XF2-II1*	c.298C>T	p.R100*	eoHM	F	EC	58	-14.50	-3.50	-17.25	-1.75	30.18	29.97	0.1	0.1	C2	1	NA	/	/	/	/
XF2-II3*	c.298C>T	p.R100*	eoHM	F	EC	54	-13.00	-2.75	-13.00	-4.75	/	/	0.1	0.2	NA	NA	NA	/	/	/	/
XF2-II5*	c.298C>T	p.R100*	eoHM	F	EC	48	-6.75	-1.75	-4.00	-1.50	25.55	24.76	0.1	0.4	/	NA	NA	/	/	/	/
XF2-II9*	c.298C>T	p.R100*	eoHM	F	EC	43	-18.75	-0.75	-18.75	-1.75	31.25	31.48	0.3	0.4	/	NA	NA	/	/	/	/
XF2-III1*	c.298C>T	p.R100*	eoHM	F	EC	31	-12.00	-2.50	-13.25	-2.75	29.37	29.95	0.5	0.4	C2	1	II	/	N	N	MR

XF2-III5*	c.298C>T	p.R100*	eoHM	F	EC	26	-10.75	-3.75	-11.50	-2.75	27.53	27.30	0.5	0.5	C1	1	II	/	/	/	/
XF2-III6*	c.298C>T	p.R100*	eoHM	F	EC	14	-8.00	-2.00	-7.75	-4.50	25.84	26.19	0.4	0.2	C1	No	NA	/	/	/	/
XF2-III9*	c.298C>T	p.R100*	eoHM	F	EC	19	-13.25	-2.50	-14.25	-1.50	27.83	27.95	0.3	0.3	C1	1/2	III	/	N	/	/
XF2-III10*	c.298C>T	p.R100*	eoHM	F	EC	13	-16.00	-4.50	-16.50	-4.50	28.89	28.99	0.4	0.4	C1	1	II	/	N	N	MoR
XF2-IV1*	c.298C>T	p.R100*	eoHM	F	/	4	-5.50	-5.25	-2.50	-2.50	25.11	24.57	/	/	C0	No	III	N	/	/	/
F11-I1	c.345G>C	p.Q115H	Un	M	/	/	-0.50	/	-0.50	/	/	/	/	/	N	/	/	/	N	/	/
F11-II2	c.345G>C	p.Q115H	eoHM	F	2	3	-9.00	-1.50	-9.00	-2.75	24.55	24.96	0.7	0.5	C1	No	II	N	N	MoR	MoR
F12-II1	c.346-2A>T	SA	eoHM	F	/	8	-17.25	-5.25	-14.25	-5.00	30.70	29.91	0.12	0.7	C1	1	III	/	N	/	/
F13-III2	c.361C>A	p.P121T	eoHM	F	EC	2	-9.50	-2.00	-10.50	-1.75	24.48	24.45	/	/	C0	1/2	/	/	/	/	/
F14-II1	c.386_389del	p.G129Vfs*14	eoHM	F	EC	6	-8.50	-2.00	-8.25	-2.00	/	/	0.4	0.4	NA	NA	NA	/	/	/	/
F15-II3	c.499A>T	p.K167*	eoHM	F	EC	9	-17.50	-2.50	-14.50	-2.50	29.46	28.39	0.5	0.5	C1	No	III	/	N	N	MoR
F16-II1	c.520G>T	p.E174*	eoHM	F	EC	10	-12.00	-3.00	-11.25	-3.50	27.24	26.99	1	0.8	NA	NA	NA	/	/	/	/
F17-III1	c.520delG	p.E174Rfs*70	eoHM	M	EC	7	-4.00	-2.00	-5.50	-2.50	25.53	26.65	0.4	0.3	C1	No	/	/	N	/	/
F18-II1	c.707C>G	p.T236R	eoHM	F	3	4	-8.00	-3.50	-7.50	-3.00	/	/	0.5	0.5	C0	No	NA	/	/	/	/
F19-II1	c.757delC	p.Q253Rfs*7	eoHM	F	EC	5	-5.25	-5.50	-6.25	-6.00	/	/	0.15	0.15	C1	1/2	III	/	N	/	/
F20-III2	c.844_845insT	p.R282Lfs*10	eoHM	F	2	3	-15.00	-2.50	-15.50	-1.75	27.15	27.30	0.15	0.15	C1	1/3	III	N	N	/	/
F20-II1	c.844_845insT	p.R282Lfs*10	eoHM	F	7	26	-13.00	-1.00	-13.00	-0.62	28.20	28.10	0.7	0.8	C1	1	II	D	N	MR	MR
XF1-III1*	c.893C>A	p.A298D	Un	M	/	78	-0.25	-1.00	-0.50	-1.00	22.64	22.57	0.4	0.4	N	/	/	/	/	/	/
XF1-III3*	c.893C>A	p.A298D	Un	M	/	72	-1.25	-1.25	-2.00	-1.50	23.03	22.90	0.4	0.4	N	/	/	/	/	/	/

XF1-III7*	c.893C>A	p.A298D	eoHM	F	EC	79	-15.00	-4.00	-14.75	-6.00	28.32	29.23	0.01	FC	C3	Yes	NA	/	/	/	/
XF1-IV2*	c.893C>A	p.A298D	eoHM	F	/	59	-14.25	-3.25	/	/	28.50	29.20	/	/	NA	NA	NA	/	/	/	/
XF1-IV4*	c.893C>A	p.A298D	eoHM	F	EC	57	-7.50	-2.25	-6.50	-3.25	25.59	25.25	FC	0.5	C3	2	V	/	/	/	/
XF1-IV6*	c.893C>A	p.A298D	eoHM	F	EC	54	/	/	/	/	27.91	28.24	0.4	0.4	C1	Yes	NA	/	/	/	/
XF1-IV7*	c.893C>A	p.A298D	eoHM	F	EC	45	/	/	/	/	28.10	27.01	0.4	0.4	C1	Yes	NA	/	/	/	/
XF1-IV8*	c.893C>A	p.A298D	eoHM	F	EC	41	-15.50	-3.25	-16.00	-4.75	29.08	28.76	0.4	0.4	C1	Yes	NA	/	/	/	/
XF1-IV12*	c.893C>A	p.A298D	eoHM	F	EC	48	/	/	/	/	31.29	30.88	0.15	0.8	NA	NA	NA	/	/	/	/
XF1-IV13*	c.893C>A	p.A298D	eoHM	F	EC	45	/	/	/	/	29.92	30.83	0.3	1	C3	2	NA	/	/	/	/
XF1-IV15*	c.893C>A	p.A298D	eoHM	F	EC	40	-16.75	-3.00	-14.75	-2.50	29.65	28.92	0.2	0.2	C1	3	II	/	N	MoR	MoR
XF1-V2*	c.893C>A	p.A298D	eoHM	F	EC	34	-19.75	-1.00	-18.25	-1.50	30.88	30.61	0.8	1.2	C1	2	I	/	/	/	/
XF1-V3*	c.893C>A	p.A298D	eoHM	F	EC	34	/	/	/	/	29.67	29.66	0.4	1	C1	1	I	/	/	/	/
XF1-V11*	c.893C>A	p.A298D	eoHM	F	EC	24	-9.00	-2.50	-19.25	-0.50	33.45	31.13	0.08	0.4	C1	1	I	/	/	/	/
XF1-V12*	c.893C>A	p.A298D	eoHM	F	EC	4	-12.00	-2.50	-5.00	-1.00	/	/	0.2	0.3	C1	No	NA	/	N	/	/
XF1-VI1*	c.893C>A	p.A298D	eoHM	F	EC	10	-7.25	-6.00	-8.00	-5.25	26.68	26.50	1	1	C1	1/2	III	/	/	/	/
XF1-VI4*	c.893C>A	p.A298D	eoHM	F	/	2	-5.75	-0.75	-5.75	-0.75	24.04	23.45	/	/	C0	No	NA	/	/	/	/
F21-I1	c.928G>T	p.E310*	eoHM	F	EC	30	-28.00	-1.50	-21.50	-2.50	34.09	32.09	0.1	0.1	C3	1	I	/	D	/	/
F21-II1	c.928G>T	p.E310*	eoHM	F	EC	5	-10.50	-2.00	-9.75	-2.00	27.08	26.87	0.2	0.2	NA	NA	NA	/	/	/	/
F22-I1	c.929_930del	p.E310Afs*46	Un	M	/	34	-2.50	-1.75	-2.50	-1.00	24.10	24.01	/	/	N	/	/	/	/	/	/
F22-II1	c.929_930del	p.E310Afs*46	eoHM	F	/	2	-6.00	-0.75	-5.75	-1.00	24.44	24.28	0.3	0.3	C1	1/3	III	N	/	/	/

F23-II2	c.963_964del	p.R321Sfs*35	eoHM	F	EC	32	-7.00	-1.25	-6.62	-3.00	26.89	26.43	0.63	0.5	C1	1/3	III	D	N	N	MR
F23-III2	c.963_964del	p.R321Sfs*35	eoHM	F	EC	1	1.00	-1.00	-1.50	-1.25	/	/	/	/	C1	1/2	III	N	N	/	/
F24-II1	c.963_964del	p.R321Sfs*35	eoHM	F	4	5	-9.00	-1.25	-9.00	-4.00	26.77	26.69	0.4	0.3	C1	1/2	/	/	N	N	MoR
F25-III3	c.963_964del	p.R321Sfs*35	eoHM	F	EC	17	-18.25	-2.50	-16.00	-3.50	/	/	0.2	0.6	C1	1	V	/	N	N	N
F26-II1	c.1014-2A>G	SA	eoHM	F	EC	3	-12.75	-2.25	-11.75	-2.50	27.17	26.61	0.3	0.3	C1	No	II	N	N	N	N

Abbreviations: AL = axial length; ID = Identification; Dx = Diagnosis; RE = Refractive error; BCVA = Best corrected visual acuity; On = Onset; Ex = Exam; DS = Diopter Spherical; DC = Diopter Cylinder; OD = Oculus Dexter; OS = Oculus Sinister; PPA = Peripapillary atrophy; PD = Papillary diameter; Ps = Posterior staphyloma; Per. = Peripheral; OCT = Optical coherence tomography; ERG = Electroretinogram; eoHM = Early onset high myopia; Un = Unaffected; M = Male; F = Female; EC = early childhood; LP = Light perception; FC =Finger counts; N = Normal; NA = Not available; D = degeneration; MR = mildly reduced; MoR= Moderately reduced.
Fundus change: C0: No macular lesions; C1: Tessellated fundus; C2: Diffuse chorioretinal atrophy; C3: Patchy chorioretinal atrophy; The posterior fundus change was classified according to the META-PM study, and the classification of poeterior staphyloma was referring to the Curtin's classification.
*indicated families which have been reported in our previous study.
