# Supplementary material for: Genetic and clinical landscape of ARR3-associated MYP26: the most common cause of Mendelian early-onset high myopia with a unique inheritance
Source: Br J Ophthalmol. 2022 Sep 30;107(10):1545–53. doi: 10.1136/bjo-2022-321511 (PMC10579186; doi:10.1136/bjo-2022-321511)
Supplement: Supplementary data [file bjo-2022-321511supp003.pdf]

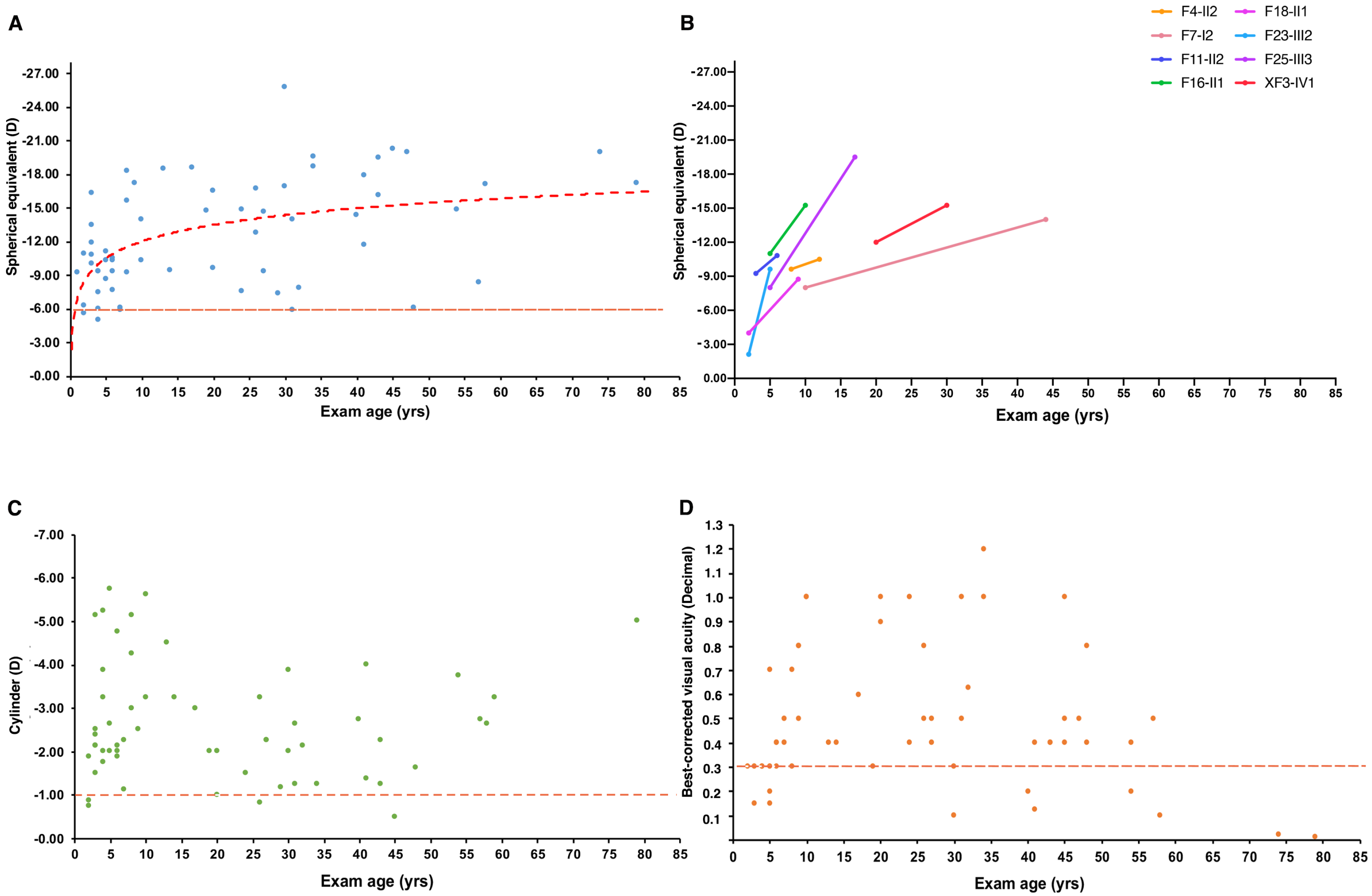

Supplementary Figure 3. The refraction distribution, refraction progression, and best-corrected visual acuity distribution of patients with pathogenic *ARR3* variants. (A) The scatter plot shows the spherical equivalent diopter distribution of 62 patients with available refraction data. (B) The line chart shows the refraction progression of eight follow-up patients. (C) The scatter diagram shows the cylinder diopter distribution of patients with *ARR3*-associated MYP26. (D) The scatter plot shows the best-corrected visual acuity distribution of 57 patients with available data.
