# Supplementary material for: Genetic and clinical landscape of ARR3-associated MYP26: the most common cause of Mendelian early-onset high myopia with a unique inheritance
Source: Br J Ophthalmol. 2022 Sep 30;107(10):1545–53. doi: 10.1136/bjo-2022-321511 (PMC10579186; doi:10.1136/bjo-2022-321511)
Supplement: Supplementary data [file bjo-2022-321511supp004.pdf]

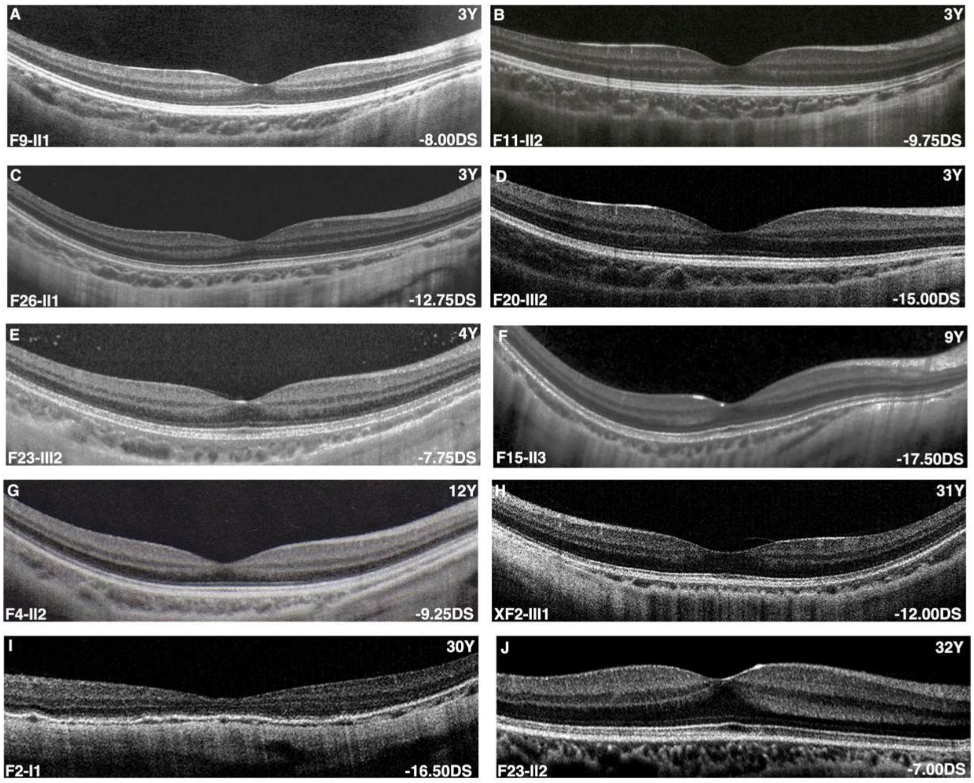

Supplementary Figure 4. The OCT scans of patients with pathogenic variants in *ARR3* in our cohort. (A-H) Most patients presented with preservation structure of macular fovea. (I) Some adult patients with *ARR3*-assocaited MYP26 show myopic macular dystrophy, especially atrophic sensory retinas. (J) One 32-year-old affected female presented with slightly shallow fovea. The top-right number shows the examined age of each OCT scan (Y: years old), while the refraction data was in the low-right of each image.
