# Supplementary material for: Genetic and clinical landscape of ARR3-associated MYP26: the most common cause of Mendelian early-onset high myopia with a unique inheritance
Source: Br J Ophthalmol. 2022 Sep 30;107(10):1545–53. doi: 10.1136/bjo-2022-321511 (PMC10579186; doi:10.1136/bjo-2022-321511)
Supplement: Supplementary data [file bjo-2022-321511supp005.pdf]

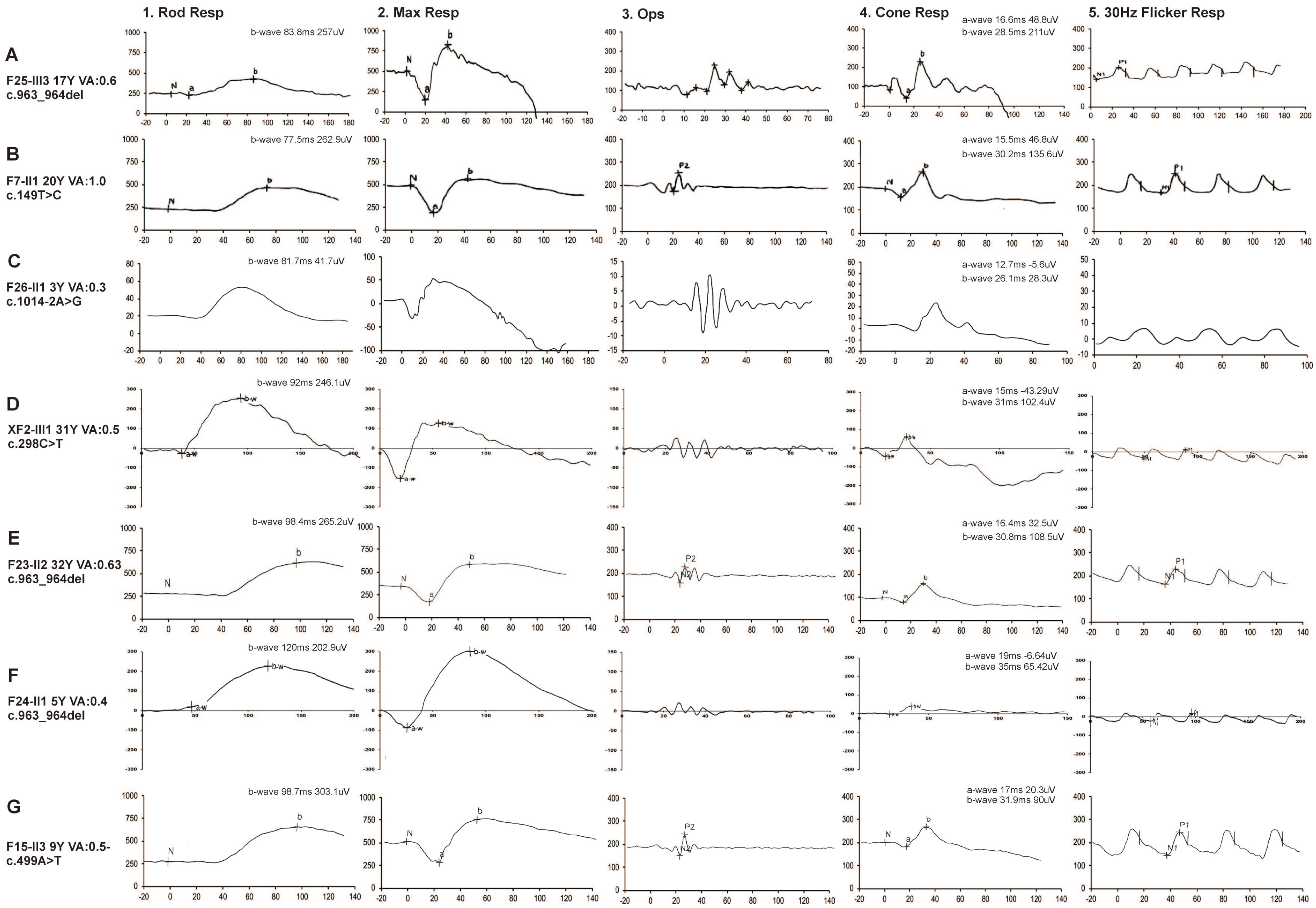
1. Rod Resp
2. Max Resp
3. Ops
4. Cone Resp
5. 30Hz Flicker Resp
A
F25-III3 17Y VA:0.6
c.963_964del
b-wave 83.8ms 257uV
a-wave 16.6ms 48.8uV
b-wave 28.5ms 211uV
B
F7-II1 20Y VA:1.0
c.149T>C
b-wave 77.5ms 262.9uV
a-wave 15.5ms 46.8uV
b-wave 30.2ms 135.6uV
C
F26-II1 3Y VA:0.3
c.1014-2A>G
b-wave 81.7ms 41.7uV
a-wave 12.7ms -5.6uV
b-wave 26.1ms 28.3uV
D
XF2-III1 31Y VA:0.5
c.298C>T
b-wave 92ms 246.1uV
a-wave 15ms -43.29uV
b-wave 31ms 102.4uV
E
F23-II2 32Y VA:0.63
c.963_964del
b-wave 98.4ms 265.2uV
a-wave 16.4ms 32.5uV
b-wave 30.8ms 108.5uV
F
F24-II1 5Y VA:0.4
c.963_964del
b-wave 120ms 202.9uV
a-wave 19ms -6.64uV
b-wave 35ms 65.42uV
G
F15-II3 9Y VA:0.5-
c.499A>T
b-wave 98.7ms 303.1uV
a-wave 17ms 20.3uV
b-wave 31.9ms 90uV

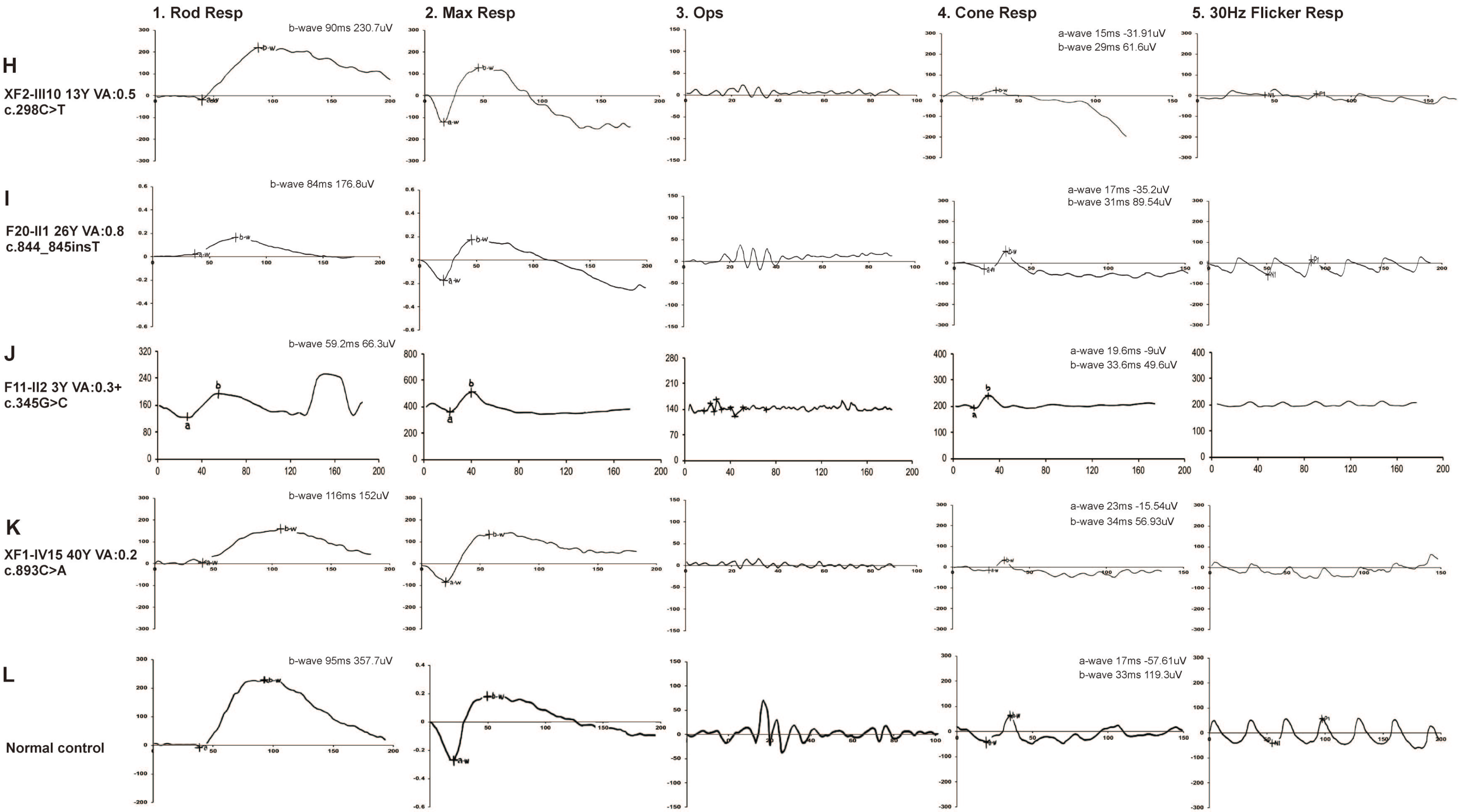

Supplementary Figure 5. Electroretinogram recordings of 11 representative patients with pathogenic *ARR3* variants. Three patients (A-C) had normal rod and cone responses, two patients (D-E) had normal rod responses and mildly reduced a-/b- wave amplitude in cones, three patients (F-H) had normal rod responses and moderately reduced a-/b- wave amplitude in cones, one patient (I) had mildly reduced a-/b- wave amplitude in both rods and cones, and two patients (J-K) had moderately reduced a-/b- wave amplitude in both rods and cones, (L) normal control.
